# Endogenous authentic OCT4A proteins directly regulate FOS/AP-1 transcription in somatic cancer cells

**DOI:** 10.1038/s41419-018-0606-x

**Published:** 2018-05-22

**Authors:** Yanwen Zhou, Xinyu Chen, Bo Kang, Shiqi She, Xiaobing Zhang, Cheng Chen, Wenxin Li, Wenjie Chen, Songsong Dan, Xiaoyun Pan, Xiaoli Liu, Jianqin He, Qingwei Zhao, Chenggang Zhu, Ling Peng, Haoyi Wang, Hangping Yao, Hongcui Cao, Lanjuan Li, Meenhard Herlyn, Ying-Jie Wang

**Affiliations:** 1 State Key Laboratory for Diagnosis and Treatment of Infectious Diseases, Collaborative Innovation Center for Diagnosis and Treatment of Infectious Diseases, the First Affiliated Hospital, School of Medicine, Zhejiang University, Hangzhou, Zhejiang 310003 China; 2 Department of Infectious Diseases, the Second Xiangya Hospital, Central South University, Changsha, Hunan 410011 China; 3 College of Life Sciences, Zhejiang University, Hangzhou, Zhejiang 310058 China; 4 The First Affiliated Hospital, School of Medicine, Zhejiang University, Hangzhou, Zhejiang 310003 China; 5 Department of Radiotherapy, the First Affiliated Hospital, School of Medicine, Zhejiang University, Hangzhou, Zhejiang 310003 China; 6 State Key Laboratory of Stem Cell and Reproductive Biology, Institute of Zoology, Chinese Academy of Sciences, 100101 Beijing, China; 7 Molecular and Cellular Oncogenesis Program and Melanoma Research Center, The Wistar Institute, Philadelphia, PA 19104 USA

## Abstract

OCT4A is well established as a master transcription factor for pluripotent stem cell (PSC) self-renewal and a pioneer factor for initiating somatic cell reprogramming, yet its presence and functionality in somatic cancer cells remain controversial and obscure. By combining the CRISPR-Cas9-based gene editing with highly specific PCR assays, highly sensitive immunoassays, and mass spectrometry, we provide unequivocal evidence here that full-length authentic OCT4A transcripts and proteins were both present in somatic cancer cells, and OCT4A proteins were heterogeneously expressed in the whole cell population and when expressed, they are predominantly localized in cell nucleus. Despite their extremely low abundance (approximately three orders of magnitude lower than in PSCs), OCT4A proteins bound to the promoter/enhancer regions of the AP-1 transcription factor subunit c-FOS gene and critically regulated its transcription. Knocking out OCT4A in somatic cancer cells led to dramatic reduction of the c-FOS protein level, aberrant AP-1 signaling, dampened self-renewal capacity, deficient cell migration that were associated with cell growth retardation in vitro and in vivo, and their enhanced sensitivity to anticancer drugs. Taken together, we resolve the long-standing controversy and uncertainty in the field, and reveal a fundamental role of OCT4A protein in regulating FOS/AP-1 signaling-centered genes that mediate the adhesion, migration, and propagation of somatic cancer cells.

## Introduction

*POU5F1* gene belongs to the class 5 POU (Pit-Oct-Unc) family of homeodomain transcription factors (TFs) whose transcript can generate three main isoforms by alternative splicing, namely OCT4A (often referred to as OCT4), OCT4B, and OCT4B1^1^. OCT4A is by far the most studied isoform given its crucial roles in early development^2^, pluripotent stem cell (PSC) maintenance^3^, and somatic cell reprogramming^4–6^. Human OCT4A protein has 360 amino acids and consists of an N-transactivation domain, a POU domain, and a C-transactivation domain^7^. POU domain can bind the canonical octamer motif (ATGCA/TAAT) through which OCT4A recognizes the promoter or enhancer regions of its hundreds of target genes and regulates their transcription^8^. Together with SOX2 and NANOG, OCT4A maintains the pluripotency and self-renewal of PSCs mainly by activating the pluripotency genes and suppressing the lineage-specific genes^3,8–10^.

Studies in PSC self-renewal and somatic cell reprogramming indicated that an optimally intermediate level of OCT4A is associated with maximal stemness or pluripotency^11,12^. During gastrulation, the transcription of OCT4A is thought to be irreversibly turned off by DNA-methylation-based epigenetic mechanism^13^, and therefore, it is generally thought that OCT4A is not expressed in normal somatic cells^8,13^. On the other hand, a large body of literature claimed the detection of OCT4A mRNAs and proteins in a variety of differentiated cancer cell lines, cancer tissues, and normal adult stem cells, implicating its crucial roles in the initiation and development of various human cancers^7,14–19^. However, main caveats exist in those studies that include: the possible presence of other OCT4 isoforms and multiple *POU5F1* pseudogenes that cannot be effectively distinguished by most PCR primers^20–22^; commercially available OCT4 antibodies cannot ensure their specific detection of OCT4A protein only^7,22,23^. Considerable efforts have been made by shRNA/siRNA approach in order to verify or validate the presence and functionality of OCT4A in somatic cancer cells^24,25^. However, shRNA/siRNA approach can only provide incomplete gene silencing, leaving residual OCT4 mRNAs and proteins that may still function; furthermore, it has relatively high off-target effects that cannot eliminate possible indirect contributions from reducing *POU5F1* pseudogenes.

Since neither full-length OCT4A transcripts nor full-length OCT4A proteins in somatic cancer cells have been identified or verified by unequivocal means (e.g., DNA sequencing, mass spectrometry (MS)) so far, what we can conclude from the literature was that certain *POU5F1* transcripts or other POU family member transcripts may be expressed in somatic cancer cells and/or a subpopulation of cancer cells known as cancer stem cells (CSCs) or tumor initiating cells (TICs). Despite numerous reports, it still remains unsolved questions in the field: are endogenous authentic OCT4A proteins truly present in any somatic cancer cells? What are the bona fide target genes and functional roles of OCT4A in somatic cancer cells? In this study, by combining CRISPR-Cas9-based gene editing with highly specific PCR assays, highly sensitive immunoassays, and MS approaches, we provide definitive answers and novel insights to these long-sought questions.

## Results

### Full-length authentic OCT4A transcripts were detected in somatic cancer cells

**Fig. 1:**
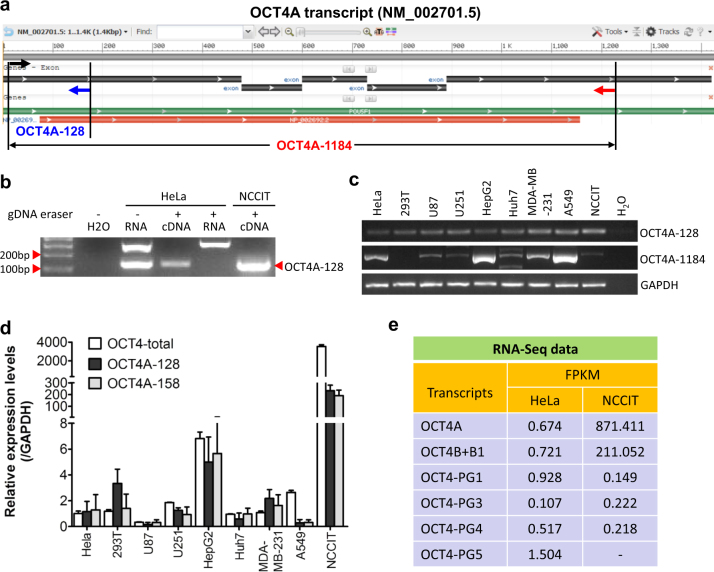
Identification and quantitation of endogenous authentic OCT4A transcripts in somatic cancer cells. **a** The target locations of OCT4A-128 and OCT4A-1184 primers relative to the *POU5F1* gene. **b** Determination of the efficiency of gDNA elimination mediated by gDNA eraser in the PrimeScript RT reagent kit. Total RNAs of HeLa cells treated with or without gDNA eraser and HeLa cDNAs reverse transcribed from total RNAs treated with gDNA eraser were subjected to PCR analysis. NCCIT cDNAs were used as a positive control and no template (H_2_O) as a negative control. **c** Detection of a small fragment (OCT4A-128) and the full-length (OCT4A-1184) transcript of OCT4A in multiple somatic cancer or normal cell lines by RT-PCR. For NCCIT, the amounts of PCR products of OCT4A-128 and OCT4A-1184 loaded were only 1/70 of other cells. **d** qRT-PCR analysis of the cell lines in (**c**), with OCT4A-total, OCT4A-128, and OCT4A-158 primers, GAPDH was used as an internal control. **e** FPKM of transcripts of *POU5F1* and *POU5F1* pseudogenes in HeLa and NCCIT cells

Several studies have previously detected OCT4A-specific transcript fragments in somatic cancer cells that were confirmed by DNA sequencing^20,26,27^. However, due to alternative splicing or even contamination of genomic DNA, positive signals of short transcript fragments cannot guarantee the presence of the full-length transcripts. We therefore carefully designed two pairs of OCT4A-specific primers that share identical forward primer targeting the 5′-UTR region of exon 1 that is absent from other known OCT4 isoforms and all known pseudogenes, named OCT4A-128 and OCT4-1184 (Fig. 1a; Supplementary Figure 1A). First, a PCR was conducted to assess the efficiency of residual gDNA elimination, and further DNA sequencing confirmed that the OCT4A-128 bands were truly amplified from the fragments of OCT4A transcripts in HeLa cells (Fig. 1b; Supplementary Figure 1B). Then, RT-PCR analyses showed that the OCT4A-128 band was detected in all the examined cells that include a non-tumor cell line (293T), seven human somatic cancer cell lines and a human embryonal carcinoma cell line (NCCIT, as positive control) (Fig. 1c, upper panel). However, the full-length OCT4A-1184 band variably appeared in somatic cancer cells but missed in all non-tumor samples including 293T cells (Fig. 1b, middle panel), LO2 cells, and normal human liver tissues with the exception of HUVEC cells (Supplementary Figure 1C) whose identity was subsequently confirmed by DNA sequencing (Supplementary Figure 1D, E). Next, we conducted qRT-PCR using three primer pairs: the “OCT4A-128”, a well-characterized OCT4A-specific primer pair (“OCT4-158”)^27^ (Supplementary Figure 1A), and a previously used primer pair that can amplify all known OCT4 isoforms and major *POU5F1* pseudogenes (“OCT4-total”)^25^. The three primer pairs gave similar quantitative results, showing that the OCT4A mRNA levels in somatic cancer cells were 2−3 orders of magnitude lower than that in NCCIT (Fig. 1d). The RNA-Seq data also revealed that the transcript levels of other known OCT4 isoforms and major *POU5F1* pseudogenes were similar to that of OCT4A in HeLa cells and the OCT4A mRNA level difference between HeLa and NCCIT was in line with that of qRT-PCR (Fig. 1e). Moreover, a full-length OCT4A transcript could be assembled based on RNA-Seq reads from HeLa cells (Supplementary Figure 2), further supporting our RT-PCR finding for the presence of the full-length OCT4A transcripts. Collectively, we concluded that full-length authentic OCT4A mRNAs were present at low levels in somatic cancer cells.

### Circumstantial proofs for the presence and identity of authentic OCT4A proteins in somatic cancer cells

**Fig. 2:**
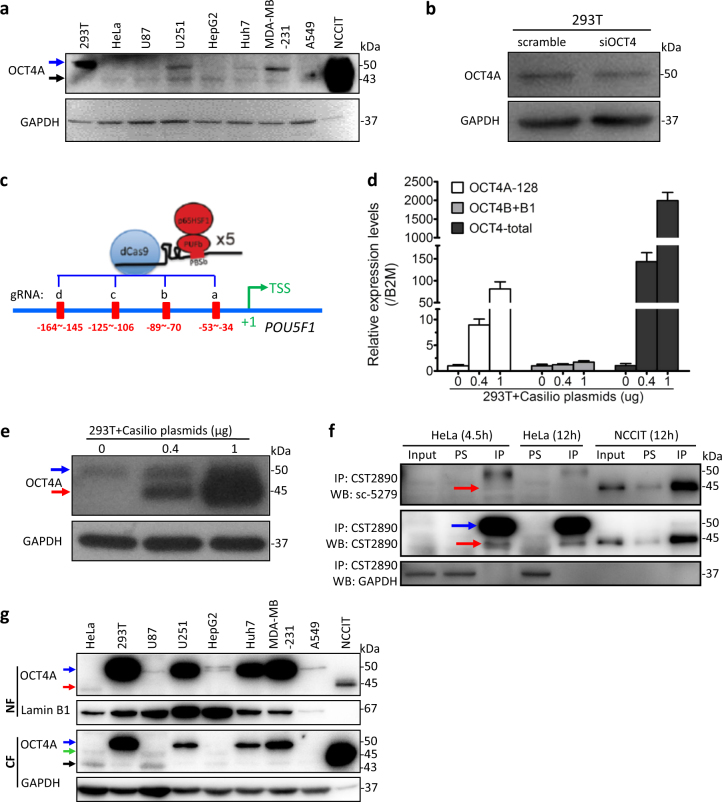
Detection of OCT4A proteins in somatic cancer cells via WB analysis with or without enrichment techniques. **a** WB analysis of whole cell lysates of the cell lines shown in Fig. 1c, the amount of NCCIT total protein loaded was only 1/50 of the other cell lines. **b** The whole cell lysates of 293T cells treated with scramble siRNA or siOCT4 were subjected to WB analysis. **c–e** A strategy to achieve transcriptional activation of the endogenous *POU5F1* using a dCas9-based activation system (Casilio) (**c**); qRT-PCR analysis using OCT4A-128, OCT4B + B1 and OCT4-total primers (**d**) and WB analysis (**e**) of 293T cells transfected with 0, 0.4, 1 μg Casilio plasmids, respectively. **f** HeLa and NCCIT whole cell lysates were IPed with CST2890 for indicated times and IP complexes were subjected to enhanced WB analysis with anti-OCT4A CST2890 or sc-5279, respectively. **g** Enhanced WB analysis of OCT4A protein in nuclear fraction (NF) and cytoplasmic fraction (CF) of the above cell lines. The NF of NCCIT was loaded with 1:10,000 dilution and the CF with 1:100 dilution compared with other undiluted samples. The blue arrows indicate the 50 kDa band, the red, green, and black arrows indicate the 45, 47, and 43 kDa band, respectively (the same below)

We then examined OCT4A protein in the above cells by regular western blotting (WB) analysis using a rabbit monoclonal antibody presumed to be OCT4A-specific (CST 2890). Similar to previous results from our own group^25,28,29^ and other groups^30–32^, we detected two major bands with apparent molecular size of 50 kDa (blue arrow, Fig. 2a) and 43 kDa (black arrow, Fig. 2a), respectively, in a variety of somatic cancer cell lines that differ from the 45 kDa band seen in NCCIT cells. We have previously demonstrated that in glioblastoma cell lines both the 47 kDa and 43 kDa bands recognized by the widely used mouse anti-OCT4A monoclonal antibody (Santa Cruz sc-5279) could be downregulated by an siRNA targeting all OCT4 isoforms and major *POU5F1* pseudogenes^25^. The 50 kDa band in 293T cells was also reduced by the same siRNA (Fig. 2b; Supplementary Figure 3A). We employed a dCas9-based gene activation system (termed Casilio^33^) (Fig. 2c) to activate endogenous OCT4A expression at both transcriptional (Fig. 2d) and translational levels. The intensity of a 45 kDa band (red arrow, Fig. 2e) increased proportionally as more Casilio plasmids were added while that of the 50 kDa band remained unchanged (blue arrow, Fig. 2e), suggesting that the 50 kDa band is not the authentic OCT4A protein and may represent a potential new product of *POU5F1*. Next, we extended the Casilio analyses to several somatic cancer cell lines and further confirmed that the apparent molecular weight of endogenous OCT4A protein in somatic cancer cells was identical to that in PSCs (Supplementary Figure 3B-E).

We therefore conducted immunoprecipitation (IP) to enrich OCT4A proteins and examined them with an enhanced WB method. Strikingly, a band with anticipated size (45 kDa) appeared in IP samples of HeLa cells when the above-mentioned CST 2890 was used for both IP and WB, which was less prominent when the sc-5279 was used (Fig. 2f). An attempt in identifying the 45 kDa band by MS analysis failed probably due to its extremely low abundance (data not shown). Moreover, the chief drawback of this IP-WB approach was the strong interfering signals from the 50 kDa rabbit IgG heavy chain band (blue arrow, Fig. 2f) which was often intermingled with the target 45 kDa band (red arrow, Fig. 2f). To enrich the OCT4A proteins that are presumably localized in cell nucleus, we then isolated the nuclear fraction (NF) from the cytoplasmic fraction (CF) in multiple cell lines and subjected both fractions to the above enhanced WB analysis. A clear but weak band of ~45 kDa was detected in the NF but not CF of HeLa cells (red arrow, Fig. 2g; Supplementary Figure 4A, B), which was weakly seen in the NF of some but not all cell lines (Supplementary Figure 4A, B). In contrast, the 50 kDa band (blue arrow, Fig. 2g) variably appeared in the NF of all the tested somatic cells with the exception of HeLa cell, while the 47 kDa (green arrow, Fig. 2g) and 43 kDa bands (black arrow, Fig. 2g) were detectable in some somatic cancer cells and mainly distributed in the cytoplasm (Fig. 2g).

Among all known OCT4-related proteins, OCT4-PG1 could interfere with OCT4A detection to the greatest extent due to its high amino acid sequence homology (>95%) and almost identical amino acid residue number (359 of OCT4-PG1 vs. 360 of OCT4A) with OCT4A^34,35^, its highly close protein band size to OCT4A^36^ and its nuclear localization^35,36^. In fact, Zhao et al.^36^ had already demonstrated that the widely used sc-5279 could recognize ectopically expressed OCT4-PG1. Unfortunately, the CST 2890 can also recognize OCT4-PG1 (Supplementary Figure 4C), further challenging the feasibility of detecting OCT4A protein in somatic cancer cells only by “OCT4A-specific” antibodies. Taken together, we obtained circumstantial evidence for the presence of the presumed 45 kDa OCT4A proteins in the nucleus of somatic cancer cells at an extremely low level, and also detected several more abundant OCT4-related proteins (with apparent molecular weight of 50, 47, and 43 kDa, respectively) in normal and cancerous somatic cells.

### Definitive identification and quantitation of authentic OCT4A proteins in somatic cancer cells

**Fig. 3:**
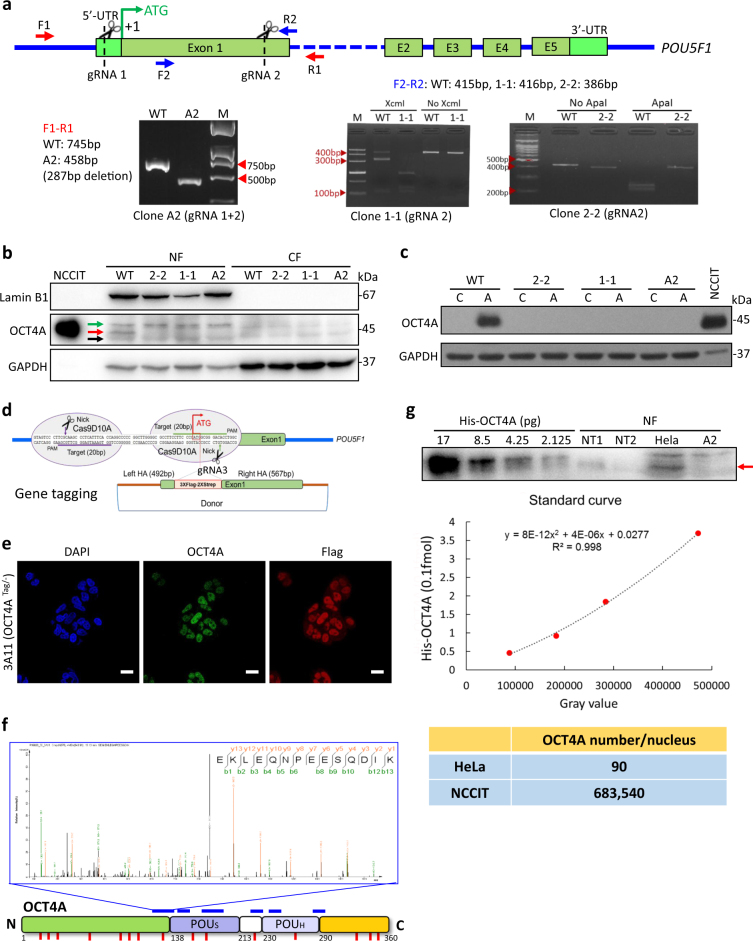
Identification and quantitation of endogenous authentic OCT4A proteins in somatic cancer cells. **a** Generation and verification of CRISPR/Cas9-mediated OCT4A-specific knockout (KO) cancer cell models. Upper panel: the strategy to obtain OCT4A-KO cancer cell clones. Note both single gRNA (gRNA2) and dual gRNAs (gRNA1 + gRNA2) were used to generate the OCT4A-KO HeLa cell clones. Lower panels: verification of homozygous OCT4A-KO clones by PCR (Left, A2 clone) or PCR-RFLP (Middle and Right, 1-1 and 2-2 clones). **b** Enhanced WB analysis of NF and CF of wild-type HeLa (WT) and OCT4A-KO HeLa clones (2-2, 1-1 and A2) with anti-OCT4A (CST 2890). **c** WT and OCT4A-KO clones subjected to Casilio activation followed by regular WB analysis. **d−f** Generation and characterization of CRISPR/Cas9-mediated knockin (KI) Tag-OCT4A HeLa cell clones. The strategy of gene KI to obtain Tag-OCT4A HeLa clones mediated by CRISPR/Cas9 and homologous recombination (**d**). Immunostaining of a single allele tagged Tag-OCT4A HeLa clone (3A11) with anti-OCT4A and anti-FLAG. Scale bars, 20 μm (**e**). IP and LC/MS/MS analysis of 3A11 HeLa clone for OCT4A identification (**f**). **g** Quantitation of endogenous OCT4A proteins in HeLa and NCCIT using enhanced WB analysis with recombinant His-OCT4A as a standard. Upper panel: enhanced WB analysis of His-OCT4A, NF of NCCIT (NT1: 1:20,000 dilution and NT2: 1:40,000 dilution) and HeLa cells, with NF of A2 clone as negative control. Middle panel: Standard curve of molar concentration and band intensity (gray value) for His-OCT4A. Lower panel: The calculated OCT4A protein molecules per cell nucleus based on absolute His-OCT4A protein concentrations determined by the above standard curve, and normalization by sample dilution factors and harvested cell numbers

To determine if the 45 kDa band truly corresponds to authentic OCT4A, we conducted specific OCT4A knockout in HeLa cells by taking the CRISPR-Cas9 approach. We succeeded in obtaining several carefully verified homozygous OCT4A-knockout (hereafter termed OCT4A-KO) clones (named 1-1, 2-2 and A2) (Fig. 3a; Supplementary Figures 5−7). Importantly, the PCR-direct sequencing showed that there was no off-target event occurred in the selected OCT4A-KO clones (Supplementary Table 1). Then, we observed that the nuclear-enriched 45 kDa band (red arrow, Fig. 3b) detected in WT completely disappeared in all OCT4A-KO clones whereas the nearby 47 kDa (green arrow, Fig. 3b) and 43 kDa bands (black arrow, Fig. 3b) remained largely unchanged. Furthermore, an anti-OCT4 antibody that recognizes all known OCT4 isoforms (Abcam ab109183) in the same WB analysis gave the same results (Supplementary Figure 8). In addition, the Casilio system that triggered a dramatic increase in the endogenous OCT4A protein level in WT did not induce any expression of the OCT4A protein in OCT4A-KO clones (Fig. 3c; Supplementary Figure 9A), and a parallel transfection experiment using a Cas9-GFP plasmid confirmed that the failure of Casilio-mediated expression of OCT4A proteins in OCT4A-KO clones was not due to the low transfection efficiency (Supplementary Figure 9B). Collectively, the nuclear 45 kDa band detected in HeLa cells most likely corresponded to the authentic OCT4A protein.

To obtain the amino acid sequence information for the endogenous 45 kDa protein whose level was too low to be applied to MS analysis, we knocked in (KI) a 3 × FLAG-2 × STREP tag with the Kozak sequence to the endogenous gene locus of *POU5F1* to generate an endogenously tagged OCT4A protein (termed Tag-OCT4A) (Fig. 3d). Since the Kozak sequence will presumably have no effect on gene transcription but can augment the translation of the Tag-OCT4A proteins^37^, such design can provide sufficient Tag-OCT4A proteins for subsequent IP-MS analysis yet still maintaining the endogenous features for gene transcription. We obtained a series of heterozygous KI clones, validated them by PCR-based genotyping with or without DNA sequencing (Supplementary Figure 10A−C). As expected, the Tag-OCT4A (50 kDa) could be detected readily by regular WB and IF (Fig. 3e; Supplementary Figures 10D, 11−12). In comparison, the signals of non-tagged endogenous OCT4A proteins (45 kDa) were still undetectable (Supplementary Figure 10D). IF analysis further confirmed the predominant nuclear localization of endogenous Tag-OCT4A in somatic cancer cells (Fig. 3e; Supplementary Figure 12). Among all heterozygotes with one allele inserted with tag sequence, the Tag-OCT4A protein levels varied considerably despite the same copy numbers (Supplementary Figures 10B, 10D and 12). Of note, the Tag-OCT4A protein levels even varied within the cell population derived from a single clone (Fig. 3e; Supplementary Figure 12). Collectively, these results revealed that OCT4A proteins were expressed heterogeneously in somatic cancer cells. We then selected the Tag-KI clone with the highest Tag-OCT4A protein level (3A11) for IP-MS analysis. Nine peptides that matched with six fragments of OCT4A protein and covered 16.94% of the full-length OCT4A protein sequence were successfully identified by MS (Fig. 3f; Supplementary Figures 13, 14). Importantly, one of the above identified peptide spanned the N-terminal domain (OCT4A specific) and POUs domain (common in OCT4A and OCT4B) of OCT4A and contained an amino acid that differs between OCT4A and OCT4-PG1, strongly suggesting that the identified peptide corresponded to authentic OCT4A protein (Fig. 3f). Besides, other two peptides were also found to carry an amino acid that differs from OCT4-PG1 (Fig. 3f; Supplementary Figure 14). Taken together, we concluded that authentic OCT4A proteins are present heterogeneously in somatic cancer cells.

To further quantify OCT4A protein levels in somatic cancer cells with relatively high accuracy, we adopted the NF-based enhanced WB approach to quantitate endogenous OCT4A proteins in HeLa and NCCIT cells with recombinant His-OCT4A as standard (Supplementary Figure 15A−C). On average, there were approximately 680,000 OCT4A protein molecules in each NCCIT nucleus vs. only 90 OCT4A protein molecules in each HeLa nucleus (Fig. 3g; Supplementary Figure 15D). Thus, we estimated that the OCT4A protein levels in somatic cancer cells are approximately 3−4 orders of magnitude lower than that in PSCs.

### OCT4A knockout altered the transcription of multiple cytoskeletal/adhesion molecules that converge on integrin and AP-1 signaling

**Fig. 4:**
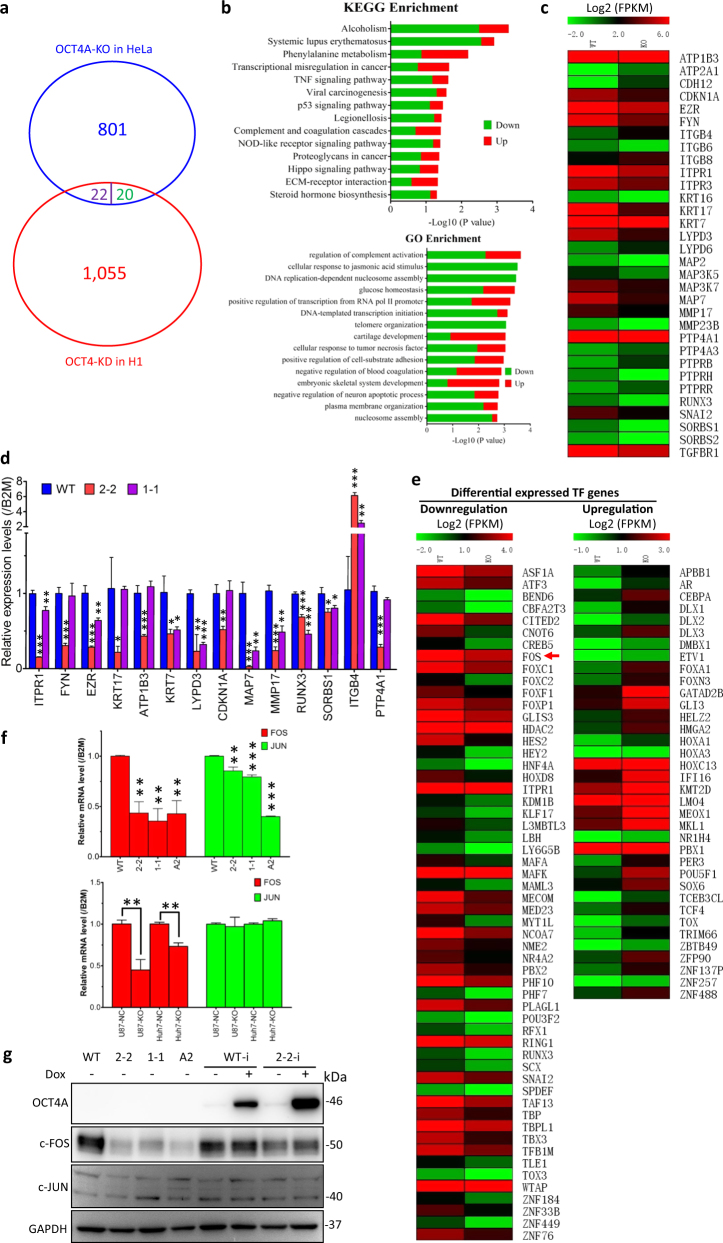
Alterations of the genome-wide transcriptome and multiple signaling pathways by OCT4A-KO in somatic cancer cells. **a** Venn diagram comparing the RNA-Seq differentially expressed genes (DEGs) between WT and OCT4A-KO (2-2) HeLa cells and the DNA microarray DEGs between OCT4 and siOCT4 in hESC H1 cell line reported by Babaie et al.^10^. Only 42 DEGs were shared by HeLa and H1 cells, of which 22 had opposite trend (purple), and 20 had similar trend (green). **b** KEGG (upper panel) and Gene Ontology (GO) (lower panel) analysis of the DEGs. **c** Heatmap of 32 selected DEGs involved in integrin-related pathways. **d** qRT-PCR analysis of the indicated transcripts in WT, 2-2, and 1-1 cells. **e** Heatmaps of 92 transcription factor (TF) genes identified by RNA-Seq analysis. Left panel: downregulated TF genes. Right panel: upregulated TF genes. **f** qRT-PCR analysis of FOS and JUN in WT and OCT4A-KO HeLa clones (upper panel) and population-based OCT4A-KO cancer cell models (U87/Huh7-NC/KO) (lower panel), respectively. **g** WB analysis of WT, OCT4A-KO HeLa clones, and HeLa cells inducibly expressed with ectopic OCT4A (WT-i and 2-2-i, with or without doxycycline (Dox) addition) with indicated antibodies. Uncropped original scans of the blots were shown in Supplementary Figure 23. The data in (**d**) and (**f**) shown were mean ± S.D. and mean ± S.E.M., respectively. The statistical significance was evaluated using the two-tailed unpaired Student’s *t* test. If not indicated, *P < 0.05, **P < 0.01 and ***P < 0.001 vs. “WT”

To search for genome-wide transcriptome alteration resulted from OCT4A knockout, we performed RNA-Seq analysis to compare differentially expressed genes (DEGs) between WT and OCT4A-KO clone 2-2. A total of 843 annotated human genes were upregulated (326 genes) or downregulated (517 genes) by more than twofold, respectively, in OCT4A-KO cells (Fig. 4a; Supplementary Table 7). We then compared our OCT4A-KO-based DEGs in HeLa with the published RNAi-mediated OCT4 knockdown-based DEGs in hESC cell line H1^10^. Overall, OCT4A ablation led to a global gene downregulation in HeLa cells (up vs. down: 326 vs. 517) but gene upregulation in H1 cells (up vs. down: 702 vs. 395), and only 42 DEGs were shared by HeLa and H1 cells, of which 22 had opposite trend (Fig. 4a and Supplementary Table 7). Further GO and KEGG analysis showed that the DEGs in OCT4A-KO HeLa cells were associated with multiple signaling pathways and transcriptional regulation (Fig. 4b). Thirty-two DEGs (up vs. down: 8 vs. 24) involved in integrin signaling pathway were selected and presented in the heatmap (Fig. 4c). Among them, 14 DEGs were further tested in two OCT4A-KO (2-2 and 1-1) clones by qRT-PCR (Fig. 4d). Compared to WT, all tested DEGs echoed the pattern of RNA-Seq data in 2-2 clone, and 9 out of the 14 DEGs that are largely related to cytoskeleton and cell adhesion also exhibited similar expression patterns in another OCT4A-KO clone 1-1 (Fig. 4d), suggesting that most DEGs were associated with OCT4A knockout per se.

It was reported that OCT4A regulates about 600 target genes in human ESCs where OCT4A, SOX2, and NANOG form a core transcriptional regulatory circuitry and their co-occupying genes encode a variety of important TFs^38^. We therefore screened TF genes from the 843 DEGs and identified a total of 92 TFs (up vs. down: 36 vs. 56) (Fig. 4e). Given the extremely low abundance of OCT4A in somatic cancer cells, we speculated that only a small portion of the 843 DEGs can be directly regulated by OCT4A and most DEGs may be indirectly controlled by OCT4A-regulated TFs. Therefore, by bioinformatics analyses, we searched for candidate TFs that could potentially bind to the enhancer and promoter regions of the above-mentioned 32 genes involved in adhesion-related pathways. Strikingly, c-FOS was predicted to bind to the enhancer/promoter regions of all the 32 genes (Supplementary Figure 16A). Among these 32 genes, *MMP17* and *CDKN1A* have been indicated as *FOS*/*AP-1* target genes in the literature^39,40^, and three other known *FOS*/*AP-1* target genes (*TERT*, *THBS1*, and *VEGFA*)^40,41^ were also listed in the 843 DEGs (Supplementary Figure 16B). Furthermore, both OCT4A and c-FOS were predicted to bind to the regulatory regions of multiple integrin signaling and adhesion-related genes (Supplementary Figure 16C). Indeed, c-FOS was one of the 56 downregulated TFs in OCT4A-KO clone 2-2, indicating its close correlation with OCT4A and the OCT4A-KO-mediated alteration of integrin signaling pathway in somatic cancer cells.

To verify that *FOS* is truly regulated by OCT4A in somatic cancer cells, we compared the *FOS* mRNA level between WT and OCT4A-KO clones (2-2, 1-1 and A2), between U87/Huh7-NC and U87/Huh7-KO (other somatic cancer cell models with CRISPR-Cas9-mediated non-targeting control (NC) and OCT4A-knockout (KO) in the whole cell population), respectively. All OCT4A-KO cells exhibited significantly reduced *FOS* mRNA levels, even though endogenous OCT4A protein in U87 and Huh7 cells is under detection limit of the current enhanced WB analysis, while *JUN* mRNA levels remained largely unchanged except for the A2 clone cells (Fig. 4f). Furthermore, WB analysis showed a dramatic reduction of c-FOS but not c-JUN protein levels in all OCT4A-KO HeLa clones (Fig. 4g). Intriguingly, ectopically introduced OCT4A, at its leaked expression level, partially but substantially increased c-FOS proteins in 2-2 clone cells (Fig. 4g, 2-2-i vs. 2-2), but the Dox-induced high-level expression of ectopic OCT4A did not further increase the c-FOS protein level in 2-2 cells (Fig. 4g, 2–2–i + Dox vs. 2-2-i). Collectively, OCT4A regulates *FOS* and other hundreds of genes (rather different from target genes in PSCs) involved in multiple signaling pathways (such as integrin signaling pathway) in somatic cancer cells.

### OCT4A directly and differentially regulated *FOS*/*AP-1* transcription at various protein levels

**Fig. 5:**
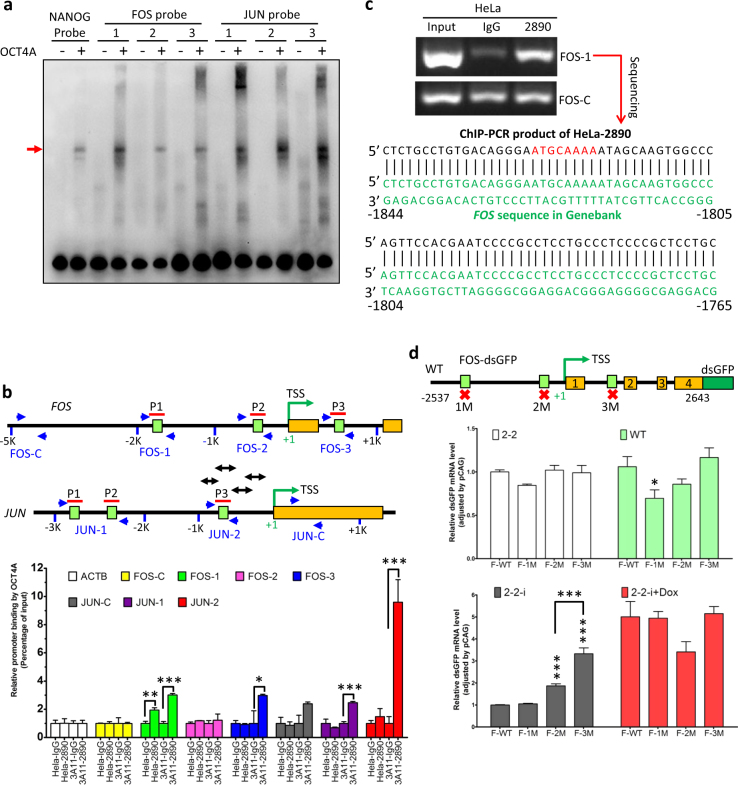
OCT4A was a transcription factor for *FOS*/*AP-1* in somatic cancer cells. **a** EMSA of three sets of biotinylated FOS and JUN probes (relative position shown in (**b**)), incubated respectively with recombinant His-OCT4A proteins. Biotinylated NANOG probe was applied as a positive control and the red arrow indicated the OCT4A-bound probes. **b** ChIP-qPCR analysis of WT and 3A11 HeLa cells with anti-OCT4A (CST 2890) and the indicated primers. Upper and middle panel: the schematic representation showing relative positions of predicted octamer motifs (the green boxes), probes (the red lines) and primers (the paired blue arrows) in human *FOS* (upper) and *JUN* (middle) genes. Double-headed arrows represent the four DNA fragments of promoter region of *JUN* covered by primers used by Kuo et al.^42^. Lower panel: ChIP-qPCR results quantified as relative promoter binding by OCT4A. **c** Anti-OCT4A (CST 2890)-based ChIP-PCR analysis of HeLa cells with the FOS-1 primers (upper panel). The ChIP-PCR products of HeLa-2890 were sequenced and the partial sequence (black) was aligned with *FOS* regulatory sequence in public database (green). The putative octamer motif is marked in red. **d** FOS-dsGFP reporter gene assay of cells with various OCT4A protein expression levels. Upper panel: Schematic representation of the components of normal or single octamer motif mutated FOS-dsGFP reporter plasmids. Middle and lower panels: 2-2, WT, 2-2-i and 2-2-i + Dox were transfected with the wild type or mutated FOS-dsGFP reporter plasmids (F-WT/1M/2M/3M) and the transcription of the reporter genes was evaluated by qRT-PCR. B2M was used as internal control and the relative dsGFP expression was normalized by pCAG primer. The data in (**b**) and (**d**) were expressed as mean ± S.D. and mean ± S.E.M., respectively. Two-tailed unpaired Student’s *t* tests were used. Unless specified, *P < 0.05, **P < 0.01 and ***P < 0.001 were vs. “F-WT”

*FOS* was not one of the identified target genes of OCT4A in human ESCs^38^, and a direct association between *FOS* and OCT4A has not been reported yet. Thus, our findings prompted us to explore whether OCT4A acts as a TF for *FOS*/*AP-1* gene in somatic cancer cells. We first performed an in silico search through the MatInspector tool in Genomatix platform for canonical octamer motif at the regulatory and coding regions of the *FOS* gene and its best-characterized partner *JUN* gene that was proved to be a direct target gene of OCT4A in stem-like liver cancer cells^42^ (Fig. 5b, middle panel, double-headed arrows). Three putative octamer motifs were identified in the *FOS* and *JUN* genes, respectively (Fig. 5b, upper and middle panel). We further confirmed that His-OCT4A proteins bound to all the six probes spanning the predicted octmaer motifs in vitro in EMSA, with the FOS probe 1 (spanning the motif numbered −1852~−1794 relative to the TSS) and the JUN probe 3 (spanning the motif numbered −694~−636 relative to the TSS) showing the highest binding affinity, respectively (Fig. 5a). Furthermore, we examined the OCT4A-*FOS*/*JUN* regulatory element interaction in vivo using ChIP assays. Firstly, ChIP-PCR analysis in HeLa cells revealed that the primers used were specific and the whole detection system was effective (Supplementary Figure 17). Secondly, ChIP-qPCR analysis was conducted with HeLa cells (containing extremely low level of endogenous OCT4A protein) and 3A11 clone cells (with translationally increased OCT4A protein level). For HeLa cells (WT), FOS-1 DNA fragments were significantly enriched in 2890 group compared to control IgG group while other *FOS* DNA fragments and all *JUN* DNA fragments were not enriched in 2890 group (Fig. 5b, bottom panel). For 3A11 cells (3A11), besides FOS-1 DNA fragments, both JUN-1 and JUN-2 DNA fragments were also substantially enriched in 2890 group compared to control IgG group (Fig. 5b, bottom panel). In comparison, neither the DNA fragments of *ACTB* gene nor those of non-octamer motif region in the *FOS* (–4983~−4808) and *JUN* (+406~+495) could be pulled down substantially by anti-OCT4A, indicating that the binding of OCT4A to the octamer motifs in the *FOS* and *JUN* promoter/enhancer was specific (Fig. 5b, bottom panel). Thirdly, DNA sequencing confirmed that the FOS-1 DNA fragments pulled down by the CST 2890 did contain the octamer motif shown in the above EMSA (Fig. 5c). In sum, we concluded that endogenous OCT4A in somatic cancer cells directly bound to the enhancer/promoter regions of the *FOS* gene.

To further evaluate the physiological relevance and significance of these three octamer motifs in *FOS* gene, we constructed FOS-dsGFP reporter plasmids harboring normal octamer motifs (wild type, F-WT) or one of the three octamer motif mutants (F-1M/2M/3M, respectively) (Fig. 5d) and introduced each of them into 2-2 (no OCT4A protein), WT (extremely low OCT4A), 2-2-i (leaked Flag-OCT4A), and 2-2-i + Dox (high level Flag-OCT4A), respectively. There was a slight decrease of F-1M-driven reporter transcription in 2-2 group that became more obvious in WT group but totally disappeared in 2-2-i and 2-2-i + Dox groups. In contrast, the F-2M-driven reporter transcription remained unchanged in 2-2 and WT, increased in 2-2-i but decreased in 2-2-i + Dox. The F-3M-driven reporter transcription only increased in 2-2-i group. Such complicated pattern indicated that depending on its level, OCT4A proteins may differentially bind to the three octamer motifs in *FOS* gene, either positively or negatively regulate its transcription. These data further confirmed the FOS-1, an octamer motif located in the putative distal enhancer region of *FOS* gene, plays a crucial role in *FOS* transcription when only trace amount of OCT4A is present.

Taken together, we demonstrated here that OCT4A is a TF for *FOS* and positively regulates its transcription via binding to the enhancer region of the *FOS* gene in somatic cancer cells.

### OCT4A knockout/FOS reduction was associated with migration defects and growth retardation of somatic cancer cells in vitro and in vivo

**Fig. 6:**
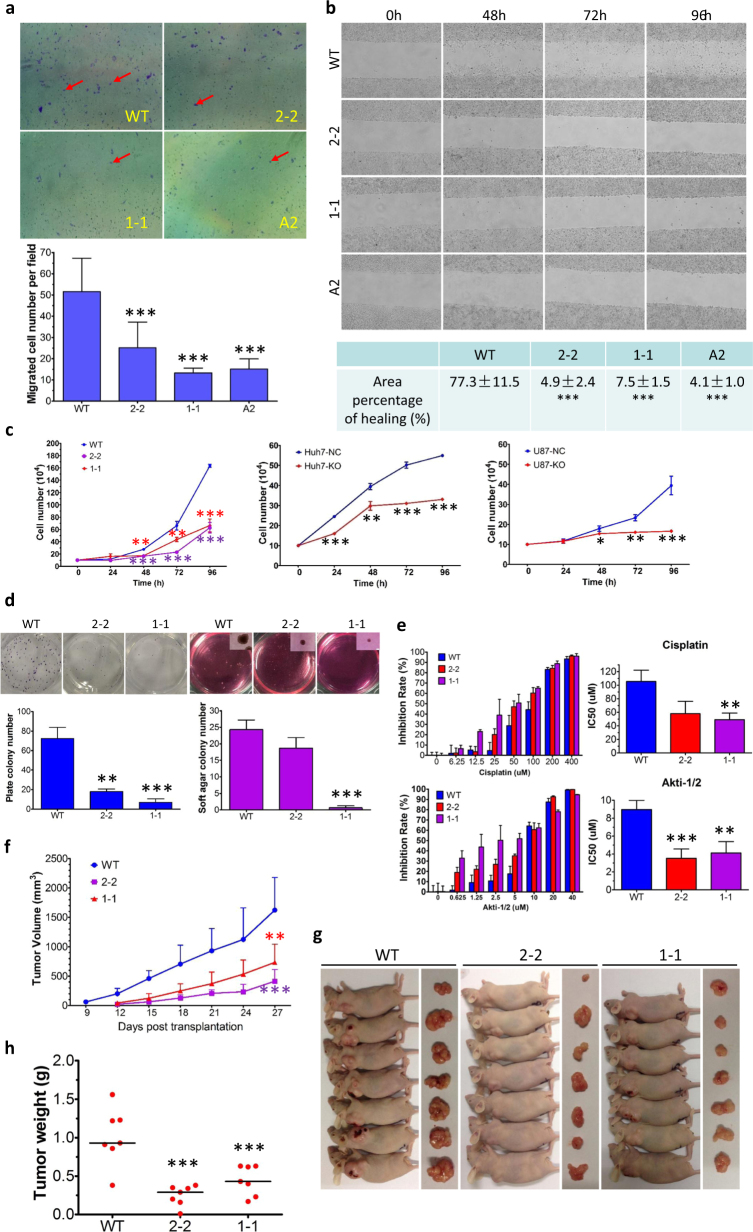
OCT4A knockout/FOS reduction was associated with migration defects and growth retardation of somatic cancer cells in vitro and in vivo. **a** Transwell migration assay of WT and OCT4A-KO clones. Upper panels: Representative images of migrated cells captured by microscope. Lower panel: quantification of migrated cells in upper panels. **b** Wound healing assay of WT and OCT4A-KO clones. Upper panels: Representative images of cells acquired by microscope at the indicated time points after the “wounds” were created. Lower panel: quantification of wound healing assays performed in upper panels. **c** Growth curves of WT and OCT4A-KO HeLa cells (Left panel), Huh7-NC and -KO cells (Middle panel) and U87-NC and -KO cells (Right panel). **d** Colony formation assays of WT and OCT4A-KO cells. Upper panels: representative photos of plate colony formation assay (Left) and soft agar colony formation assay with micrographs incorporated at the upper right corner (Right). Lower panels: quantifications of plate formation assays (Left) and soft agar colony formation assays (Right). **e** Chemosensitivity assay of WT and OCT4A-KO clones. Left panels: inhibition rate (IR) of Cisplatin (Upper) and Akti-1/2 (Lower) in WT and OCT4A-KO cells determined by CCK-8 analysis. IR data were expressed as mean ± S.D. Right panels: quantifications of IC_50_ values of Cisplatin (Upper) and Akti-1/2 (Lower) to WT and OCT4A-KO cells based on IRs in left panels. IC_50_ data were expressed as mean ± S.E.M. of three independent CCK-8 experiments. **f−h** Tumorigenicity analysis of WT and OCT4A-KO cells. Growth curves of xenograft tumors of WT and OCT4A-KO cells (**f**). Pictures of sacrificed mice bearing the tumors and the excised tumors (**g**). Comparison of tumor weights among WT and OCT4A-KO cells (**h**). Results shown in (**a**−**d**) were representative of three independent experiments. Two-tailed unpaired Student’s *t* tests were used for statistical analyses. *P < 0.05, **P < 0.01 and ***P < 0.001 were vs. “WT” or “NC”

To investigate the biological roles of endogenous OCT4A in somatic cancer cells, we first conducted wound healing assay and transwell migration assay to compare the cell migration capabilities of WT and OCT4A-KO clones, given that most validated DEGs in RNA-Seq were associated with cytoskeleton and cell adhesion. We found that the number of OCT4A-KO cells migrating through the transwell pores decreased remarkably compared with that of WT cells (Fig. 6a). Wound healing experiments demonstrated that the closure of the “wound” was completed within 96 h in WT cells but severely blocked in all the three OCT4A-KO clones (Fig. 6b), consistent with the result from the above transwell migration assay.

**Fig. 7:**
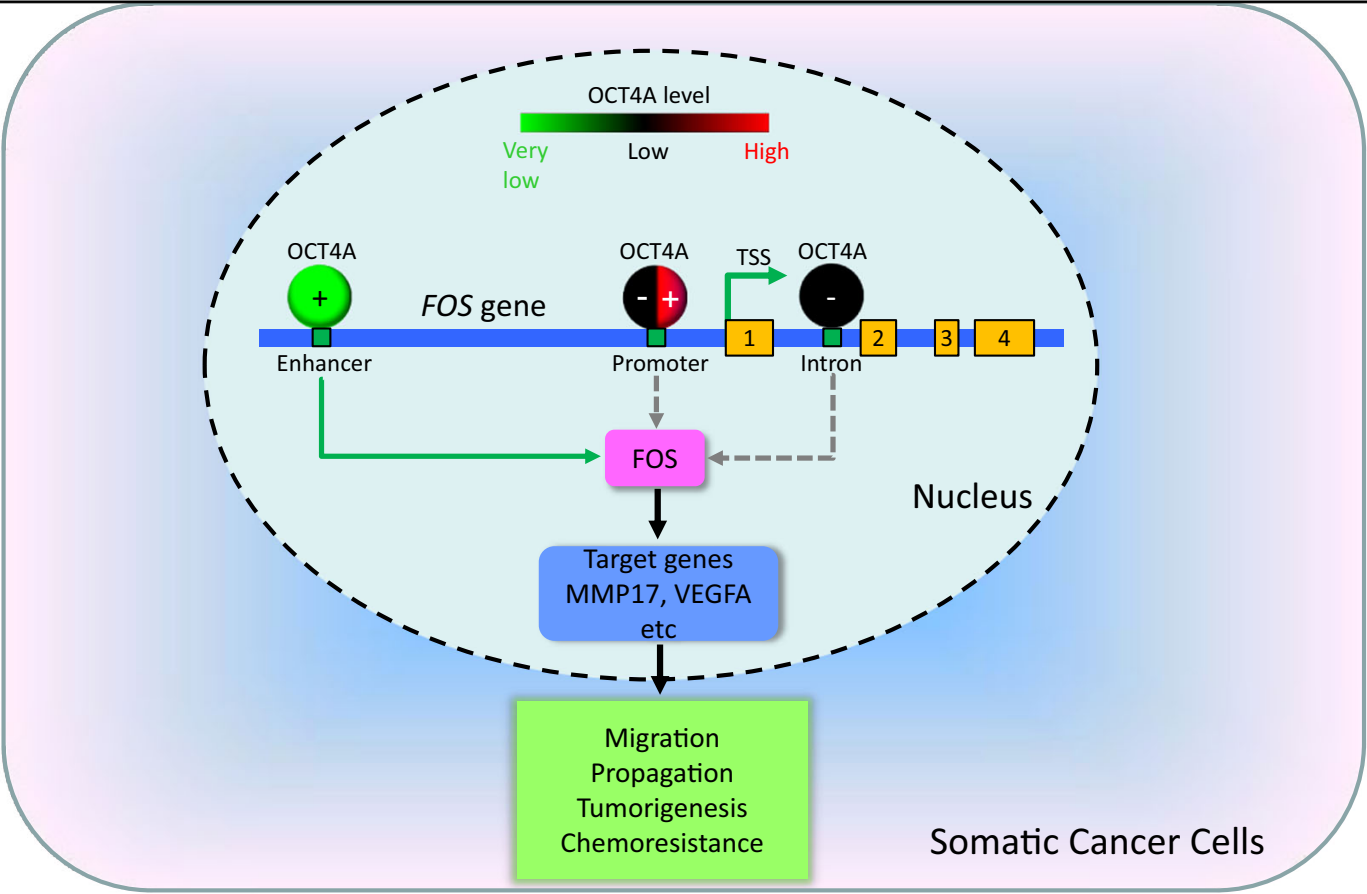
Working model of OCT4A in somatic cancer cells. In somatic cancer cells, authentic OCT4A proteins are heterogeneously expressed at very low levels in the cell nucleus. The trace amount of OCT4A proteins directly and preferentially bind to a distal enhancer region (green box, located at “Enhancer”) of *FOS* that has not been identified before, positively regulate the transcription of *FOS* and critically maintain its expression level, and thereby regulating numerous downstream genes (FOS target genes). The OCT4A-regulated genes in somatic cancer cells that are fundamentally distinct from those in PSCs play key roles in cell migration, propagation, tumorigenesis and chemoresistance, etc. There is an indication that at higher expression levels, OCT4A proteins may differentially bind to other octamer motifs on *FOS* (green boxes, located at “Promoter” and “Intron”), either inhibiting (“−”) or promoting (“ + ”) the transcription of *FOS*. The numbered yellow boxes stand for the four exons of *FOS* gene

Next, we evaluated the propagation of WT and OCT4A-KO clones by using direct cell counting-based growth assay. Both OCT4A-KO clones 2-2 and 1-1 showed significant growth retardation compared to WT cells (Fig. 6c, left panel). We extended this analysis to the above-mentioned OCT4A-KO cell population models of Huh7 and U87, which gave similar results (Fig. 6c, middle and right panels). Furthermore, the cell cycle analyses revealed that, compared with the WT cells, the OCT4A-KO cells had a decreased fraction of cells in G1 phase and an increased fraction of cells in G2/M phase and/or S phase (Supplementary Figure 18). Meanwhile, the flow cytometry-based apoptosis analyses showed increased proportions of OCT4A-KO cells that underwent apoptosis and necrosis (Supplementary Figure 19). Collectively, these findings indicated that OCT4A-KO led to growth retardation by simultaneously inducing cell cycle arrest and apoptotic cell death.

We then attempted to find out if the above OCT4A-KO phenotypes could be rescued by c-FOS overexpression. The OCT4A-KO clone 2-2 cells were transiently transfected with a plasmid comprising c-FOS + dsGFP driven by *FOS* promoter (designated as pFOS-GFP) or a plasmid with only dsGFP driven by *FOS* promoter (designated as pGFP) as a control. Quantification of the mRNA levels of *FOS* and the documented *FOS* target genes (*TERT* and *CDKN1A*) was used to assess the efficiency of pFOS-GFP overexpression (Supplementary Figure 20A). Remarkably, the propagation and migration capabilities of 2-2 were partially rescued by the pFOS-GFP but not the pGFP plasmid (Supplementary Figure 20B, C), implicating the involvement of c-FOS in OCT4A-controlled phenotypes.

We then asked if OCT4A plays a definitive role in anticancer drug resistance, as multiple studies have indicated its involvement in promoting cellular resistance to chemotherapy based on RNAi strategies that were not specifically targeting OCT4A^25,29,43,44^. We treated the WT and OCT4A-KO cells with Cisplatin, a first-line anticancer drug for cervical carcinoma, or Akti-1/2, a potent anticancer agent in clinical trial. Compared to the WT cells, the OCT4A-KO cells exhibited higher sensitivity to both Cisplatin and Akti-1/2, although the difference of IC_50_ values of Cisplatin between WT and 2-2 did not reach statistical significance (Fig. 6e). Remarkably, 2-2 cells exhibited a much higher apoptosis and necrosis rate than WT cells when treated with Cisplatin and analyzed by flow cytometry (Supplementary Figure 19).

The plate colony formation assay revealed that OCT4A-KO dramatically reduced the proportion of cells with colony formation ability (self-renewal potential, a critical feature of CSCs/TICs) (Fig. 6d, left panels). Similarly, the soft agar colony formation assay demonstrated that OCT4A-KO also led to an impaired foci formation capacity, with the 1-1 clone exhibiting an extremely low colony-forming efficiency. Although the colony-forming efficiency of the 2-2 clone was only reduced by 20%, the average size of 2-2 colonies was much smaller than that of WT, also indicating a strong decay in colony-forming capacity (Fig. 6d, right panels). Furthermore, the tumorsphere formation assay showed that only WT cells can generate a few tumorspheres at an initial plating number of 100 cells/well, and by contrast, OCT4A-KO cells failed to form any tumorspheres in such condition, suggesting substantial shrinkage of CSC/TIC pool (Supplementary Figure 21).

To extend the above analyses to an in vivo setting, WT and OCT4A-KO cells (2-2 and 1-1) were inoculated subcutaneously into nude mice. WT cells formed visible xenograft tumors in most inoculated mice at the ninth day after transplantation, whereas the OCT4A-KO cell-derived tumors were not visible until the twelfth day (Fig. 6f). Moreover, the xenograft tumor expansion rate of the WT group was always remarkably higher than that of both OCT4A-KO groups (Fig. 6f). Last, the tumor weight of the WT group was significantly higher than that of both OCT4A-KO groups (Fig. 6g, h). To sum up, OCT4A-KO led to migration defects, dampened self-renewal capacities and growth retardation of somatic cancer cells in vitro and in vivo.

## Discussion

By a combination of state-of-the-art approaches, we resolve the long-standing controversy and provide unequivocal evidence here that full-length authentic OCT4A transcripts and proteins are present in somatic cancer cells. Despite numerous reports claiming OCT4A is present and upregulated in somatic cancer cells/tissues and CSCs, few are solid enough to be countable. In fact, due to intrinsic detection specificity problems associated with the PCR primers and OCT4A antibodies employed and intrinsic limitations of using siRNA/shRNA in functionally validating the presence of OCT4A, we believe that the data or interpretations regarding OCT4A detection in somatic cancer cells in many published reports were misleading, as pointed out previously^7,22^. Here, we showed that the full-length authentic OCT4A transcripts were detected only in somatic cancer cell lines but not in 293T, LO2 and normal liver cells while the short OCT4A primer pairs can give positive signals for all tested cancerous and non-cancerous cells. Since the detection of short fragments of OCT4A cannot guarantee the existence of its full-length transcripts due to gDNA contamination, alternative splicing etc., our finding underscores the importance of amplifying the specific full-length transcripts of OCT4A instead of its small portions when trying to make a claim that authentic OCT4A transcripts are present in somatic cells. Furthermore, in many studies, the protein samples for somatic cells and PSCs were not run on the same gel and therefore the relative positions of the OCT4 bands on immunoblots cannot be meaningfully compared^24,27^. Our present data showed that the apparent molecular weight of the authentic OCT4A band in somatic cancer cells is approximately 45 kDa, identical or very close to that of the OCT4A in PSCs. In contrast, the commonly presumed OCT4 proteins in the literature probably correspond to either the 50 kDa, 47 kDa or 43 kDa band, etc. shown in this study. Since these OCT4 bands can be recognized by commercially available antibodies, and can be reduced by siRNAs/shRNAs targeting *POU5F1* and several of its pseudogenes but not by OCT4A-specific gRNA-Cas9, they are likely to be protein products from OCT4 pseudogenes, potential new isoforms generated by alternative splicing, or even other POU family members. Future work is needed to uncover the identity of those bands, their potential connections with OCT4A and potential roles in somatic cancer cells.

In this study, we showed that endogenous OCT4A protein is predominantly localized in the nucleus in somatic cancer cells, consistent with its putative functions as a TF in PSCs^38^. Furthermore, for the first time, we quantified the levels of OCT4A transcripts and proteins in somatic cancer cells. On average, there are approximately 90 OCT4A protein molecules in each HeLa cells, a level that is ~7500-fold lower than that in pluripotent NCCIT cells. Considering the heterogeneity of the OCT4A protein levels in HeLa cell population and approximately 10% of the total HeLa cells contain relatively high levels of OCT4A that may represent the CSCs, even the OCT4A level in CSCs is ~750-fold lower than that in PSCs. Such low levels of endogenous OCT4A protein present in somatic cancer cells explain the difficulty of its detection by routine methods. Our findings have at least four implications: first, for somatic cancer cells or CSCs, extreme caution should be exercised when interpreting the OCT4 bands on immunoblots, the bands recognized by anti-OCT4A antibodies should be further validated by more stringent means such as OCT4A-specific gRNA-Cas9-mediated knockout. Second, highly sensitive detection techniques are crucial for detecting proteins with extremely low abundance. In this study, we combined the subcellular fraction with signal enhancement methods to improve detection sensitivity of regular WB. The detection limit of this NF-based enhanced WB approach is about 1 pg OCT4A, merely picking up the endogenous OCT4A level (about 3.8 pg) in bulk HeLa cells that was not detectable by routine WB^45^. However, it is still not sensitive enough to pick up the endogenous OCT4A protein band in most other somatic cancer cells that express the full-length OCT4A transcripts. Third, the poor correlation between OCT4A transcript level and its protein level among the analyzed somatic cancer cell lines strongly suggested that OCT4A expression is under control at multiple layers (transcriptional, post-transcriptional, especially translational and post-translational level, etc.) in somatic cancer cells. Fourth, proteins with extremely low abundance (even below the detection limit of routine methods) may still exert essential biological functions.

Remarkably, we revealed that despite its low abundance in somatic cancer cells, endogenous OCT4A proteins bind to the promoter/enhancer regions of the *FOS*/*AP-1* gene and critically regulate its transcription (Fig. 7). OCT4A proteins in PSCs are known to bind to and regulate hundreds of target genes mainly associated with pluripotency maintenance and lineage specification^10,38,46^. In very small embryonic-like mesenchymal stem cells, OCT4 and HIF-2α jointly regulate cell survival genes including Bcl2 and Survivin^47^. In contrast, much less is known about the OCT4 target genes in somatic cancer cells. By using ChIP sequencing, Tang et al.^48^ identified thousands of genomic regions that are potential OCT4-binding regions in the OCT4A-overexpressing lung cancer cell line A549. The candidate OCT4 target genes in A549 cells minimally overlapped with OCT4A target genes in PSCs. However, the main caveat of their study is that overexpressed OCT4A could bind to target genes that are not physiologically bound by the extremely low quantity of endogenous OCT4A proteins present in somatic cancer cells. Here we provide compelling evidence using both cancer cells with non-edited *POU5F1* gene and cancer cells whose *POU5F1* gene locus was inserted with a Tag sequence, endogenous OCT4A proteins preferentially bound to the −1826~−1819 bp region of the *FOS* promoter/enhancer in somatic cancer cells. Moreover, transient overexpression of c-FOS could partially rescue some of the OCT4A KO phenotypes including declined migration capability and growth inhibition.

Taken together, we overcome several major pitfalls associated with OCT4A detection and provide unequivocal evidence here that both full-length authentic OCT4A transcripts and proteins are present at low and varied levels in somatic cancer cells. These OCT4A proteins critically control the transcription of *FOS*/*AP-1*, and thereby regulating the adhesion, metastasis and propagation of somatic cancer cells. Targeting OCT4A in a combination therapy may hold great promise in combating human cancers.

## Materials and methods

### Regular/enhanced western blotting

Whole cell lysate preparation and regular WB were performed as previously described^28,49^. Preparation of NF and CF was conducted using a protein extraction kit (Beyotime P0027) according to the manufacturer’s instruction. For enhanced WB analysis, the SuperSignal Western Blot Enhancer kit (Thermo 46640) was employed to enhance detection sensitivity.

### Recombinant protein expression, purification, and endogenous OCT4A quantification in cancer cells

The prokaryotic expression and purification of His-tagged OCT4A (His-OCT4A) recombinant proteins was conducted as previously described^50^. The enhanced WB-based quantification of endogenous OCT4A protein was carried out as described in the Supplementary Materials and Methods.

### Transcriptional activation of endogenous *POU5F1* gene

Endogenous *POU5F1* gene transcription activation was performed according to the original report^33^. In brief, each cell line was seeded at 100,000 cells per well in a 12-well plate 16 h before transfection. One microgram Casilio plasmids were lipo-transfected and harvested at 72 h after transfection for qRT-PCR and WB analysis.

### Mouse xenograft tumor models

The animal experiments were carried out under the Guide for the Care and Use of Animals for Research Purposes and have been approved by the Committee of the Ethics of Animal Experiments of the Zhejiang University. The source of BALB/c nude mice, the breeding condition and the operation criteria were described previously^28,29^. WT and OCT4A-KO (2-2 and 1-1) HeLa cells, 1×10^6^ each, were inoculated subcutaneously into each mouse, respectively. Once the xenograft tumors were visible and touchable, the volume of the tumors was evaluated.

### Statistical analyses

All quantitative results are expressed as mean values ± S.D. or ±S.E.M. All statistical analyses were conducted using the GraphPad Prism 5.0 statistics software released by GraphPad Software, Inc. The statistical significance was evaluated using the two-tailed unpaired Student’s *t* test, and differences were considered significant at *P < 0.05, **P < 0.01, and ***P < 0.001.

## Electronic supplementary material

Supplementary figures

Supplemental methods

Supplementary Table 7
