# Supplementary material for: Endogenous authentic OCT4A proteins directly regulate FOS/AP-1 transcription in somatic cancer cells
Source: Cell Death Dis. 2018 May 22;9(6):585. doi: 10.1038/s41419-018-0606-x (PMC5964179; doi:10.1038/s41419-018-0606-x)
Supplement: Supplementary file 1 — Supplementary figures [file 41419_2018_606_MOESM1_ESM.ppt]

## Slide 1
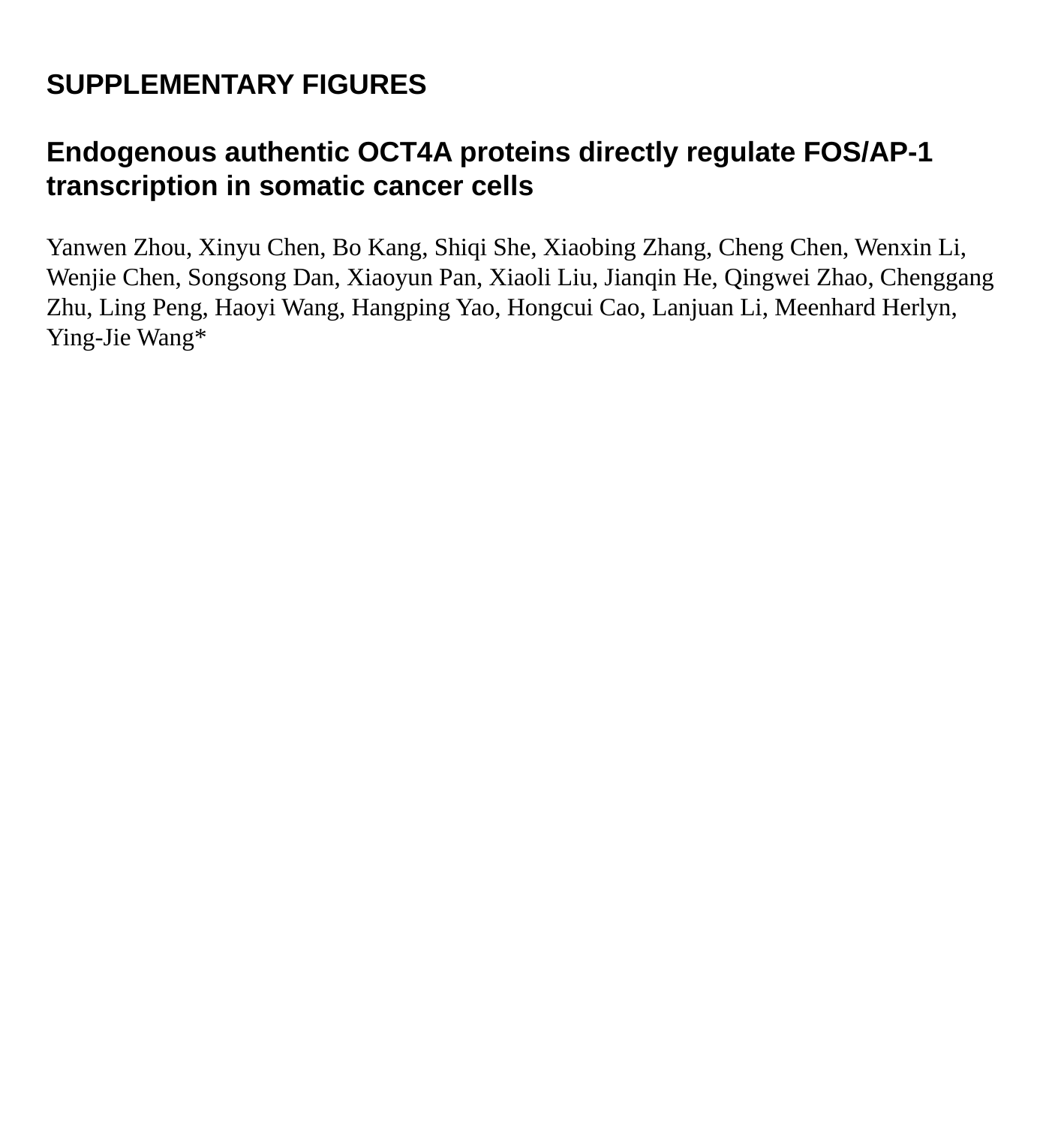

SUPPLEMENTARY FIGURES
Endogenous authentic OCT4A proteins directly regulate FOS/AP-1 transcription in somatic cancer cells
Yanwen Zhou, Xinyu Chen, Bo Kang, Shiqi She, Xiaobing Zhang, Cheng Chen, Wenxin Li, Wenjie Chen, Songsong Dan, Xiaoyun Pan, Xiaoli Liu, Jianqin He, Qingwei Zhao, Chenggang Zhu, Ling Peng, Haoyi Wang, Hangping Yao, Hongcui Cao, Lanjuan Li, Meenhard Herlyn, Ying-Jie Wang*

## Slide 2
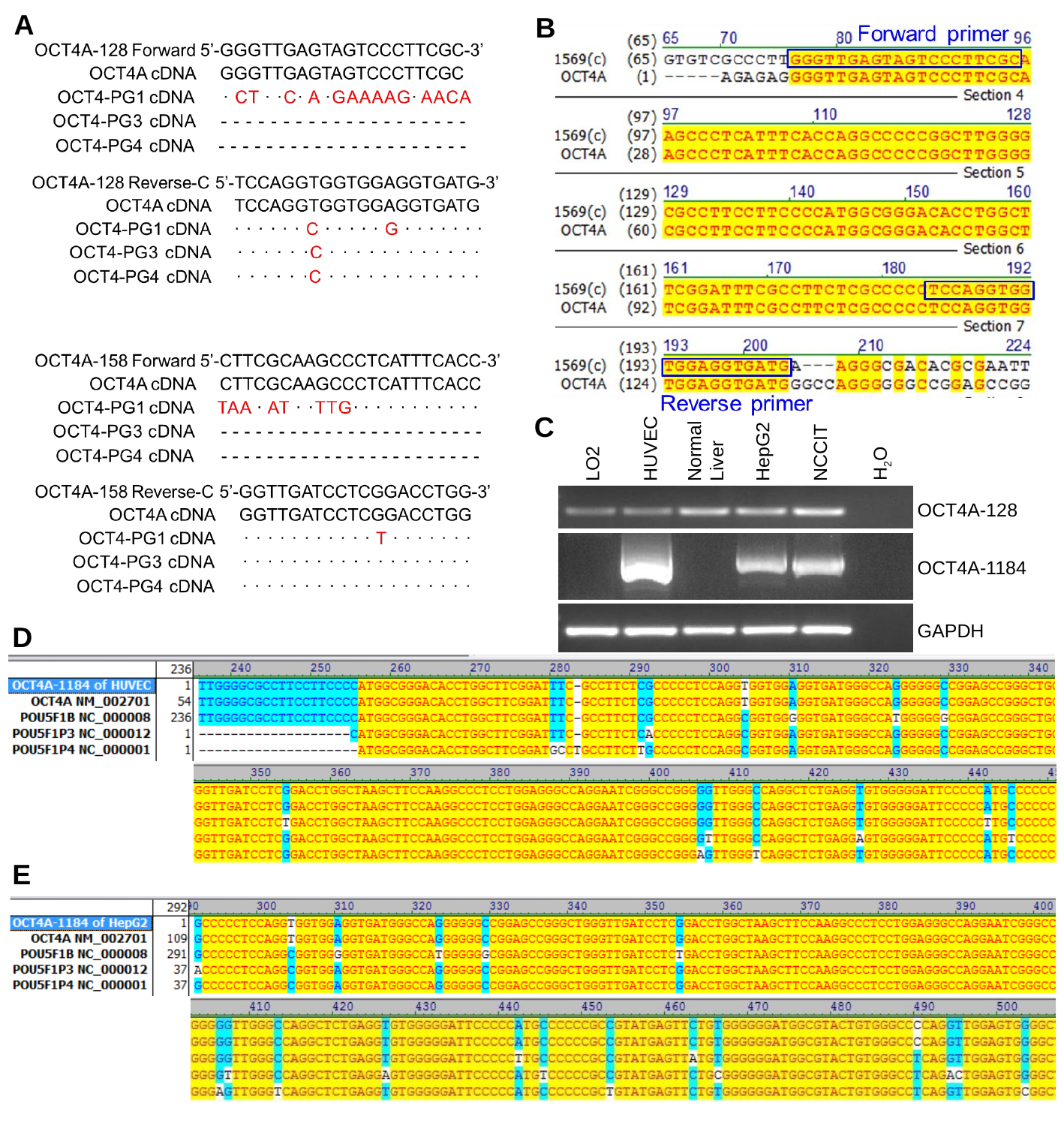

A
B
C
Normal
Liver
HUVEC
HepG2
NCCIT
H2O
LO2
OCT4A-1184
OCT4A-128
GAPDH
D
E

## Slide 3
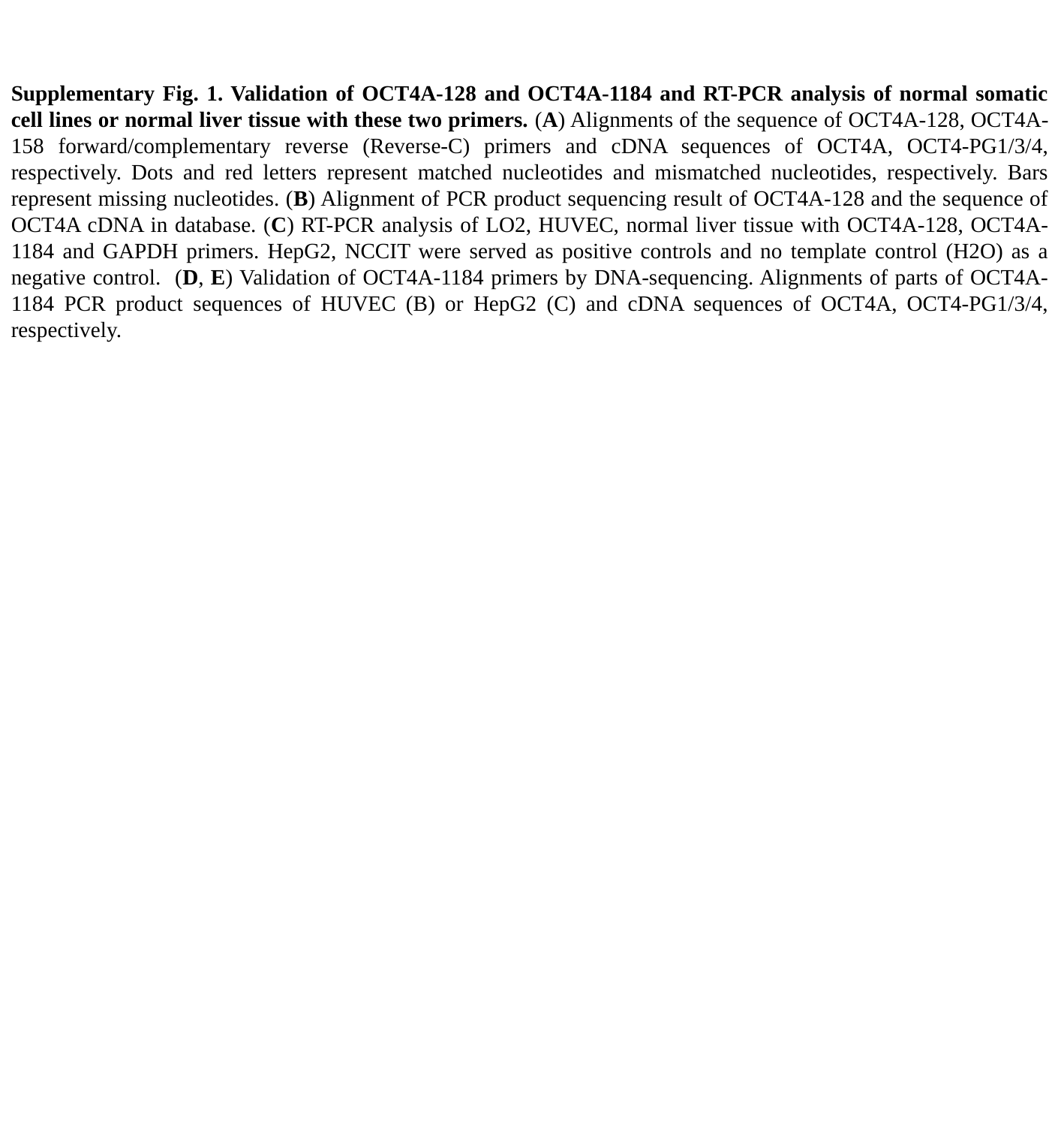

Supplementary Fig. 1. Validation of OCT4A-128 and OCT4A-1184 and RT-PCR analysis of normal somatic cell lines or normal liver tissue with these two primers. (A) Alignments of the sequence of OCT4A-128, OCT4A-158 forward/complementary reverse (Reverse-C) primers and cDNA sequences of OCT4A, OCT4-PG1/3/4, respectively. Dots and red letters represent matched nucleotides and mismatched nucleotides, respectively. Bars represent missing nucleotides. (B) Alignment of PCR product sequencing result of OCT4A-128 and the sequence of OCT4A cDNA in database. (C) RT-PCR analysis of LO2, HUVEC, normal liver tissue with OCT4A-128, OCT4A-1184 and GAPDH primers. HepG2, NCCIT were served as positive controls and no template control (H2O) as a negative control. (D, E) Validation of OCT4A-1184 primers by DNA-sequencing. Alignments of parts of OCT4A-1184 PCR product sequences of HUVEC (B) or HepG2 (C) and cDNA sequences of OCT4A, OCT4-PG1/3/4, respectively.

## Slide 4
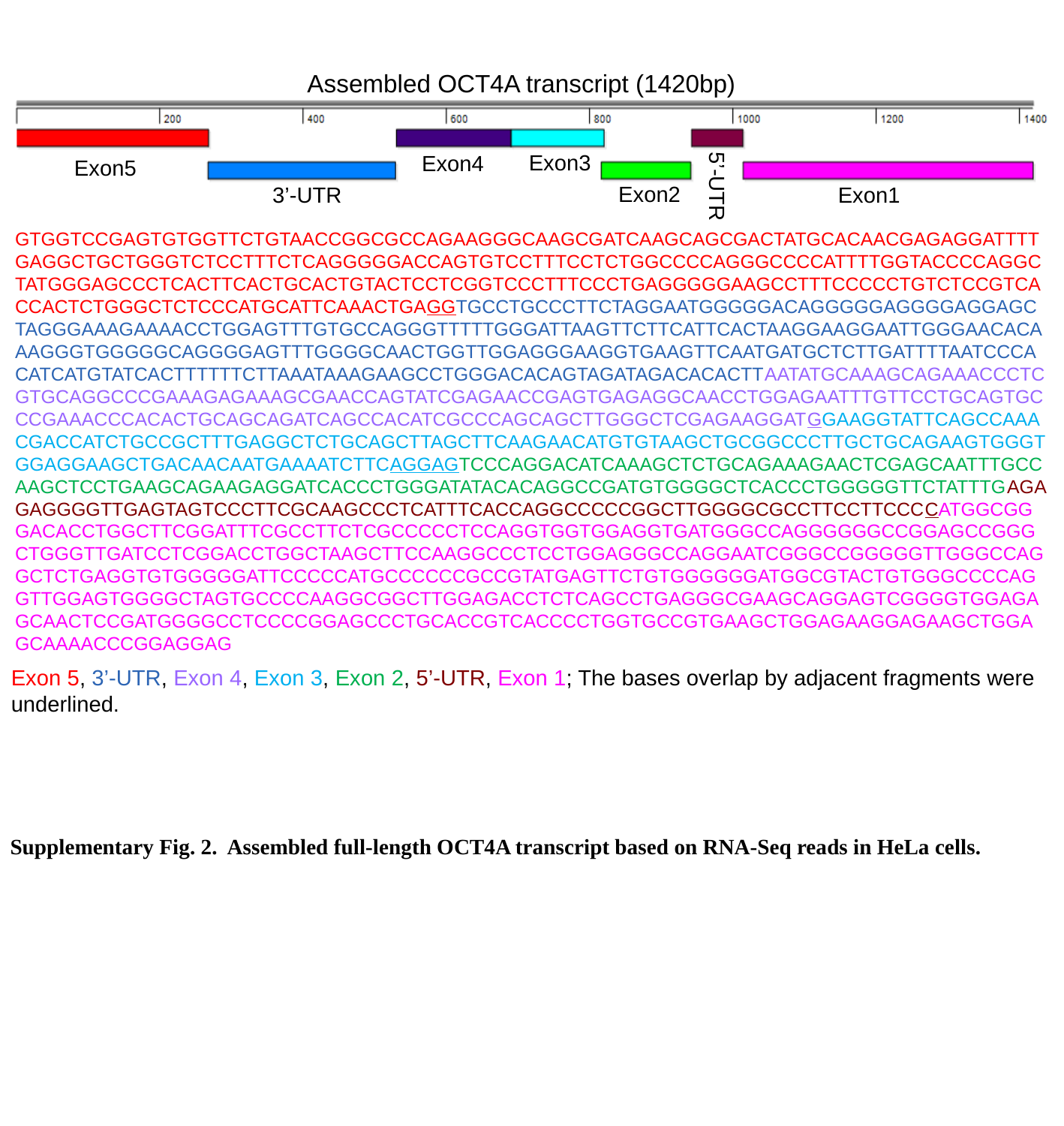

Assembled OCT4A transcript (1420bp)
Exon3
Exon4
Exon5
5’-UTR
Exon2
3’-UTR
Exon1
GTGGTCCGAGTGTGGTTCTGTAACCGGCGCCAGAAGGGCAAGCGATCAAGCAGCGACTATGCACAACGAGAGGATTTTGAGGCTGCTGGGTCTCCTTTCTCAGGGGGACCAGTGTCCTTTCCTCTGGCCCCAGGGCCCCATTTTGGTACCCCAGGCTATGGGAGCCCTCACTTCACTGCACTGTACTCCTCGGTCCCTTTCCCTGAGGGGGAAGCCTTTCCCCCTGTCTCCGTCACCACTCTGGGCTCTCCCATGCATTCAAACTGAGGTGCCTGCCCTTCTAGGAATGGGGGACAGGGGGAGGGGAGGAGCTAGGGAAAGAAAACCTGGAGTTTGTGCCAGGGTTTTTGGGATTAAGTTCTTCATTCACTAAGGAAGGAATTGGGAACACAAAGGGTGGGGGCAGGGGAGTTTGGGGCAACTGGTTGGAGGGAAGGTGAAGTTCAATGATGCTCTTGATTTTAATCCCACATCATGTATCACTTTTTTCTTAAATAAAGAAGCCTGGGACACAGTAGATAGACACACTTAATATGCAAAGCAGAAACCCTCGTGCAGGCCCGAAAGAGAAAGCGAACCAGTATCGAGAACCGAGTGAGAGGCAACCTGGAGAATTTGTTCCTGCAGTGCCCGAAACCCACACTGCAGCAGATCAGCCACATCGCCCAGCAGCTTGGGCTCGAGAAGGATGGAAGGTATTCAGCCAAACGACCATCTGCCGCTTTGAGGCTCTGCAGCTTAGCTTCAAGAACATGTGTAAGCTGCGGCCCTTGCTGCAGAAGTGGGTGGAGGAAGCTGACAACAATGAAAATCTTCAGGAGTCCCAGGACATCAAAGCTCTGCAGAAAGAACTCGAGCAATTTGCCAAGCTCCTGAAGCAGAAGAGGATCACCCTGGGATATACACAGGCCGATGTGGGGCTCACCCTGGGGGTTCTATTTGAGAGAGGGGTTGAGTAGTCCCTTCGCAAGCCCTCATTTCACCAGGCCCCCGGCTTGGGGCGCCTTCCTTCCCCATGGCGGGACACCTGGCTTCGGATTTCGCCTTCTCGCCCCCTCCAGGTGGTGGAGGTGATGGGCCAGGGGGGCCGGAGCCGGGCTGGGTTGATCCTCGGACCTGGCTAAGCTTCCAAGGCCCTCCTGGAGGGCCAGGAATCGGGCCGGGGGTTGGGCCAGGCTCTGAGGTGTGGGGGATTCCCCCATGCCCCCCGCCGTATGAGTTCTGTGGGGGGATGGCGTACTGTGGGCCCCAGGTTGGAGTGGGGCTAGTGCCCCAAGGCGGCTTGGAGACCTCTCAGCCTGAGGGCGAAGCAGGAGTCGGGGTGGAGAGCAACTCCGATGGGGCCTCCCCGGAGCCCTGCACCGTCACCCCTGGTGCCGTGAAGCTGGAGAAGGAGAAGCTGGAGCAAAACCCGGAGGAG
Exon 5, 3’-UTR, Exon 4, Exon 3, Exon 2, 5’-UTR, Exon 1; The bases overlap by adjacent fragments were underlined.
Supplementary Fig. 2. Assembled full-length OCT4A transcript based on RNA-Seq reads in HeLa cells.

## Slide 5
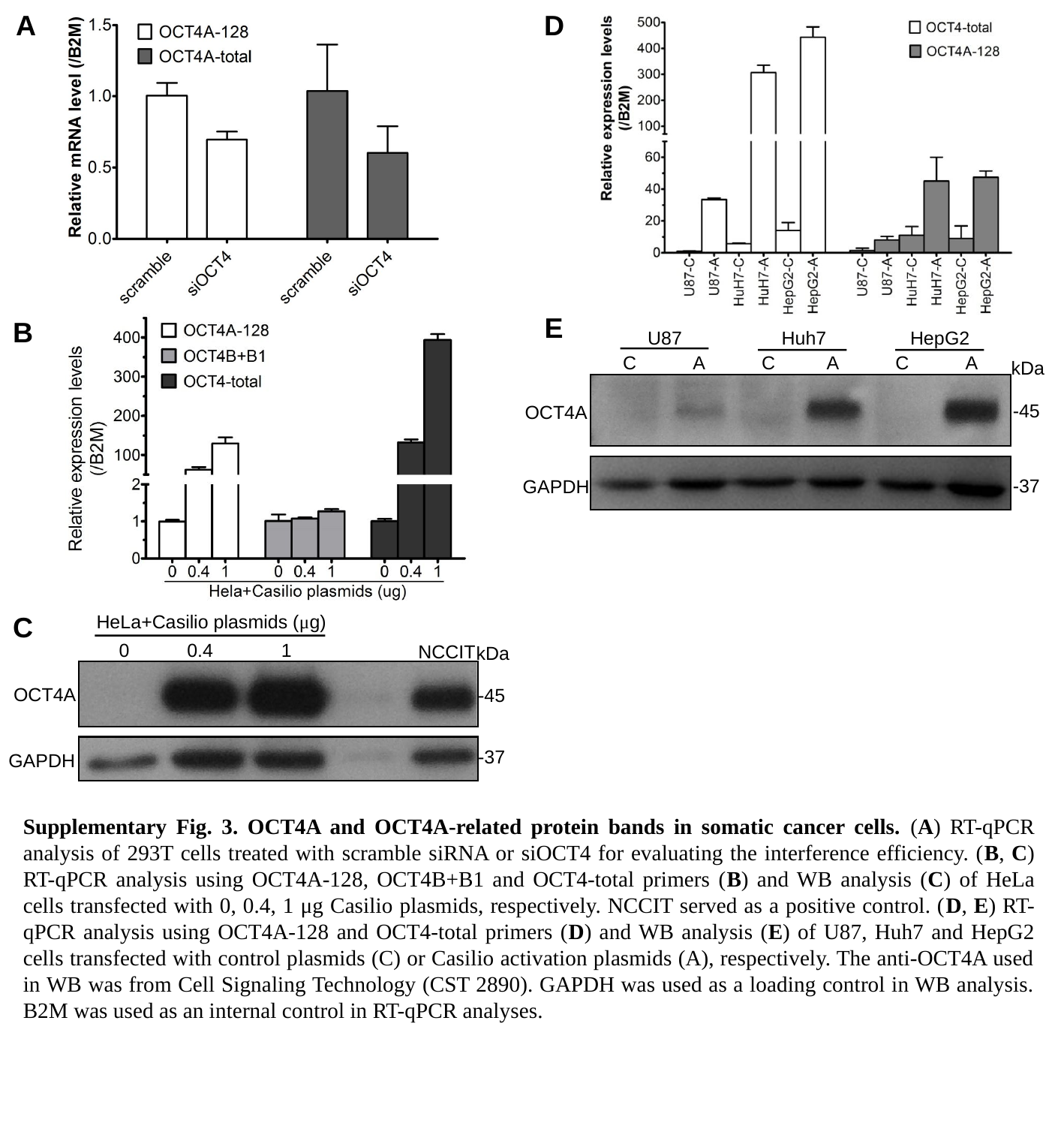

A
D
E
B
U87 Huh7 HepG2
C A C A C A
OCT4A
GAPDH
kDa
-45
-37
C
HeLa+Casilio plasmids (μg)
0 0.4 1
NCCIT
OCT4A
GAPDH
kDa
-45
-37
Supplementary Fig. 3. OCT4A and OCT4A-related protein bands in somatic cancer cells. (A) RT-qPCR analysis of 293T cells treated with scramble siRNA or siOCT4 for evaluating the interference efficiency. (B, C) RT-qPCR analysis using OCT4A-128, OCT4B+B1 and OCT4-total primers (B) and WB analysis (C) of HeLa cells transfected with 0, 0.4, 1 μg Casilio plasmids, respectively. NCCIT served as a positive control. (D, E) RT-qPCR analysis using OCT4A-128 and OCT4-total primers (D) and WB analysis (E) of U87, Huh7 and HepG2 cells transfected with control plasmids (C) or Casilio activation plasmids (A), respectively. The anti-OCT4A used in WB was from Cell Signaling Technology (CST 2890). GAPDH was used as a loading control in WB analysis. B2M was used as an internal control in RT-qPCR analyses.

## Slide 6
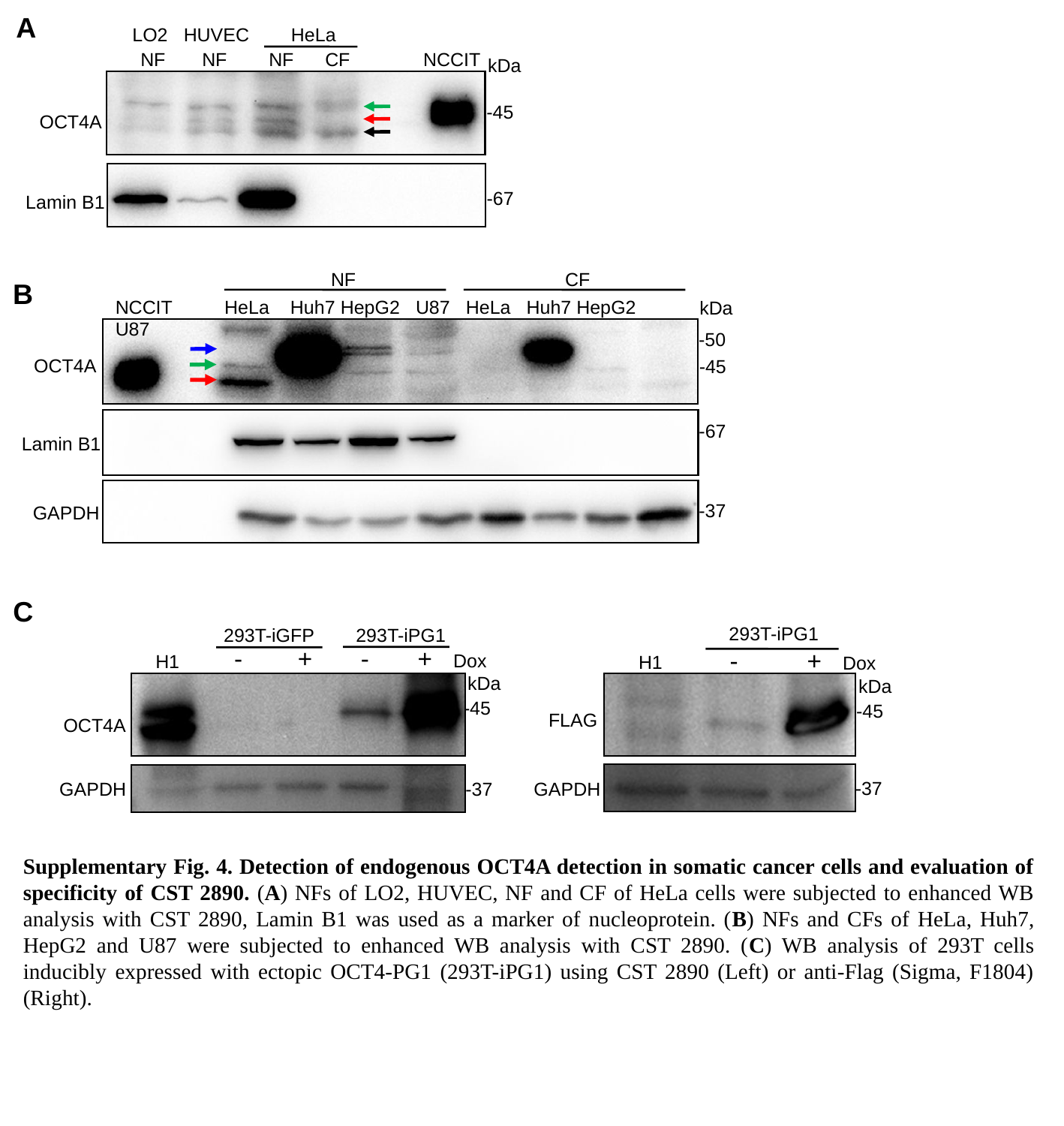

A
LO2 HUVEC HeLa
NF NF NF CF NCCIT
kDa
-45
OCT4A
-67
Lamin B1
NF CF
NCCIT HeLa Huh7 HepG2 U87 HeLa Huh7 HepG2 U87
kDa
-50
OCT4A
-45
-67
Lamin B1
-37
GAPDH
B
C
293T-iPG1
 - + Dox
H1
kDa
FLAG
GAPDH
-45
-37
293T-iGFP 293T-iPG1
- + - + Dox
H1
OCT4A
GAPDH
-45
-37
kDa
Supplementary Fig. 4. Detection of endogenous OCT4A detection in somatic cancer cells and evaluation of specificity of CST 2890. (A) NFs of LO2, HUVEC, NF and CF of HeLa cells were subjected to enhanced WB analysis with CST 2890, Lamin B1 was used as a marker of nucleoprotein. (B) NFs and CFs of HeLa, Huh7, HepG2 and U87 were subjected to enhanced WB analysis with CST 2890. (C) WB analysis of 293T cells inducibly expressed with ectopic OCT4-PG1 (293T-iPG1) using CST 2890 (Left) or anti-Flag (Sigma, F1804) (Right).

## Slide 7
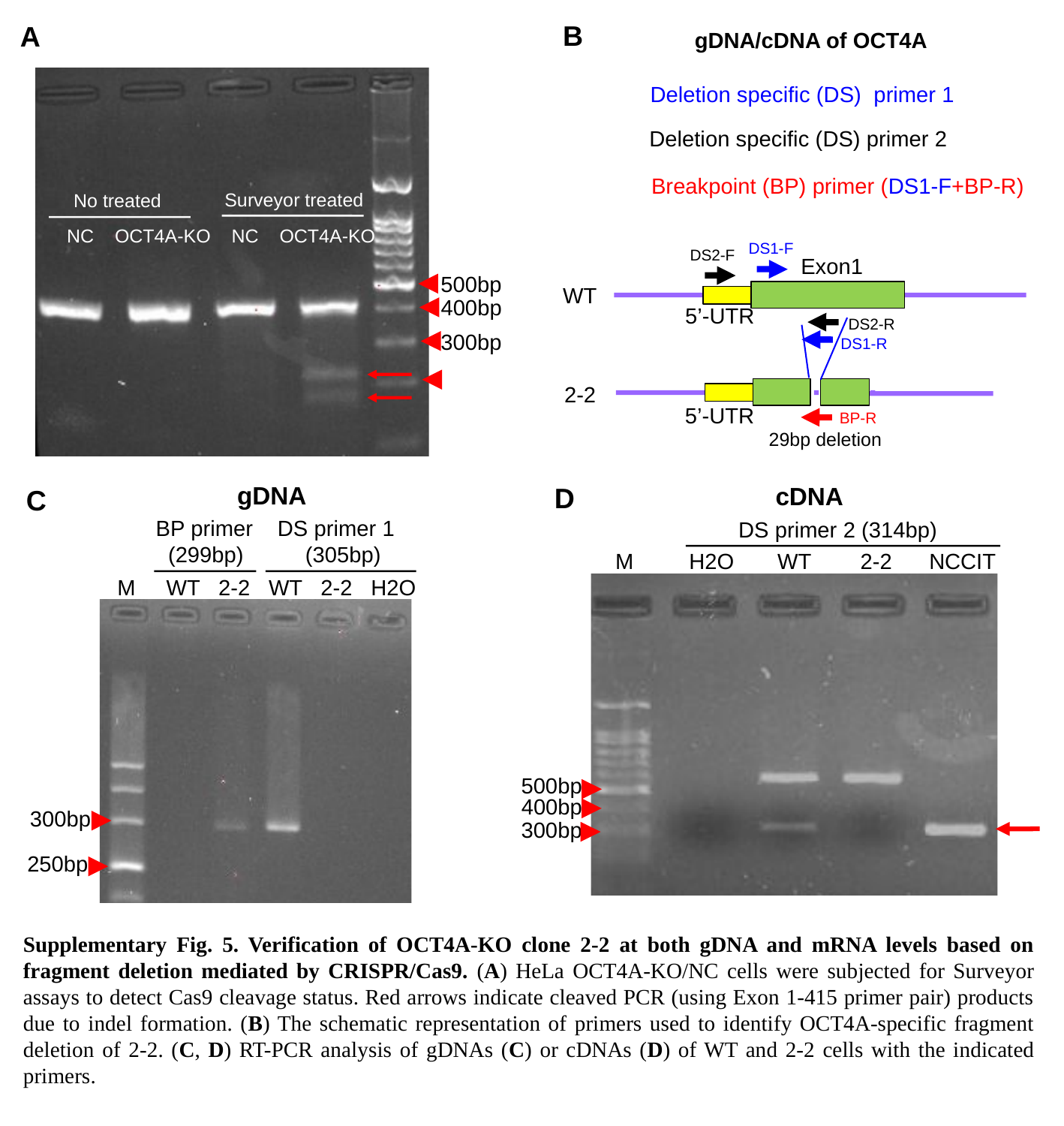

B
A
gDNA/cDNA of OCT4A
Exon1
WT
5’-UTR
2-2
29bp deletion
Deletion specific (DS) primer 1
Deletion specific (DS) primer 2
Breakpoint (BP) primer (DS1-F+BP-R)
5’-UTR
DS1-R
DS1-F
DS2-F
BP-R
DS2-R
Surveyor treated
No treated
NC OCT4A-KO NC OCT4A-KO
500bp
400bp
300bp
gDNA
BP primer DS primer 1
 (299bp) (305bp)
M WT 2-2 WT 2-2 H2O
300bp
250bp
D
cDNA
C
DS primer 2 (314bp)
M H2O WT 2-2 NCCIT
500bp
400bp
300bp
Supplementary Fig. 5. Verification of OCT4A-KO clone 2-2 at both gDNA and mRNA levels based on fragment deletion mediated by CRISPR/Cas9. (A) HeLa OCT4A-KO/NC cells were subjected for Surveyor assays to detect Cas9 cleavage status. Red arrows indicate cleaved PCR (using Exon 1-415 primer pair) products due to indel formation. (B) The schematic representation of primers used to identify OCT4A-specific fragment deletion of 2-2. (C, D) RT-PCR analysis of gDNAs (C) or cDNAs (D) of WT and 2-2 cells with the indicated primers.

## Slide 8
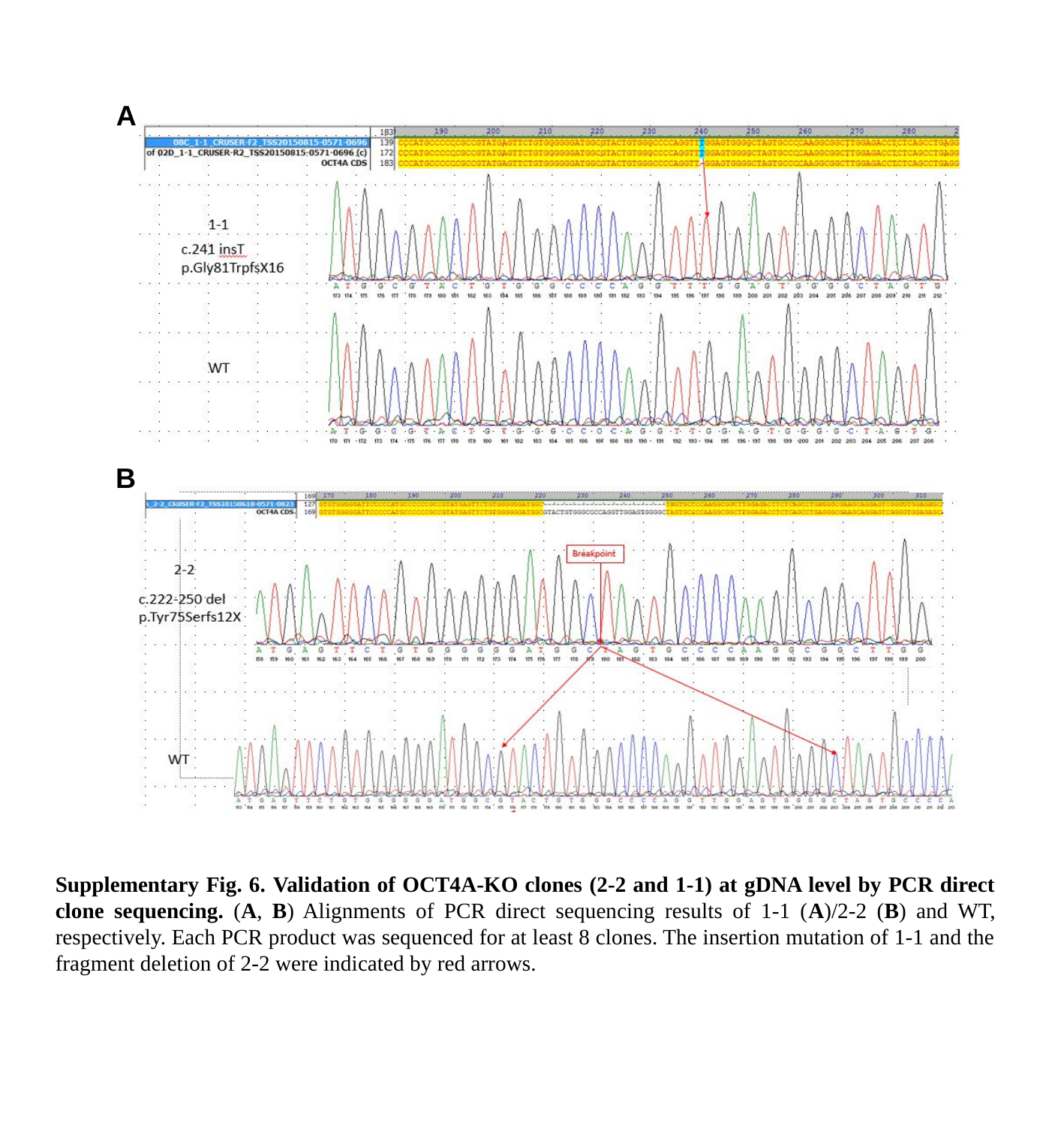

A
B
Supplementary Fig. 6. Validation of OCT4A-KO clones (2-2 and 1-1) at gDNA level by PCR direct clone sequencing. (A, B) Alignments of PCR direct sequencing results of 1-1 (A)/2-2 (B) and WT, respectively. Each PCR product was sequenced for at least 8 clones. The insertion mutation of 1-1 and the fragment deletion of 2-2 were indicated by red arrows.

## Slide 9
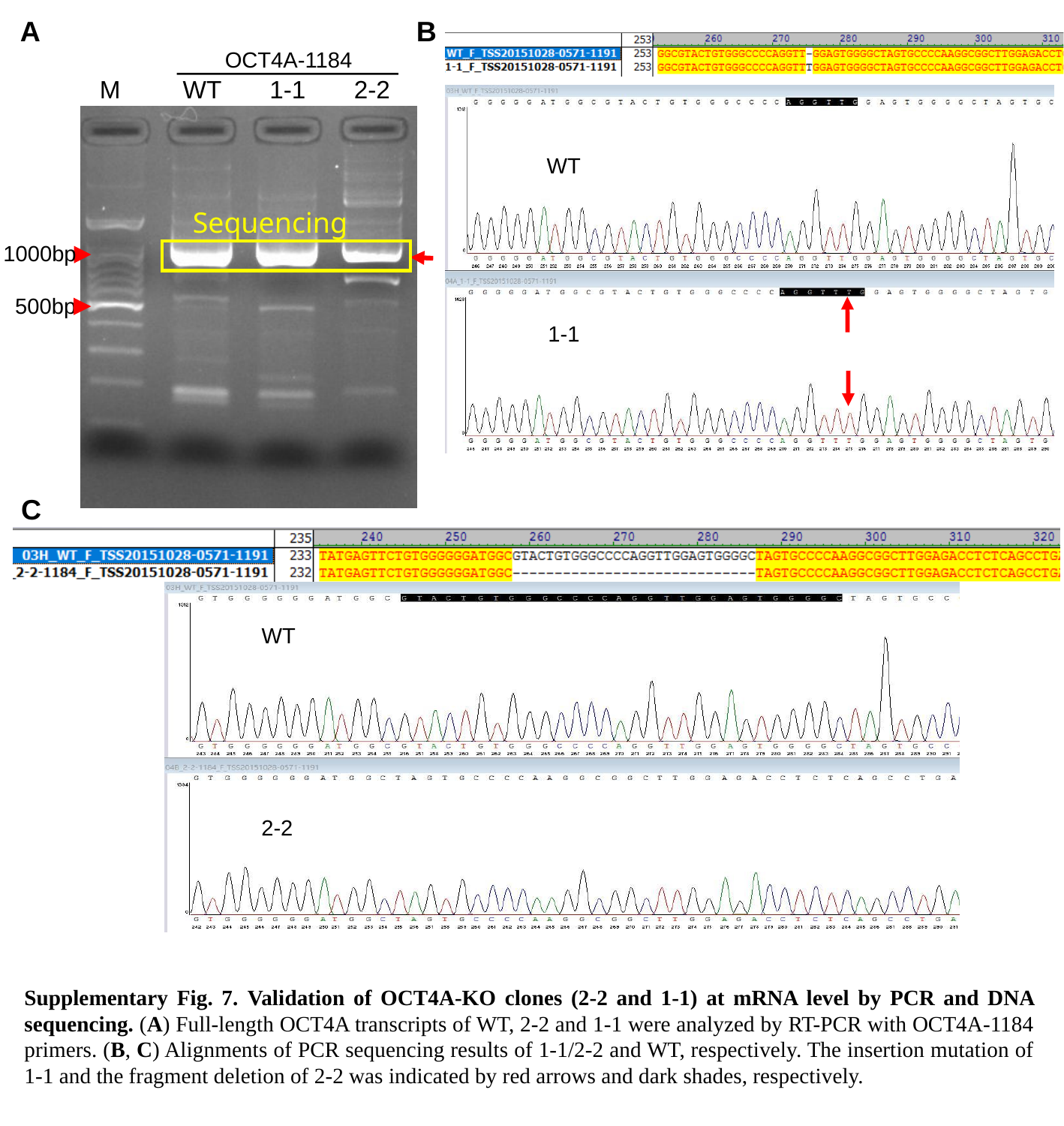

A
B
WT
1-1
OCT4A-1184
M WT 1-1 2-2
Sequencing
1000bp
500bp
C
WT
2-2
Supplementary Fig. 7. Validation of OCT4A-KO clones (2-2 and 1-1) at mRNA level by PCR and DNA sequencing. (A) Full-length OCT4A transcripts of WT, 2-2 and 1-1 were analyzed by RT-PCR with OCT4A-1184 primers. (B, C) Alignments of PCR sequencing results of 1-1/2-2 and WT, respectively. The insertion mutation of 1-1 and the fragment deletion of 2-2 was indicated by red arrows and dark shades, respectively.

## Slide 10
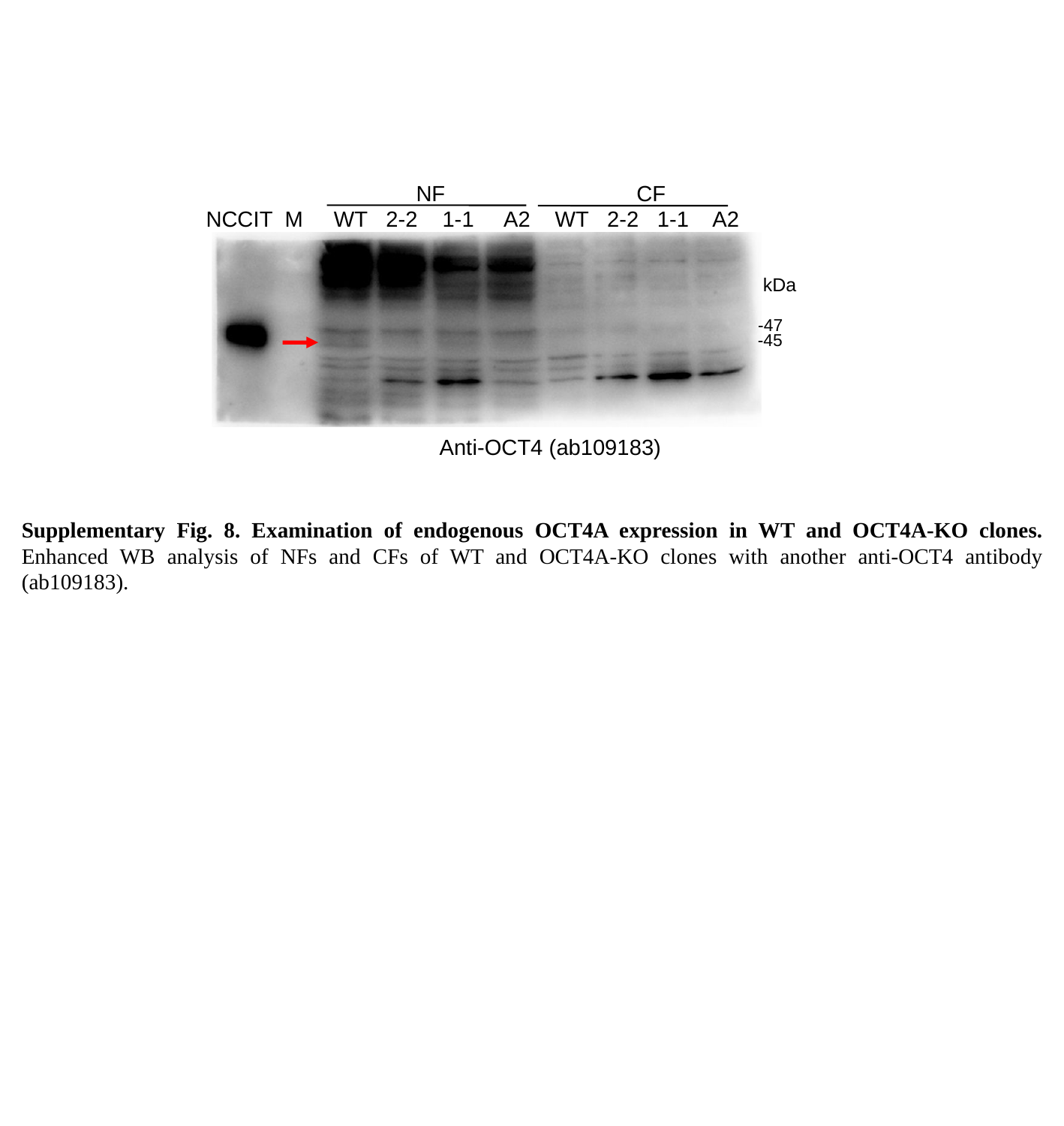

NF CF
NCCIT M WT 2-2 1-1 A2 WT 2-2 1-1 A2
Anti-OCT4 (ab109183)
kDa
-47
-45
Supplementary Fig. 8. Examination of endogenous OCT4A expression in WT and OCT4A-KO clones. Enhanced WB analysis of NFs and CFs of WT and OCT4A-KO clones with another anti-OCT4 antibody (ab109183).

## Slide 11
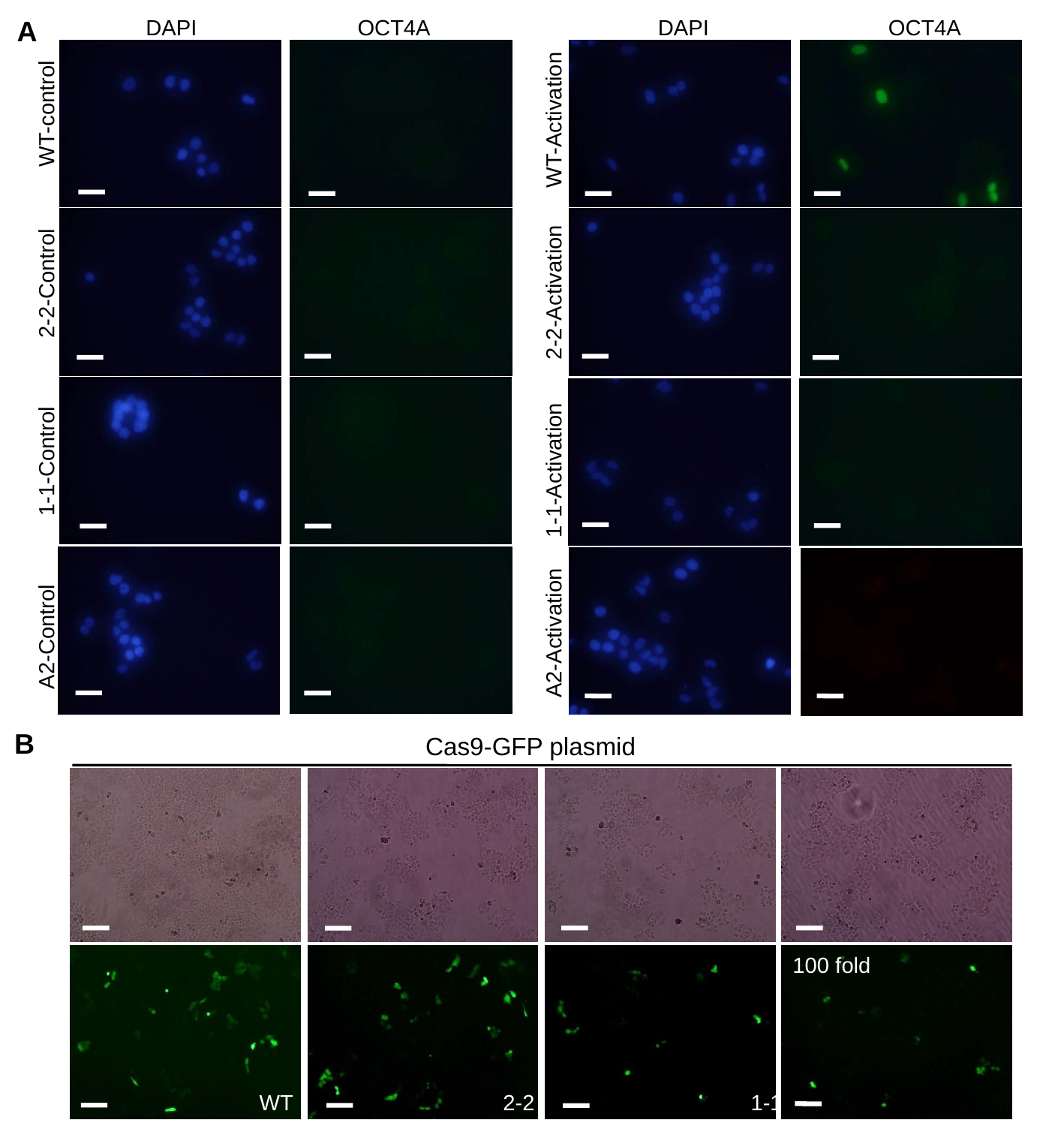

A
DAPI OCT4A DAPI OCT4A
A2-Control 1-1-Control 2-2-Control WT-control
A2-Activation 1-1-Activation 2-2-Activation WT-Activation
B
Cas9-GFP plasmid
100 fold
WT 2-2 1-1 A2

## Slide 12
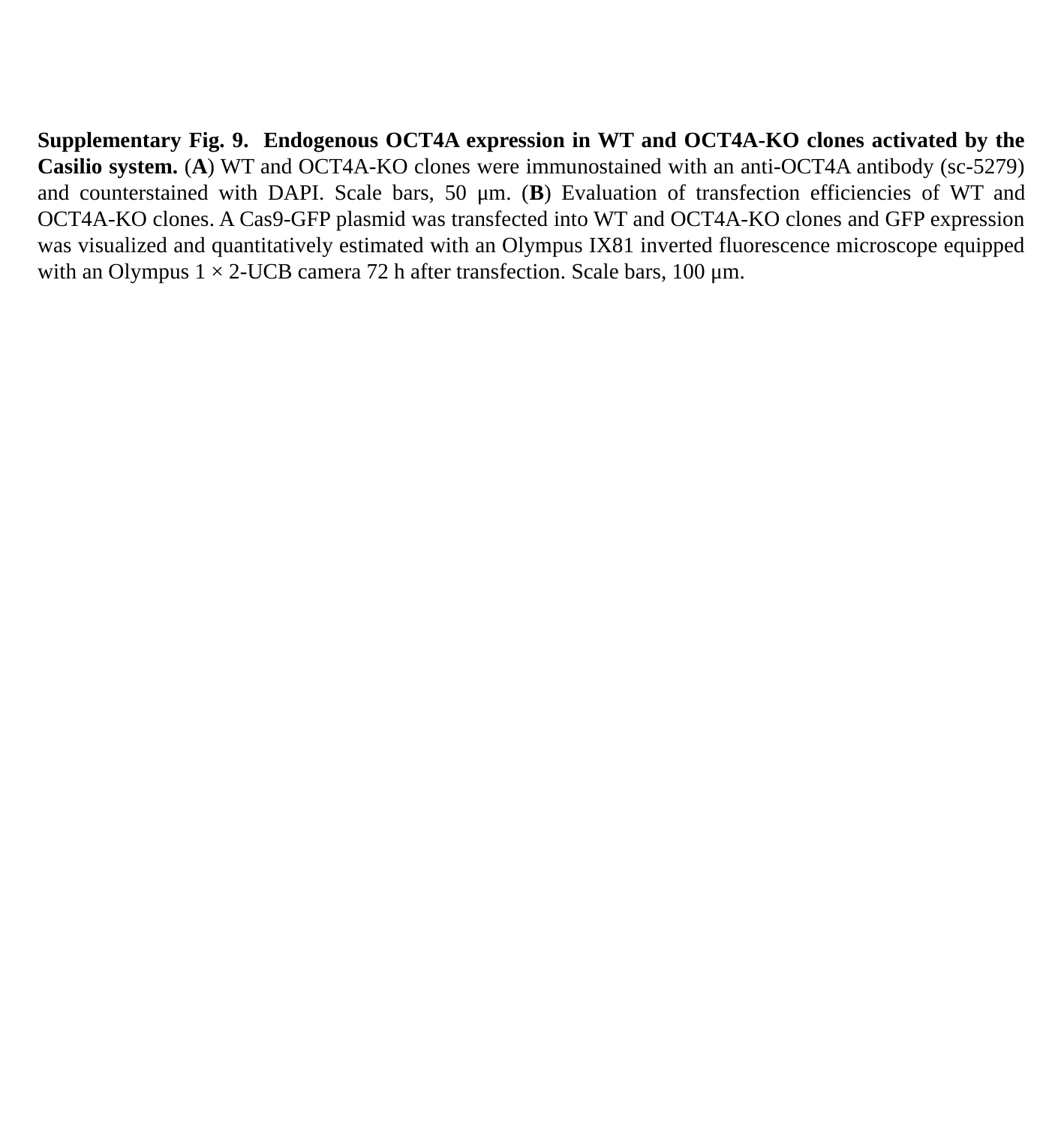

Supplementary Fig. 9. Endogenous OCT4A expression in WT and OCT4A-KO clones activated by the Casilio system. (A) WT and OCT4A-KO clones were immunostained with an anti-OCT4A antibody (sc-5279) and counterstained with DAPI. Scale bars, 50 μm. (B) Evaluation of transfection efficiencies of WT and OCT4A-KO clones. A Cas9-GFP plasmid was transfected into WT and OCT4A-KO clones and GFP expression was visualized and quantitatively estimated with an Olympus IX81 inverted fluorescence microscope equipped with an Olympus 1 × 2-UCB camera 72 h after transfection. Scale bars, 100 μm.

## Slide 13
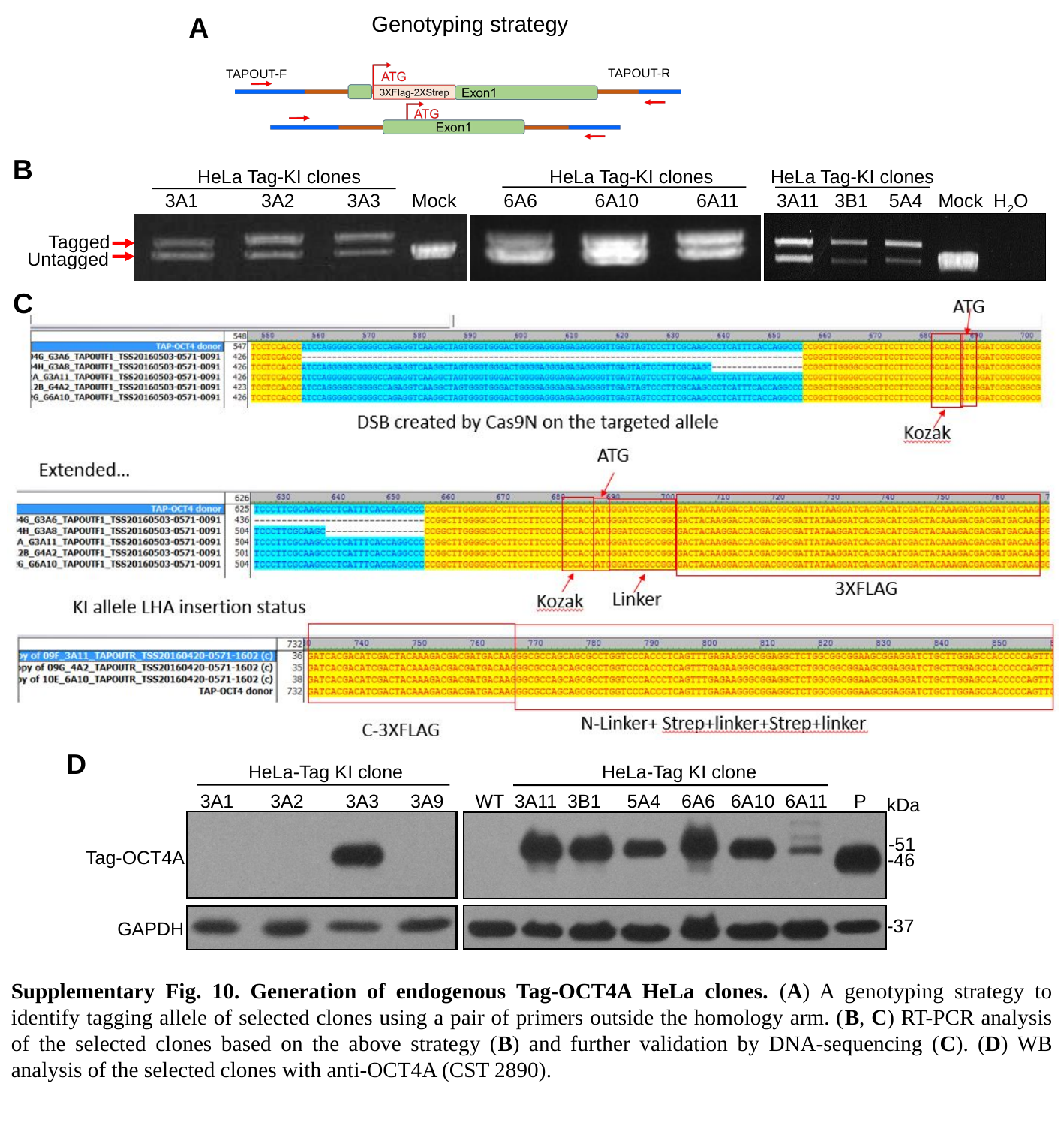

A
Genotyping strategy
TAPOUT-R
TAPOUT-F
B
HeLa Tag-KI clones HeLa Tag-KI clones HeLa Tag-KI clones
3A11 3B1 5A4 Mock H2O
Tagged
Untagged
3A1 3A2 3A3 Mock 6A6 6A10 6A11
C
D
HeLa-Tag KI clone HeLa-Tag KI clone
3A1 3A2 3A3 3A9 WT 3A11 3B1 5A4 6A6 6A10 6A11 P
kDa
-51
Tag-OCT4A
-46
-37
GAPDH
Supplementary Fig. 10. Generation of endogenous Tag-OCT4A HeLa clones. (A) A genotyping strategy to identify tagging allele of selected clones using a pair of primers outside the homology arm. (B, C) RT-PCR analysis of the selected clones based on the above strategy (B) and further validation by DNA-sequencing (C). (D) WB analysis of the selected clones with anti-OCT4A (CST 2890).

## Slide 14
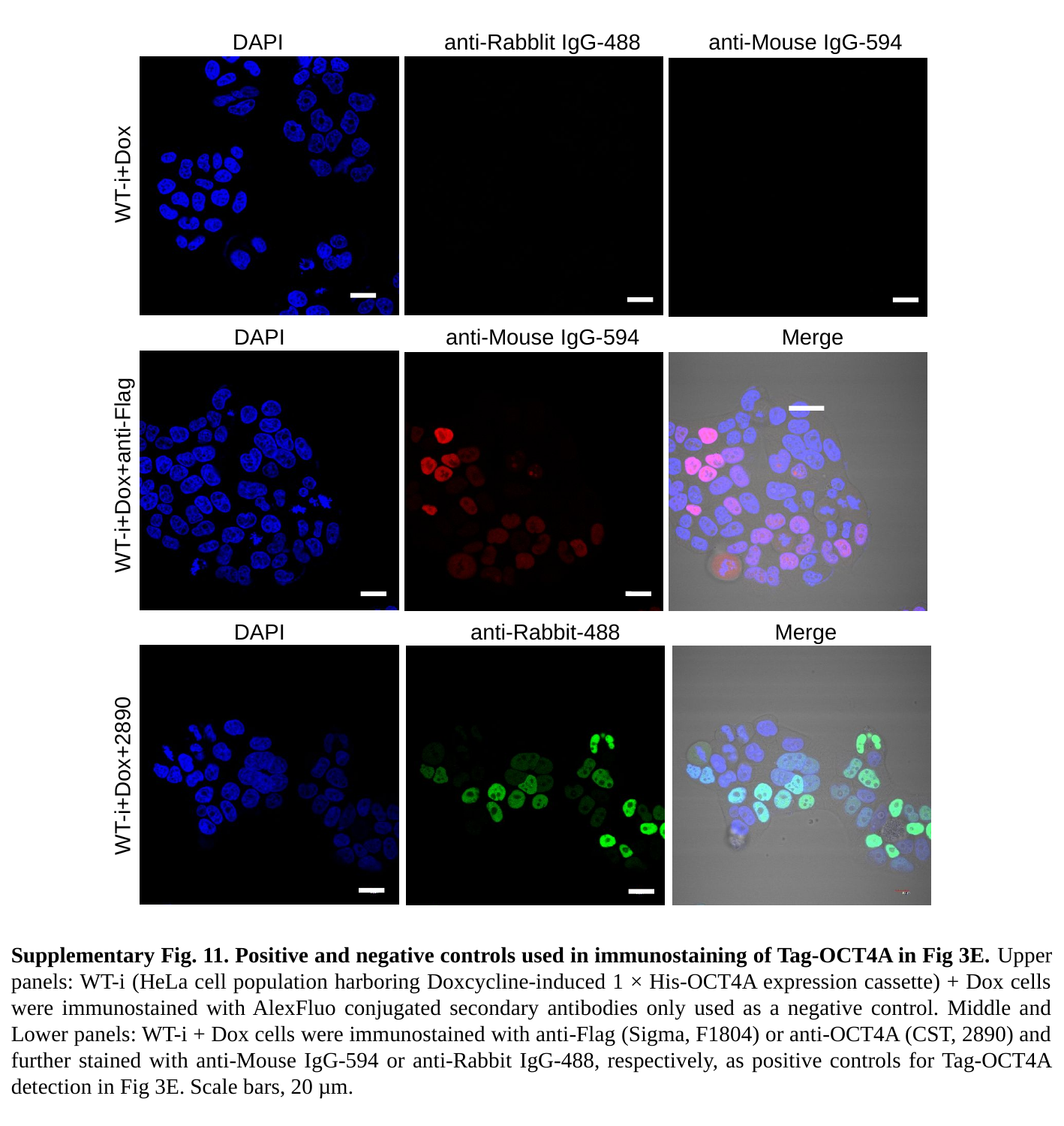

DAPI anti-Rabblit IgG-488 anti-Mouse IgG-594
DAPI anti-Mouse IgG-594 Merge
WT-i+Dox+2890 WT-i+Dox+anti-Flag WT-i+Dox
DAPI anti-Rabbit-488 Merge
Supplementary Fig. 11. Positive and negative controls used in immunostaining of Tag-OCT4A in Fig 3E. Upper panels: WT-i (HeLa cell population harboring Doxcycline-induced 1 × His-OCT4A expression cassette) + Dox cells were immunostained with AlexFluo conjugated secondary antibodies only used as a negative control. Middle and Lower panels: WT-i + Dox cells were immunostained with anti-Flag (Sigma, F1804) or anti-OCT4A (CST, 2890) and further stained with anti-Mouse IgG-594 or anti-Rabbit IgG-488, respectively, as positive controls for Tag-OCT4A detection in Fig 3E. Scale bars, 20 µm.

## Slide 15
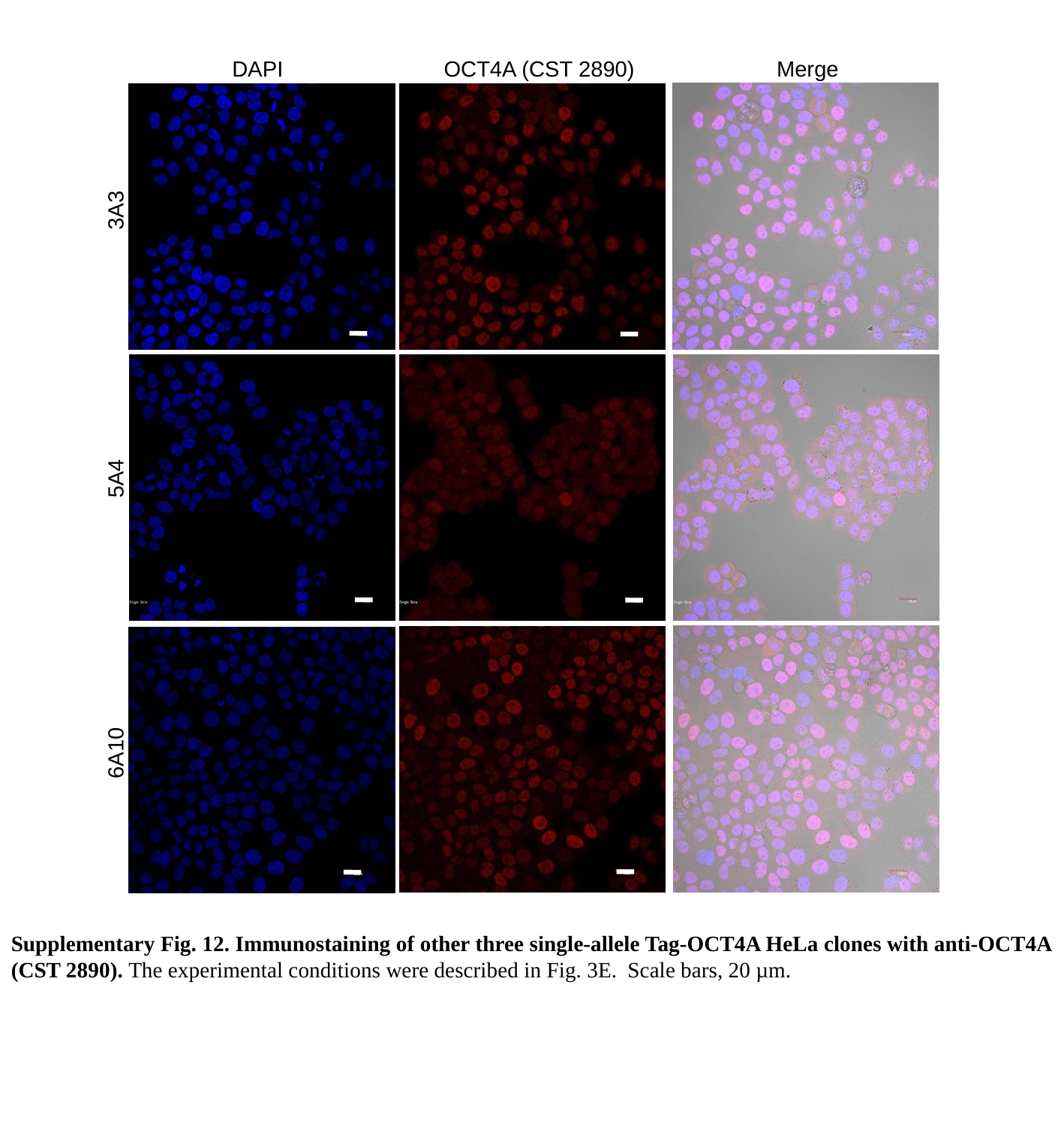

DAPI OCT4A (CST 2890) Merge
6A10 5A4 3A3
Supplementary Fig. 12. Immunostaining of other three single-allele Tag-OCT4A HeLa clones with anti-OCT4A (CST 2890). The experimental conditions were described in Fig. 3E. Scale bars, 20 µm.

## Slide 16
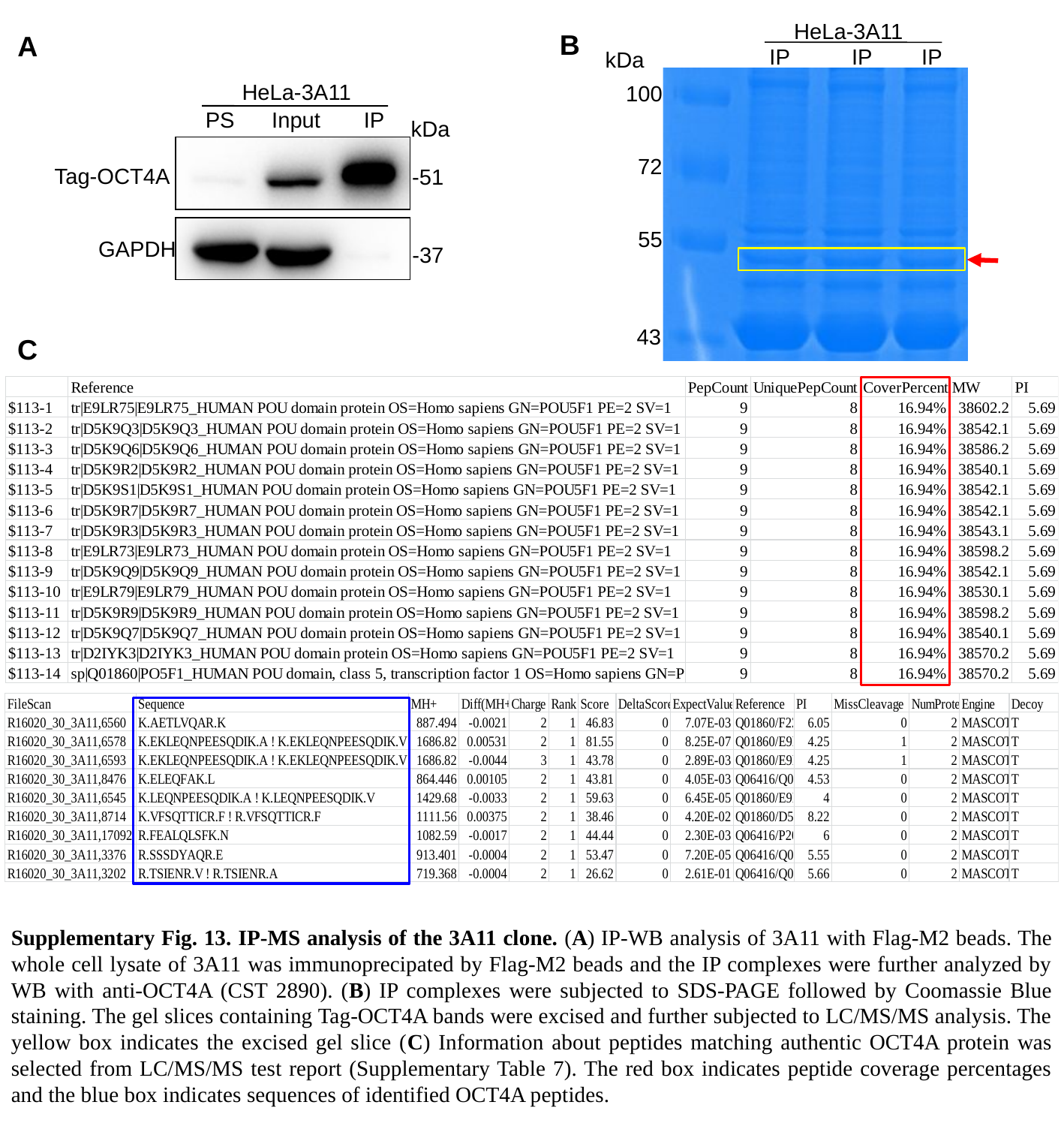

HeLa-3A11
IP IP IP
kDa
72
55
43
100
B
A
HeLa-3A11
PS Input IP
Tag-OCT4A
GAPDH
kDa
-51
-37
C
Supplementary Fig. 13. IP-MS analysis of the 3A11 clone. (A) IP-WB analysis of 3A11 with Flag-M2 beads. The whole cell lysate of 3A11 was immunoprecipated by Flag-M2 beads and the IP complexes were further analyzed by WB with anti-OCT4A (CST 2890). (B) IP complexes were subjected to SDS-PAGE followed by Coomassie Blue staining. The gel slices containing Tag-OCT4A bands were excised and further subjected to LC/MS/MS analysis. The yellow box indicates the excised gel slice (C) Information about peptides matching authentic OCT4A protein was selected from LC/MS/MS test report (Supplementary Table 7). The red box indicates peptide coverage percentages and the blue box indicates sequences of identified OCT4A peptides.

## Slide 17
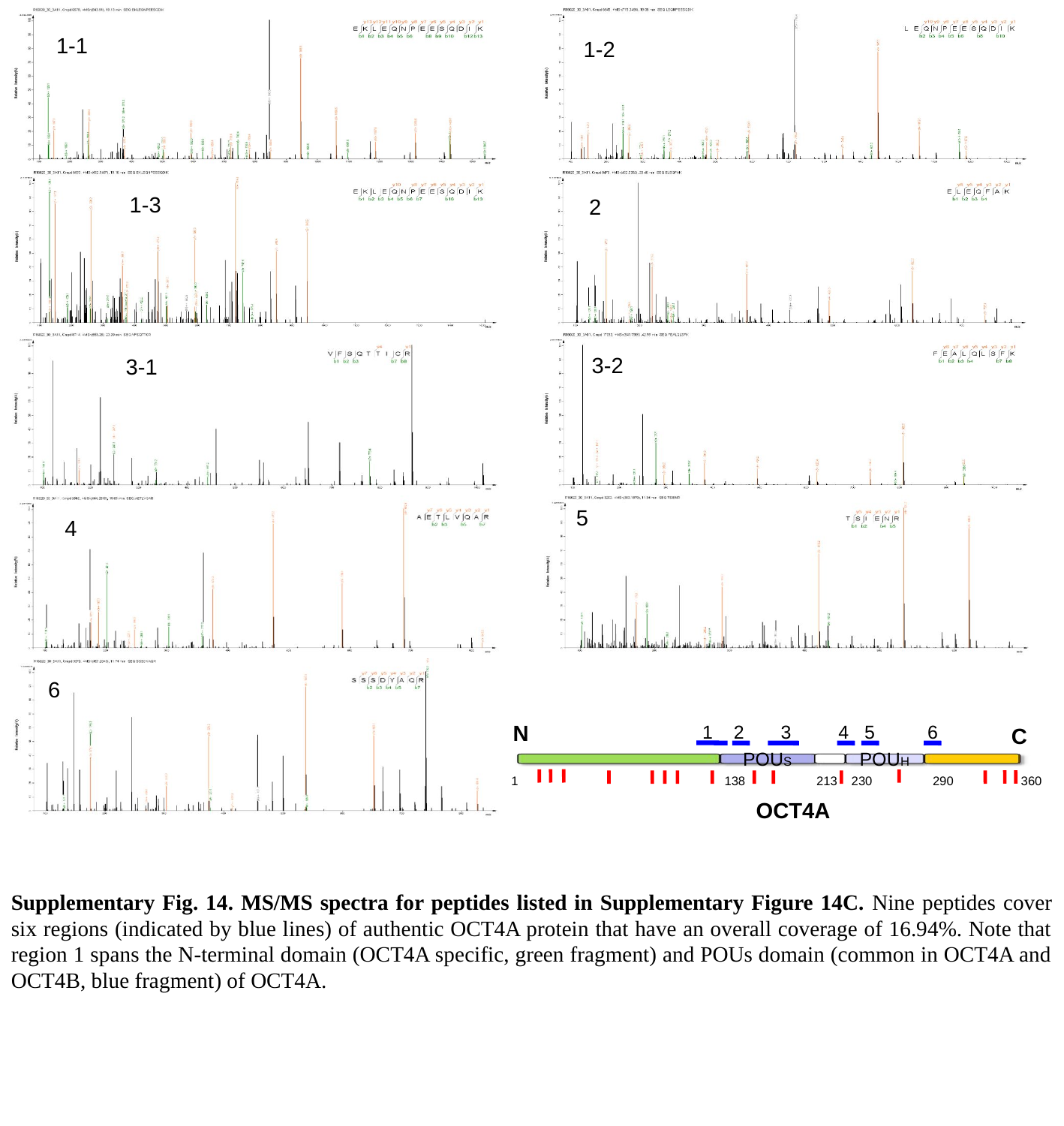

1-1
1-2
1-3
2
3-2
3-1
5
4
6
N
C
POUS
POUH
1 138 213 230 290 360
OCT4A
1 2 3 4 5 6
Supplementary Fig. 14. MS/MS spectra for peptides listed in Supplementary Figure 14C. Nine peptides cover six regions (indicated by blue lines) of authentic OCT4A protein that have an overall coverage of 16.94%. Note that region 1 spans the N-terminal domain (OCT4A specific, green fragment) and POUs domain (common in OCT4A and OCT4B, blue fragment) of OCT4A.

## Slide 18
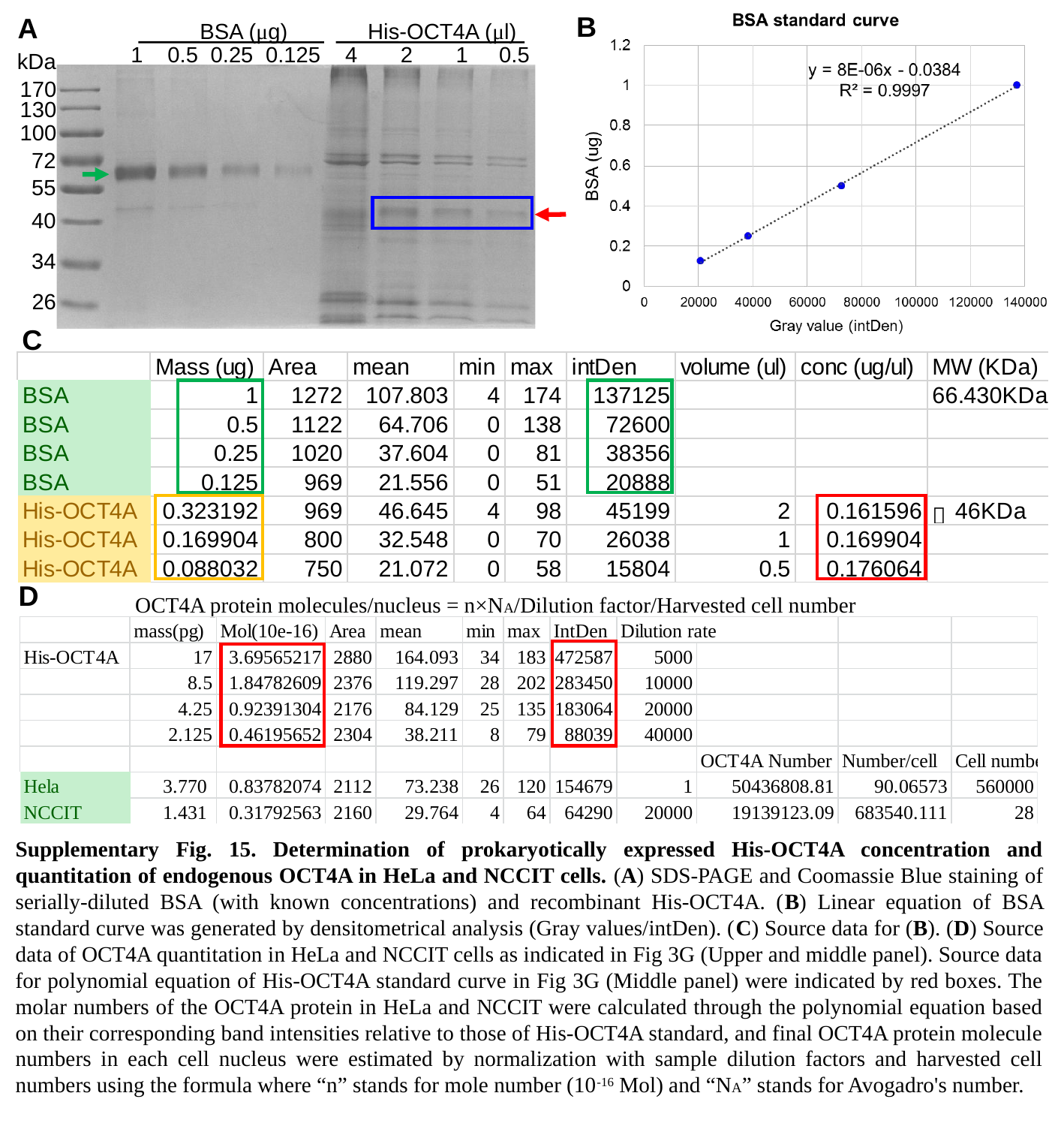

B
A
BSA (μg) His-OCT4A (μl)
1 0.5 0.25 0.125 4 2 1 0.5
170
130
100
72
55
40
34
26
kDa
C
D
OCT4A protein molecules/nucleus = n×NA/Dilution factor/Harvested cell number
Supplementary Fig. 15. Determination of prokaryotically expressed His-OCT4A concentration and quantitation of endogenous OCT4A in HeLa and NCCIT cells. (A) SDS-PAGE and Coomassie Blue staining of serially-diluted BSA (with known concentrations) and recombinant His-OCT4A. (B) Linear equation of BSA standard curve was generated by densitometrical analysis (Gray values/intDen). (C) Source data for (B). (D) Source data of OCT4A quantitation in HeLa and NCCIT cells as indicated in Fig 3G (Upper and middle panel). Source data for polynomial equation of His-OCT4A standard curve in Fig 3G (Middle panel) were indicated by red boxes. The molar numbers of the OCT4A protein in HeLa and NCCIT were calculated through the polynomial equation based on their corresponding band intensities relative to those of His-OCT4A standard, and final OCT4A protein molecule numbers in each cell nucleus were estimated by normalization with sample dilution factors and harvested cell numbers using the formula where “n” stands for mole number (10-16 Mol) and “NA” stands for Avogadro's number.

## Slide 19
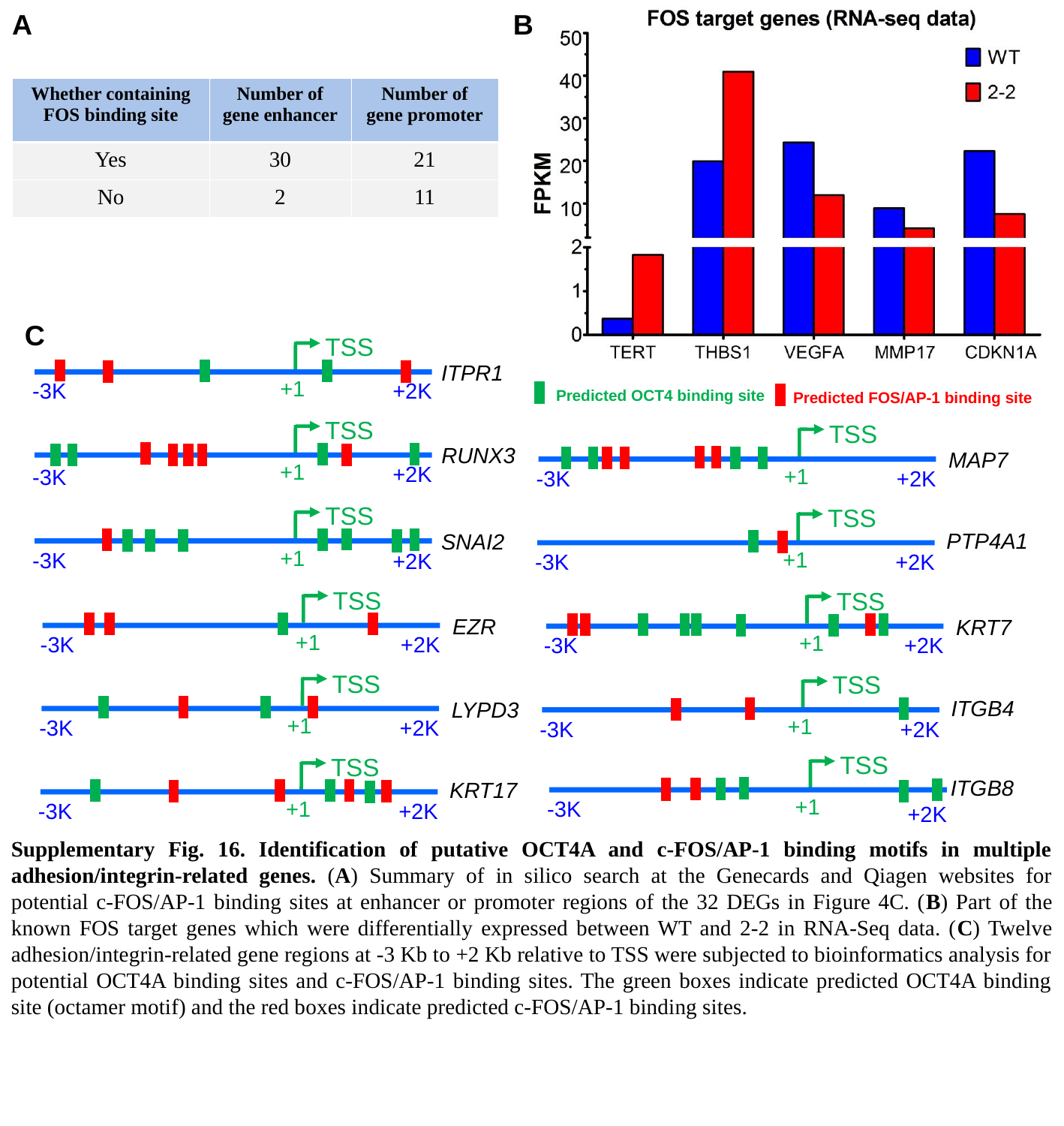

A
B
| Whether containing FOS binding site | Number of gene enhancer | Number of gene promoter |
| --- | --- | --- |
| Yes | 30 | 21 |
| No | 2 | 11 |
C
TSS
ITPR1
+1
-3K
+2K
Predicted OCT4 binding site
Predicted FOS/AP-1 binding site
TSS
RUNX3
+1
+2K
-3K
TSS
MAP7
+1
-3K
+2K
TSS
SNAI2
+1
-3K
+2K
TSS
PTP4A1
+1
-3K
+2K
TSS
EZR
+1
-3K
+2K
TSS
KRT7
+1
-3K
+2K
TSS
LYPD3
+1
-3K
+2K
TSS
ITGB4
+1
-3K
+2K
TSS
ITGB8
+1
-3K
+2K
TSS
KRT17
+1
-3K
+2K
Supplementary Fig. 16. Identification of putative OCT4A and c-FOS/AP-1 binding motifs in multiple adhesion/integrin-related genes. (A) Summary of in silico search at the Genecards and Qiagen websites for potential c-FOS/AP-1 binding sites at enhancer or promoter regions of the 32 DEGs in Figure 4C. (B) Part of the known FOS target genes which were differentially expressed between WT and 2-2 in RNA-Seq data. (C) Twelve adhesion/integrin-related gene regions at -3 Kb to +2 Kb relative to TSS were subjected to bioinformatics analysis for potential OCT4A binding sites and c-FOS/AP-1 binding sites. The green boxes indicate predicted OCT4A binding site (octamer motif) and the red boxes indicate predicted c-FOS/AP-1 binding sites.

## Slide 20
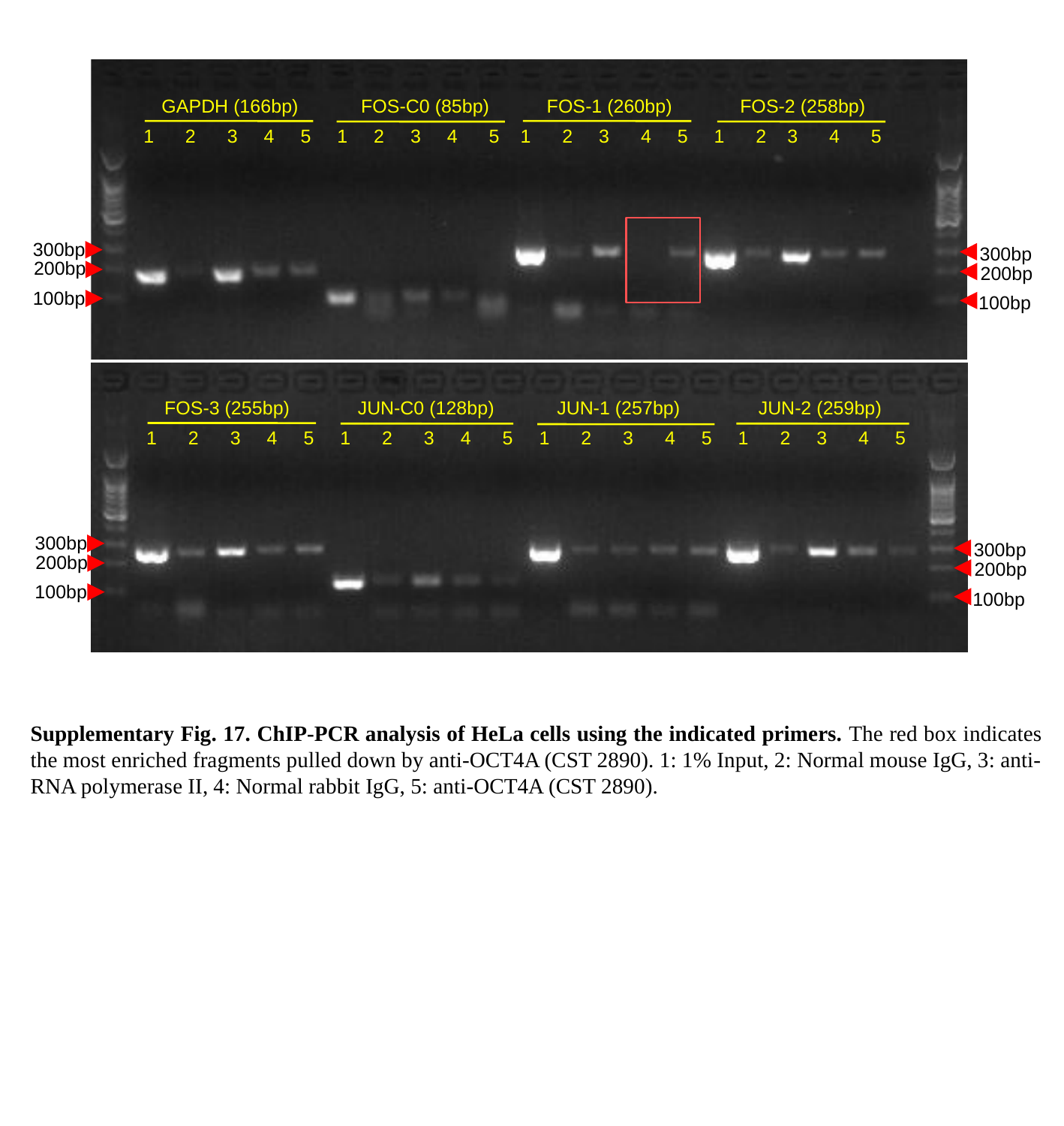

GAPDH (166bp) FOS-C0 (85bp) FOS-1 (260bp) FOS-2 (258bp)
1 2 3 4 5 1 2 3 4 5 1 2 3 4 5 1 2 3 4 5
300bp
200bp
100bp
300bp
200bp
100bp
FOS-3 (255bp) JUN-C0 (128bp) JUN-1 (257bp) JUN-2 (259bp)
1 2 3 4 5 1 2 3 4 5 1 2 3 4 5 1 2 3 4 5
300bp
200bp
100bp
300bp
200bp
100bp
Supplementary Fig. 17. ChIP-PCR analysis of HeLa cells using the indicated primers. The red box indicates the most enriched fragments pulled down by anti-OCT4A (CST 2890). 1: 1% Input, 2: Normal mouse IgG, 3: anti-RNA polymerase II, 4: Normal rabbit IgG, 5: anti-OCT4A (CST 2890).

## Slide 21
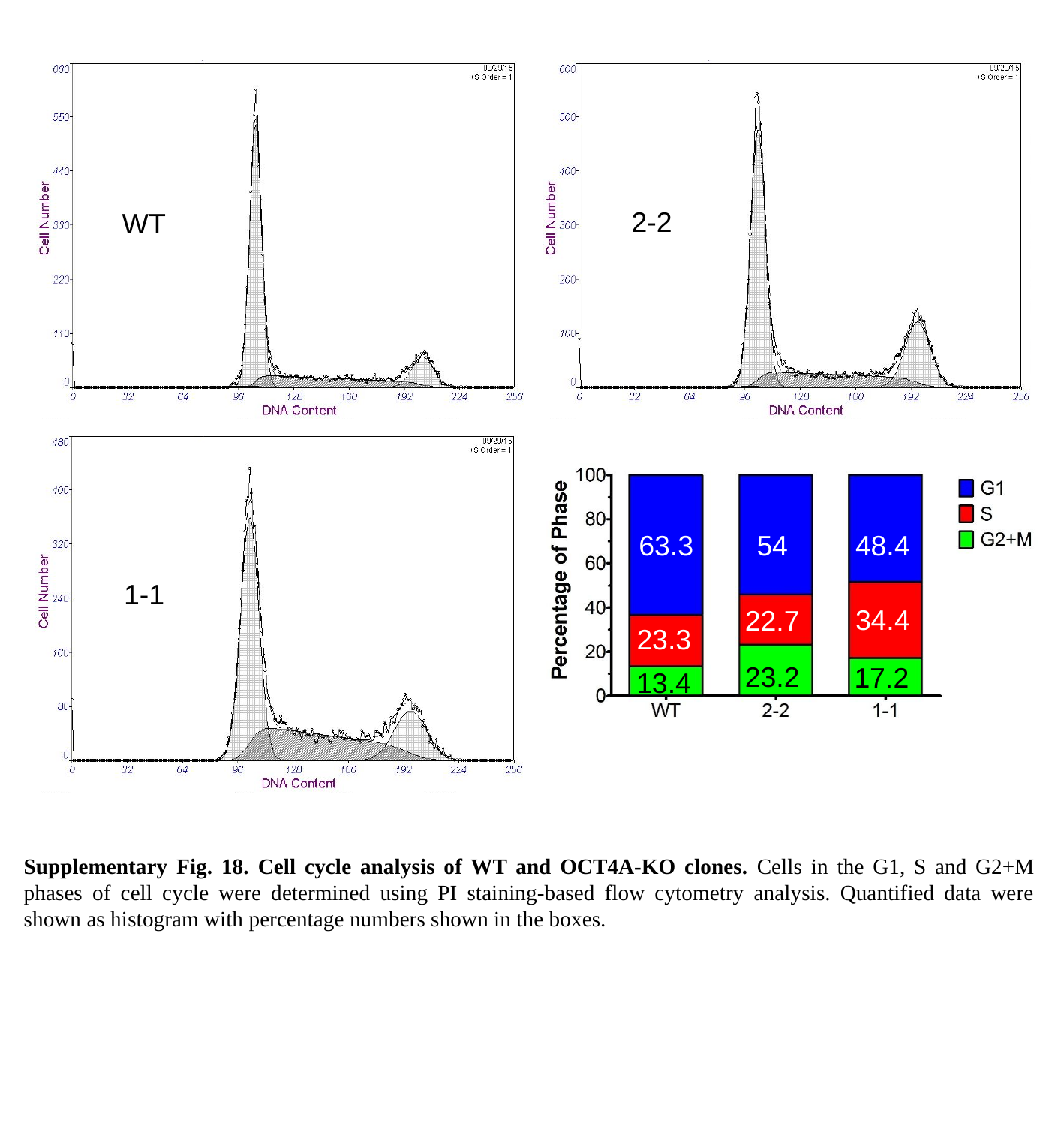

2-2
WT
1-1
63.3
54
48.4
34.4
22.7
23.3
23.2
17.2
13.4
Supplementary Fig. 18. Cell cycle analysis of WT and OCT4A-KO clones. Cells in the G1, S and G2+M phases of cell cycle were determined using PI staining-based flow cytometry analysis. Quantified data were shown as histogram with percentage numbers shown in the boxes.

## Slide 22
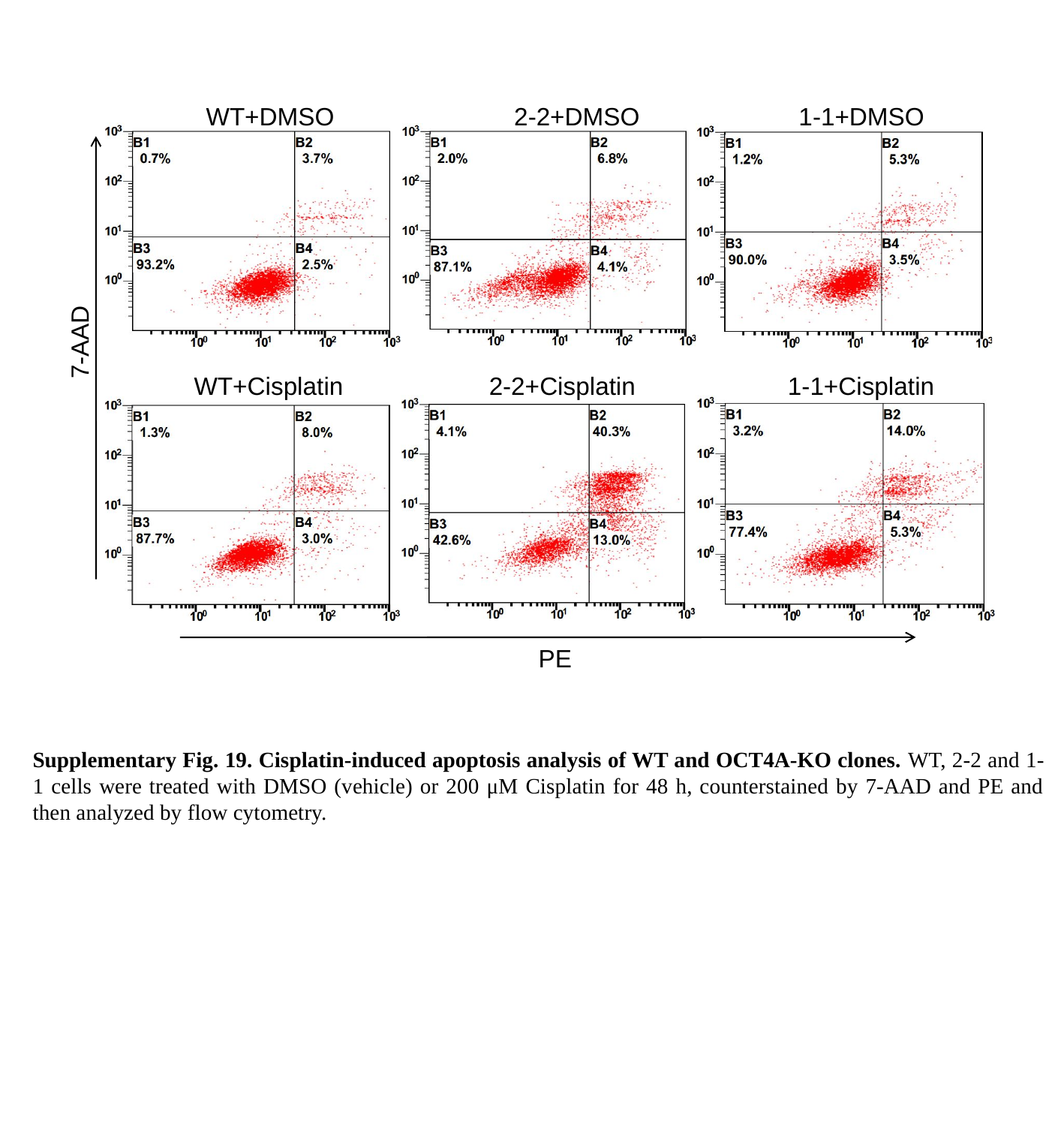

WT+DMSO 2-2+DMSO 1-1+DMSO
WT+Cisplatin 2-2+Cisplatin 1-1+Cisplatin
7-AAD
PE
Supplementary Fig. 19. Cisplatin-induced apoptosis analysis of WT and OCT4A-KO clones. WT, 2-2 and 1-1 cells were treated with DMSO (vehicle) or 200 μM Cisplatin for 48 h, counterstained by 7-AAD and PE and then analyzed by flow cytometry.

## Slide 23
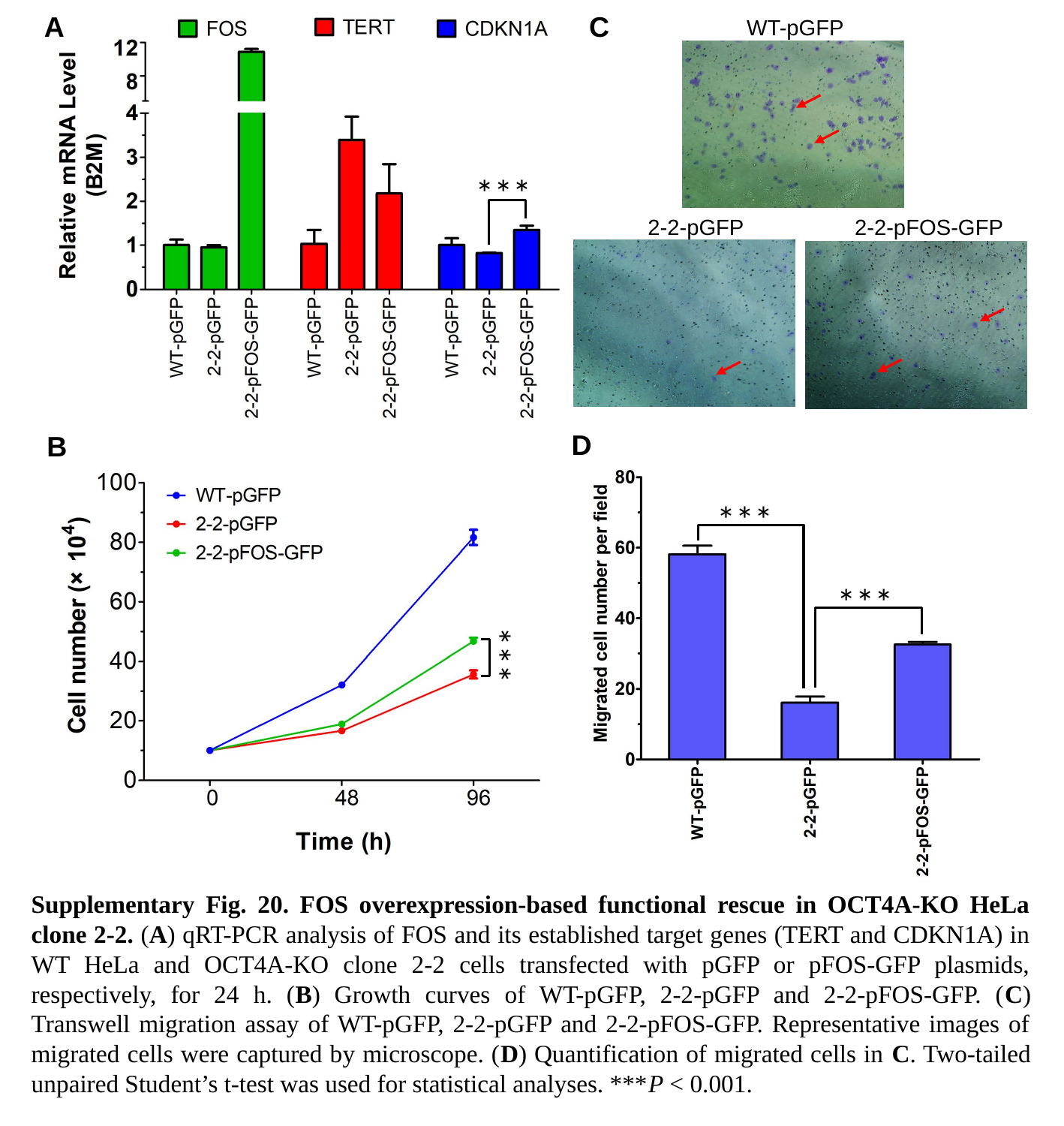

A
C
WT-pGFP
2-2-pGFP 2-2-pFOS-GFP
***
D
B
***
***
***
Supplementary Fig. 20. FOS overexpression-based functional rescue in OCT4A-KO HeLa clone 2-2. (A) qRT-PCR analysis of FOS and its established target genes (TERT and CDKN1A) in WT HeLa and OCT4A-KO clone 2-2 cells transfected with pGFP or pFOS-GFP plasmids, respectively, for 24 h. (B) Growth curves of WT-pGFP, 2-2-pGFP and 2-2-pFOS-GFP. (C) Transwell migration assay of WT-pGFP, 2-2-pGFP and 2-2-pFOS-GFP. Representative images of migrated cells were captured by microscope. (D) Quantification of migrated cells in C. Two-tailed unpaired Student’s t-test was used for statistical analyses. ***P < 0.001.

## Slide 24
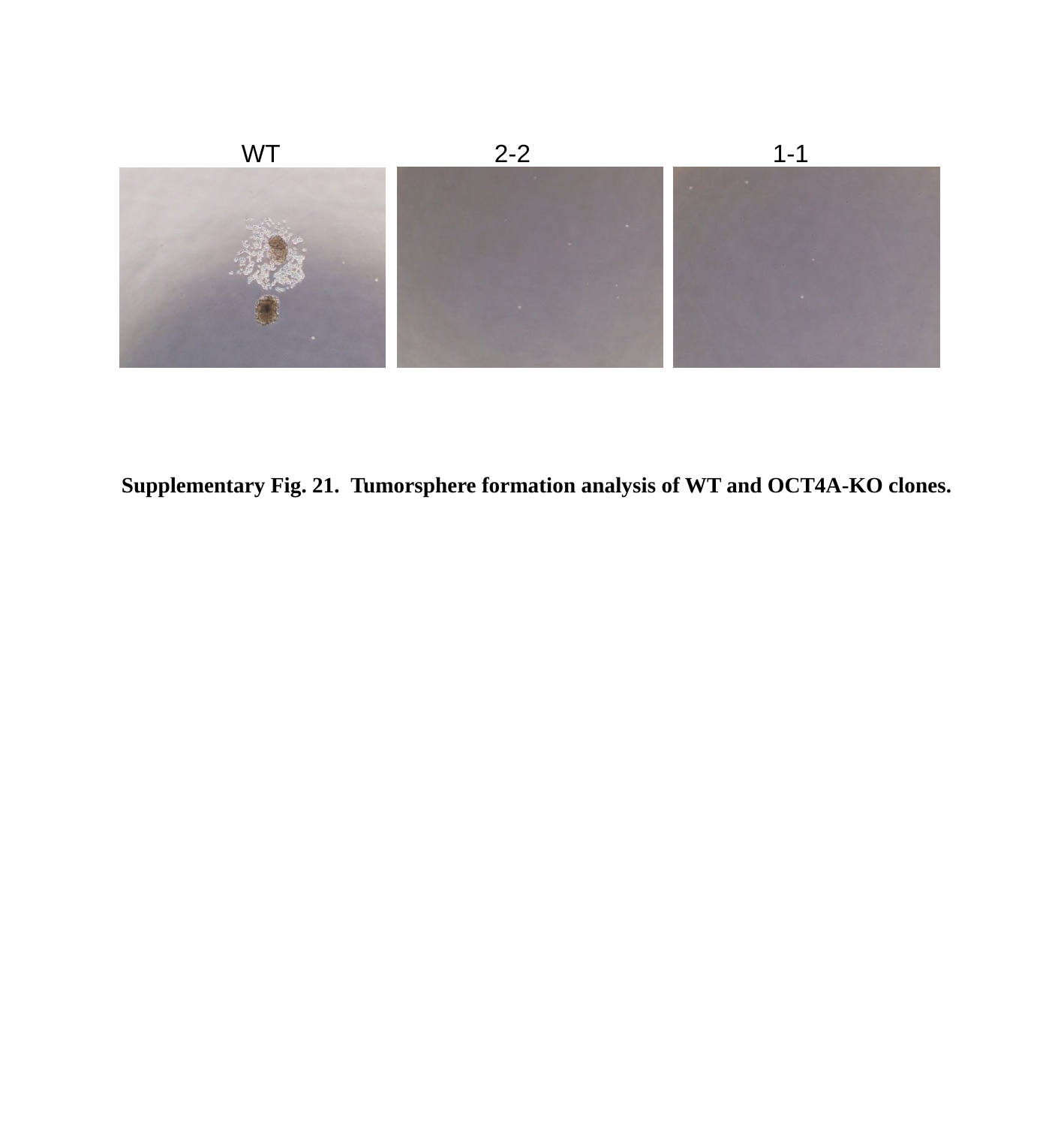

WT 2-2 1-1
Supplementary Fig. 21. Tumorsphere formation analysis of WT and OCT4A-KO clones.

## Slide 25
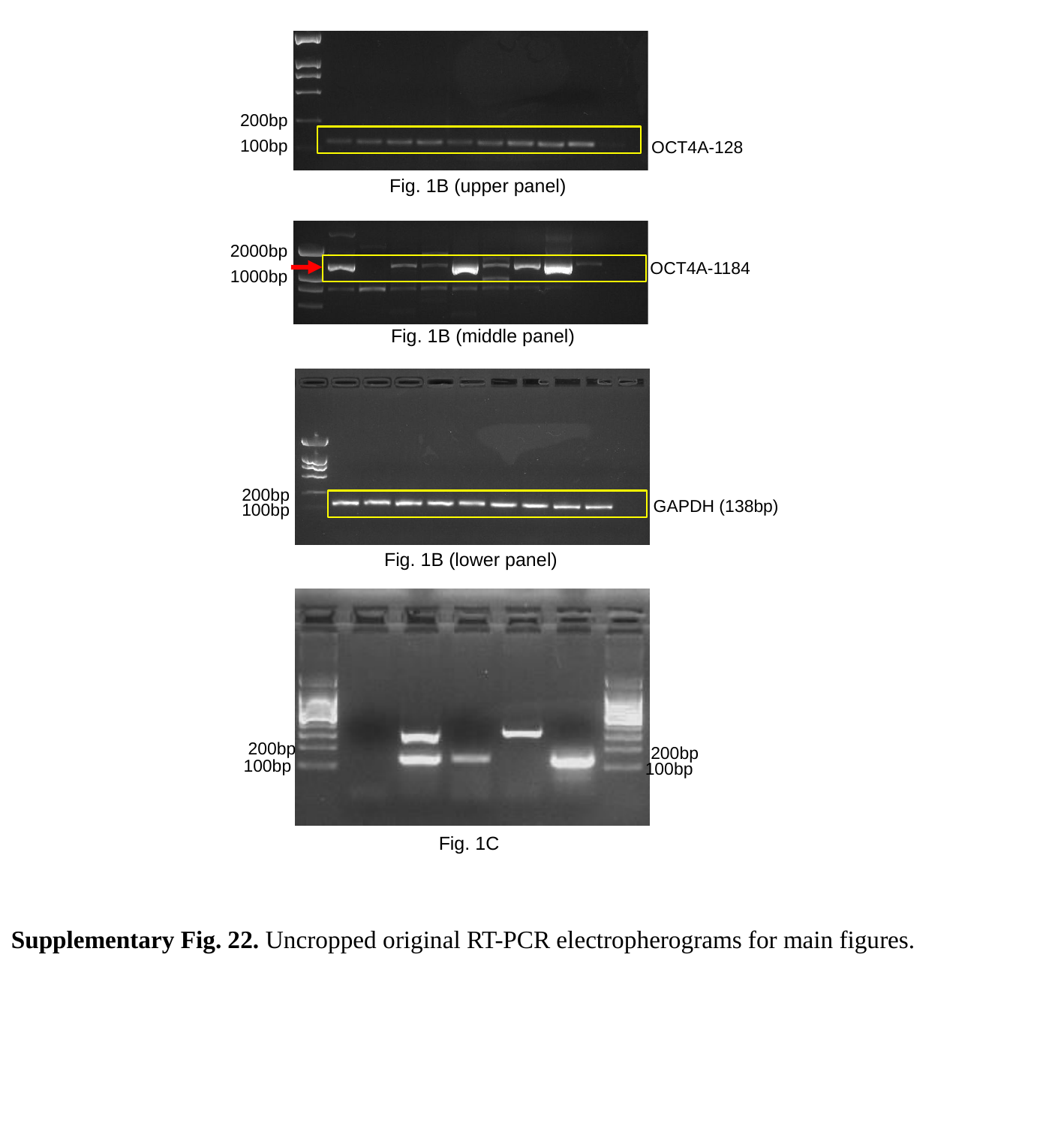

200bp
100bp
OCT4A-128
Fig. 1B (upper panel)
2000bp
OCT4A-1184
1000bp
Fig. 1B (middle panel)
200bp
GAPDH (138bp)
100bp
Fig. 1B (lower panel)
200bp
200bp
100bp
100bp
Fig. 1C
Supplementary Fig. 22. Uncropped original RT-PCR electropherograms for main figures.

## Slide 26
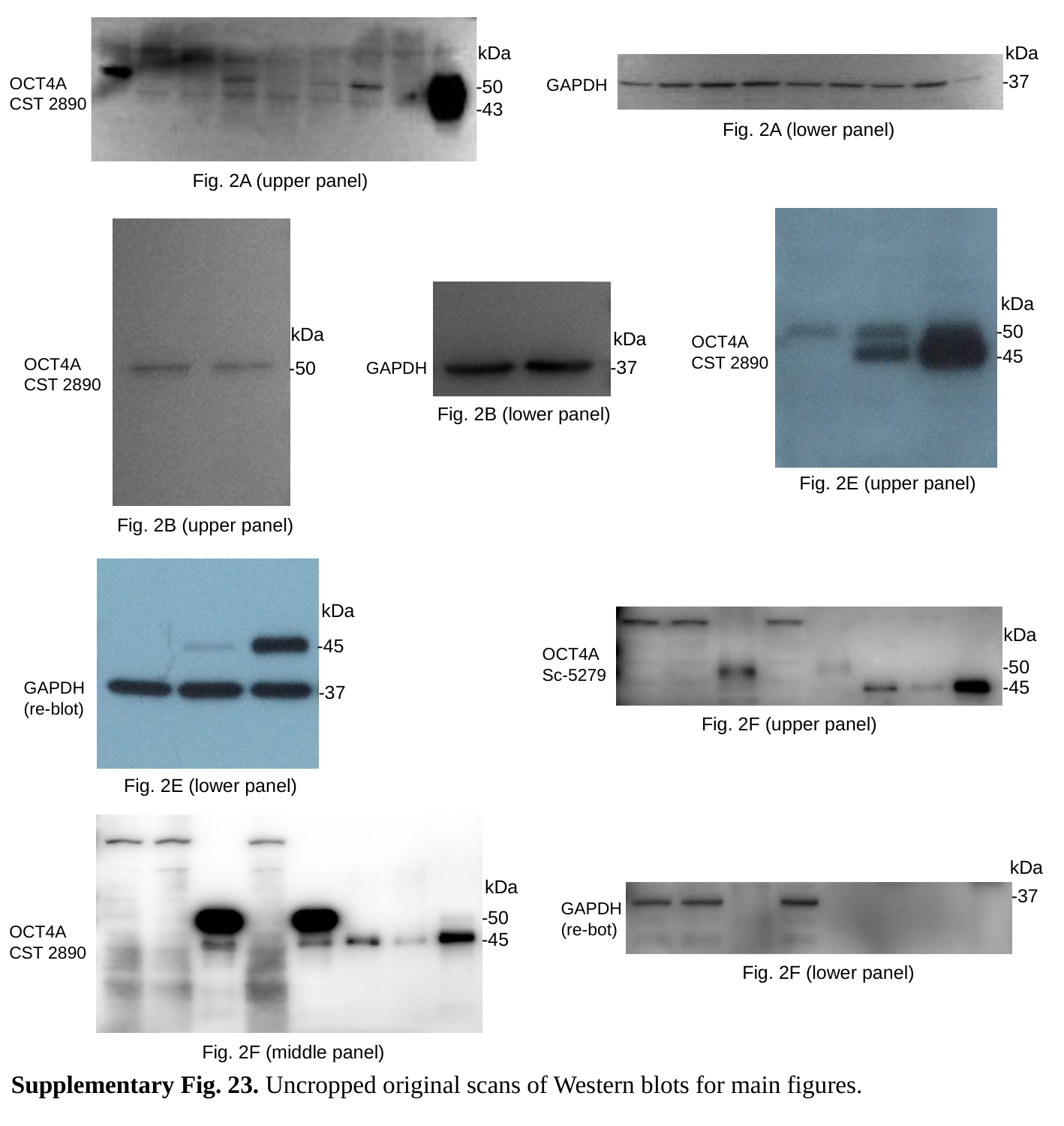

kDa
OCT4A
CST 2890
-50
-43
Fig. 2A (upper panel)
kDa
-37
GAPDH
Fig. 2A (lower panel)
kDa
-50
OCT4A
CST 2890
-45
Fig. 2E (upper panel)
kDa
OCT4A
CST 2890
-50
Fig. 2B (upper panel)
kDa
-37
GAPDH
Fig. 2B (lower panel)
kDa
-45
GAPDH
(re-blot)
-37
Fig. 2E (lower panel)
kDa
OCT4A
Sc-5279
-50
-45
Fig. 2F (upper panel)
kDa
-50
OCT4A
CST 2890
-45
Fig. 2F (middle panel)
kDa
-37
GAPDH
(re-bot)
Fig. 2F (lower panel)
Supplementary Fig. 23. Uncropped original scans of Western blots for main figures.

## Slide 27
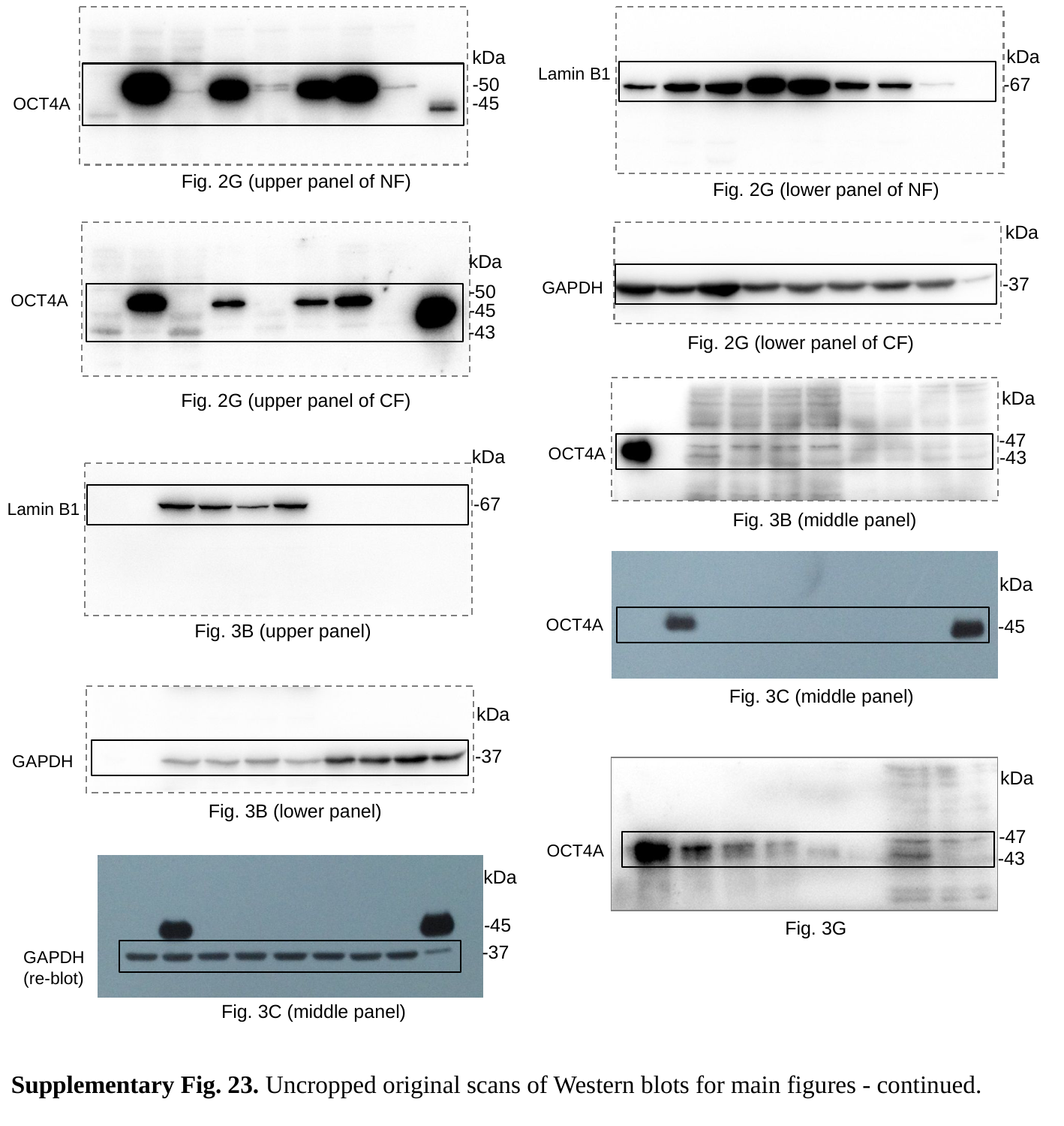

kDa
-50
-45
OCT4A
Fig. 2G (upper panel of NF)
Lamin B1
Fig. 2G (lower panel of NF)
kDa
-67
kDa
GAPDH
Fig. 2G (lower panel of CF)
-37
OCT4A
Fig. 2G (upper panel of CF)
kDa
-50
-45
-43
OCT4A
Fig. 3B (middle panel)
kDa
-47
-43
kDa
Lamin B1
Fig. 3B (upper panel)
-67
OCT4A
Fig. 3C (middle panel)
kDa
-45
GAPDH
Fig. 3B (lower panel)
kDa
-37
OCT4A
Fig. 3G
kDa
-47
-43
GAPDH
(re-blot)
Fig. 3C (middle panel)
kDa
-45
-37
Supplementary Fig. 23. Uncropped original scans of Western blots for main figures - continued.

## Slide 28
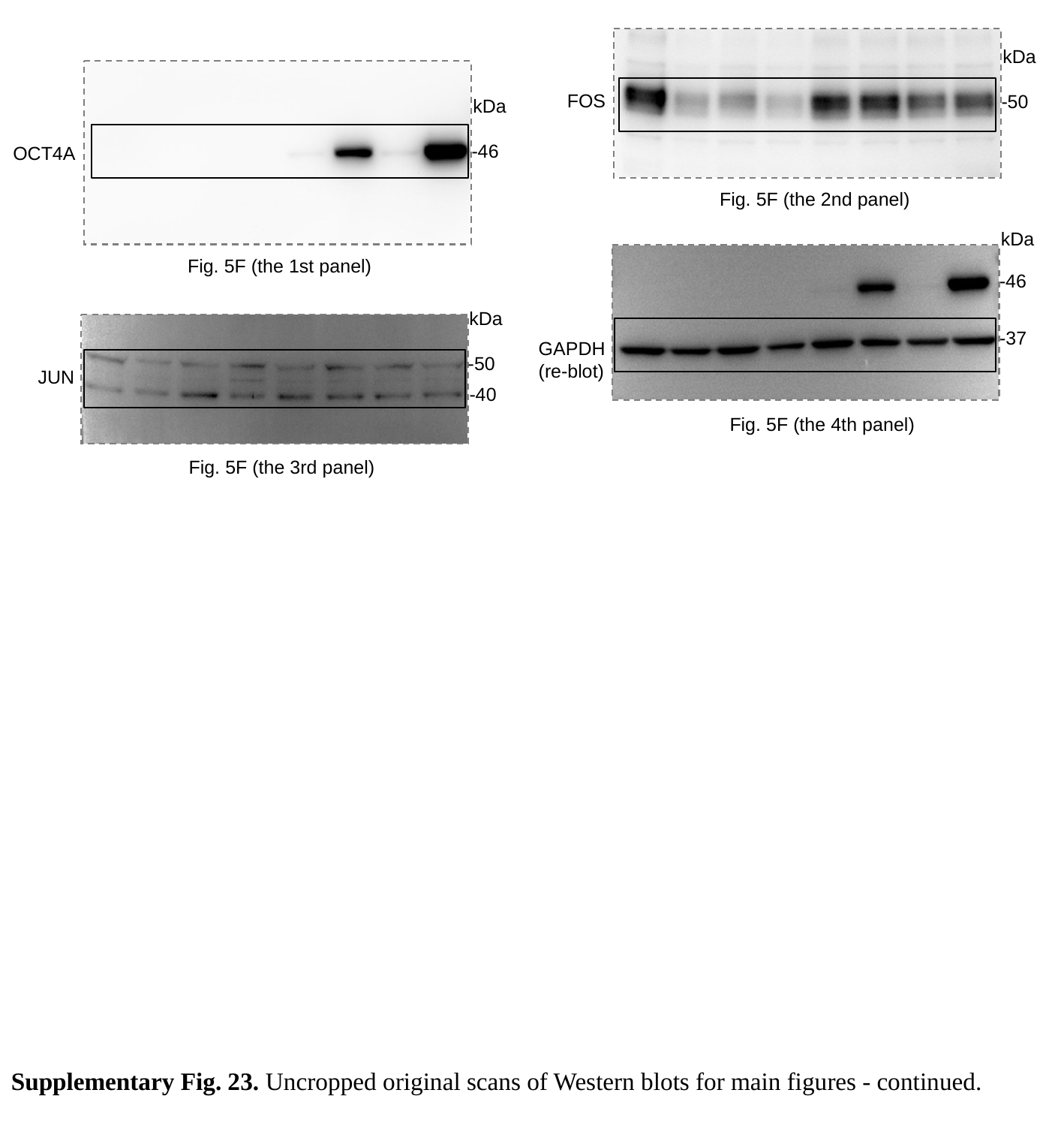

kDa
FOS
-50
Fig. 5F (the 2nd panel)
kDa
-46
OCT4A
Fig. 5F (the 1st panel)
kDa
-46
-37
GAPDH
(re-blot)
Fig. 5F (the 4th panel)
kDa
-50
JUN
-40
Fig. 5F (the 3rd panel)
Supplementary Fig. 23. Uncropped original scans of Western blots for main figures - continued.

## Slide 29
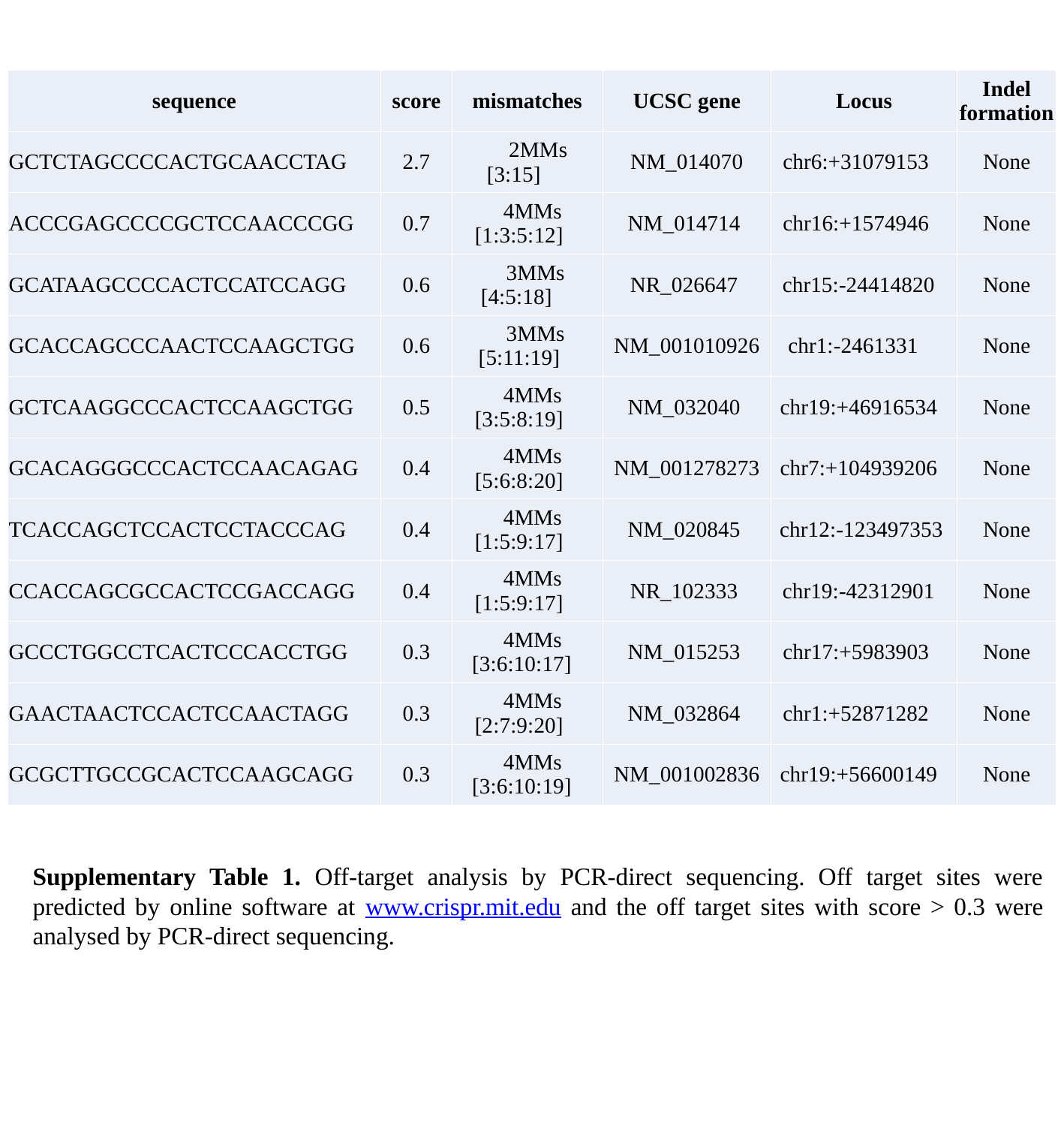

| sequence | score | mismatches | UCSC gene | Locus | Indel formation |
| --- | --- | --- | --- | --- | --- |
| GCTCTAGCCCCACTGCAACCTAG | 2.7 | 2MMs [3:15] | NM\_014070 | chr6:+31079153 | None |
| ACCCGAGCCCCGCTCCAACCCGG | 0.7 | 4MMs [1:3:5:12] | NM\_014714 | chr16:+1574946 | None |
| GCATAAGCCCCACTCCATCCAGG | 0.6 | 3MMs [4:5:18] | NR\_026647 | chr15:-24414820 | None |
| GCACCAGCCCAACTCCAAGCTGG | 0.6 | 3MMs [5:11:19] | NM\_001010926 | chr1:-2461331 | None |
| GCTCAAGGCCCACTCCAAGCTGG | 0.5 | 4MMs [3:5:8:19] | NM\_032040 | chr19:+46916534 | None |
| GCACAGGGCCCACTCCAACAGAG | 0.4 | 4MMs [5:6:8:20] | NM\_001278273 | chr7:+104939206 | None |
| TCACCAGCTCCACTCCTACCCAG | 0.4 | 4MMs [1:5:9:17] | NM\_020845 | chr12:-123497353 | None |
| CCACCAGCGCCACTCCGACCAGG | 0.4 | 4MMs [1:5:9:17] | NR\_102333 | chr19:-42312901 | None |
| GCCCTGGCCTCACTCCCACCTGG | 0.3 | 4MMs [3:6:10:17] | NM\_015253 | chr17:+5983903 | None |
| GAACTAACTCCACTCCAACTAGG | 0.3 | 4MMs [2:7:9:20] | NM\_032864 | chr1:+52871282 | None |
| GCGCTTGCCGCACTCCAAGCAGG | 0.3 | 4MMs [3:6:10:19] | NM\_001002836 | chr19:+56600149 | None |
Supplementary Table 1. Off-target analysis by PCR-direct sequencing. Off target sites were predicted by online software at www.crispr.mit.edu and the off target sites with score > 0.3 were analysed by PCR-direct sequencing.

## Slide 30
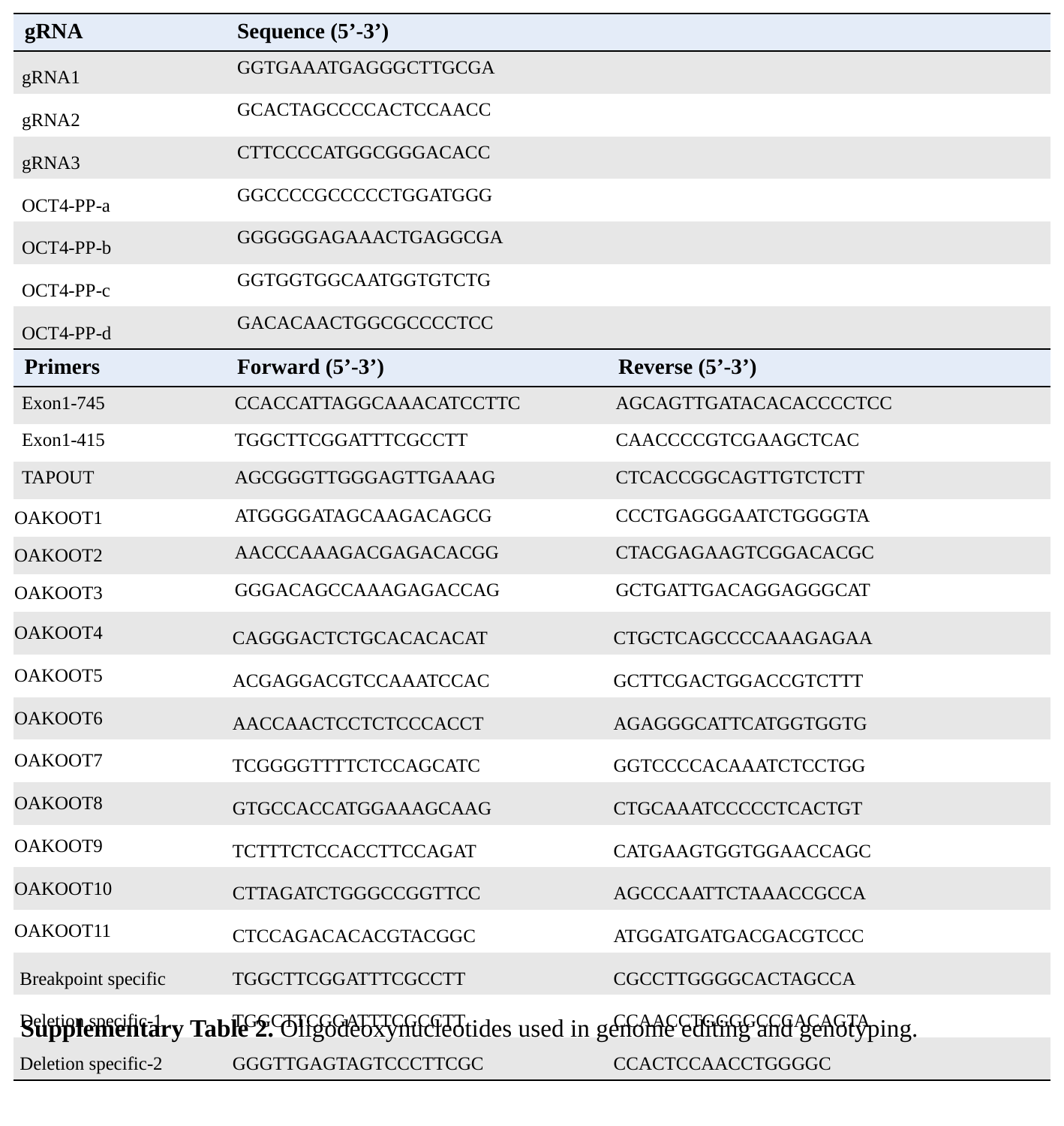

| gRNA | Sequence (5’-3’) | |
| --- | --- | --- |
| gRNA1 | GGTGAAATGAGGGCTTGCGA | |
| gRNA2 | GCACTAGCCCCACTCCAACC | |
| gRNA3 | CTTCCCCATGGCGGGACACC | |
| OCT4-PP-a | GGCCCCGCCCCCTGGATGGG | |
| OCT4-PP-b | GGGGGGAGAAACTGAGGCGA | |
| OCT4-PP-c | GGTGGTGGCAATGGTGTCTG | |
| OCT4-PP-d | GACACAACTGGCGCCCCTCC | |
| Primers | Forward (5’-3’) | Reverse (5’-3’) |
| Exon1-745 | CCACCATTAGGCAAACATCCTTC | AGCAGTTGATACACACCCCTCC |
| Exon1-415 | TGGCTTCGGATTTCGCCTT | CAACCCCGTCGAAGCTCAC |
| TAPOUT | AGCGGGTTGGGAGTTGAAAG | CTCACCGGCAGTTGTCTCTT |
| OAKOOT1 | ATGGGGATAGCAAGACAGCG | CCCTGAGGGAATCTGGGGTA |
| OAKOOT2 | AACCCAAAGACGAGACACGG | CTACGAGAAGTCGGACACGC |
| OAKOOT3 | GGGACAGCCAAAGAGACCAG | GCTGATTGACAGGAGGGCAT |
| OAKOOT4 | CAGGGACTCTGCACACACAT | CTGCTCAGCCCCAAAGAGAA |
| OAKOOT5 | ACGAGGACGTCCAAATCCAC | GCTTCGACTGGACCGTCTTT |
| OAKOOT6 | AACCAACTCCTCTCCCACCT | AGAGGGCATTCATGGTGGTG |
| OAKOOT7 | TCGGGGTTTTCTCCAGCATC | GGTCCCCACAAATCTCCTGG |
| OAKOOT8 | GTGCCACCATGGAAAGCAAG | CTGCAAATCCCCCTCACTGT |
| OAKOOT9 | TCTTTCTCCACCTTCCAGAT | CATGAAGTGGTGGAACCAGC |
| OAKOOT10 | CTTAGATCTGGGCCGGTTCC | AGCCCAATTCTAAACCGCCA |
| OAKOOT11 | CTCCAGACACACGTACGGC | ATGGATGATGACGACGTCCC |
| Breakpoint specific | TGGCTTCGGATTTCGCCTT | CGCCTTGGGGCACTAGCCA |
| Deletion specific-1 | TGGCTTCGGATTTCGCCTT | CCAACCTGGGGCCCACAGTA |
| Deletion specific-2 | GGGTTGAGTAGTCCCTTCGC | CCACTCCAACCTGGGGC |
Supplementary Table 2. Oligodeoxynucleotides used in genome editing and genotyping.

## Slide 31
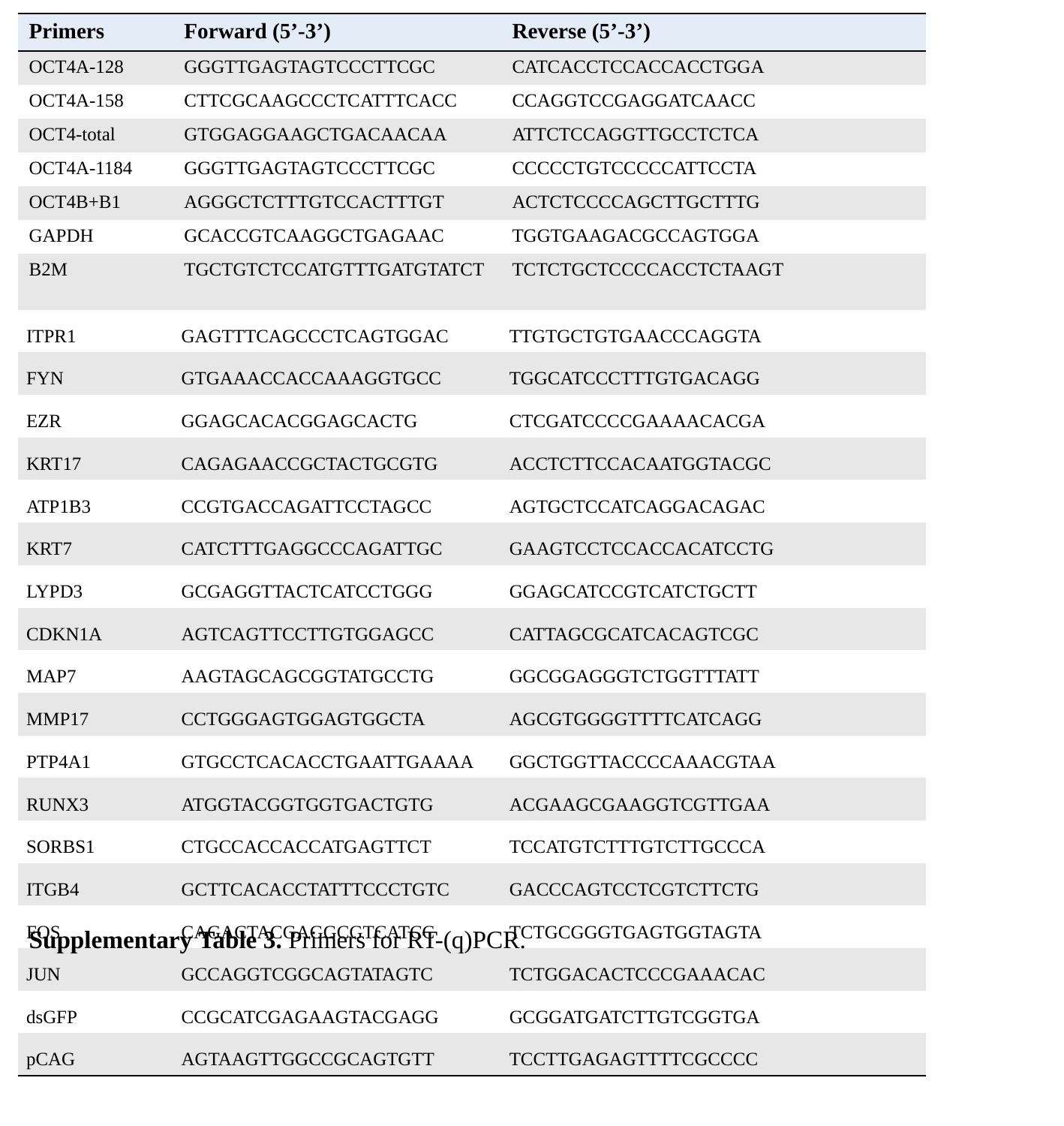

| Primers | Forward (5’-3’) | Reverse (5’-3’) |
| --- | --- | --- |
| OCT4A-128 | GGGTTGAGTAGTCCCTTCGC | CATCACCTCCACCACCTGGA |
| OCT4A-158 | CTTCGCAAGCCCTCATTTCACC | CCAGGTCCGAGGATCAACC |
| OCT4-total | GTGGAGGAAGCTGACAACAA | ATTCTCCAGGTTGCCTCTCA |
| OCT4A-1184 | GGGTTGAGTAGTCCCTTCGC | CCCCCTGTCCCCCATTCCTA |
| OCT4B+B1 | AGGGCTCTTTGTCCACTTTGT | ACTCTCCCCAGCTTGCTTTG |
| GAPDH | GCACCGTCAAGGCTGAGAAC | TGGTGAAGACGCCAGTGGA |
| B2M | TGCTGTCTCCATGTTTGATGTATCT | TCTCTGCTCCCCACCTCTAAGT |
| ITPR1 | GAGTTTCAGCCCTCAGTGGAC | TTGTGCTGTGAACCCAGGTA |
| FYN | GTGAAACCACCAAAGGTGCC | TGGCATCCCTTTGTGACAGG |
| EZR | GGAGCACACGGAGCACTG | CTCGATCCCCGAAAACACGA |
| KRT17 | CAGAGAACCGCTACTGCGTG | ACCTCTTCCACAATGGTACGC |
| ATP1B3 | CCGTGACCAGATTCCTAGCC | AGTGCTCCATCAGGACAGAC |
| KRT7 | CATCTTTGAGGCCCAGATTGC | GAAGTCCTCCACCACATCCTG |
| LYPD3 | GCGAGGTTACTCATCCTGGG | GGAGCATCCGTCATCTGCTT |
| CDKN1A | AGTCAGTTCCTTGTGGAGCC | CATTAGCGCATCACAGTCGC |
| MAP7 | AAGTAGCAGCGGTATGCCTG | GGCGGAGGGTCTGGTTTATT |
| MMP17 | CCTGGGAGTGGAGTGGCTA | AGCGTGGGGTTTTCATCAGG |
| PTP4A1 | GTGCCTCACACCTGAATTGAAAA | GGCTGGTTACCCCAAACGTAA |
| RUNX3 | ATGGTACGGTGGTGACTGTG | ACGAAGCGAAGGTCGTTGAA |
| SORBS1 | CTGCCACCACCATGAGTTCT | TCCATGTCTTTGTCTTGCCCA |
| ITGB4 | GCTTCACACCTATTTCCCTGTC | GACCCAGTCCTCGTCTTCTG |
| FOS | CAGACTACGAGGCGTCATCC | TCTGCGGGTGAGTGGTAGTA |
| JUN | GCCAGGTCGGCAGTATAGTC | TCTGGACACTCCCGAAACAC |
| dsGFP | CCGCATCGAGAAGTACGAGG | GCGGATGATCTTGTCGGTGA |
| pCAG | AGTAAGTTGGCCGCAGTGTT | TCCTTGAGAGTTTTCGCCCC |
Supplementary Table 3. Primers for RT-(q)PCR.

## Slide 32
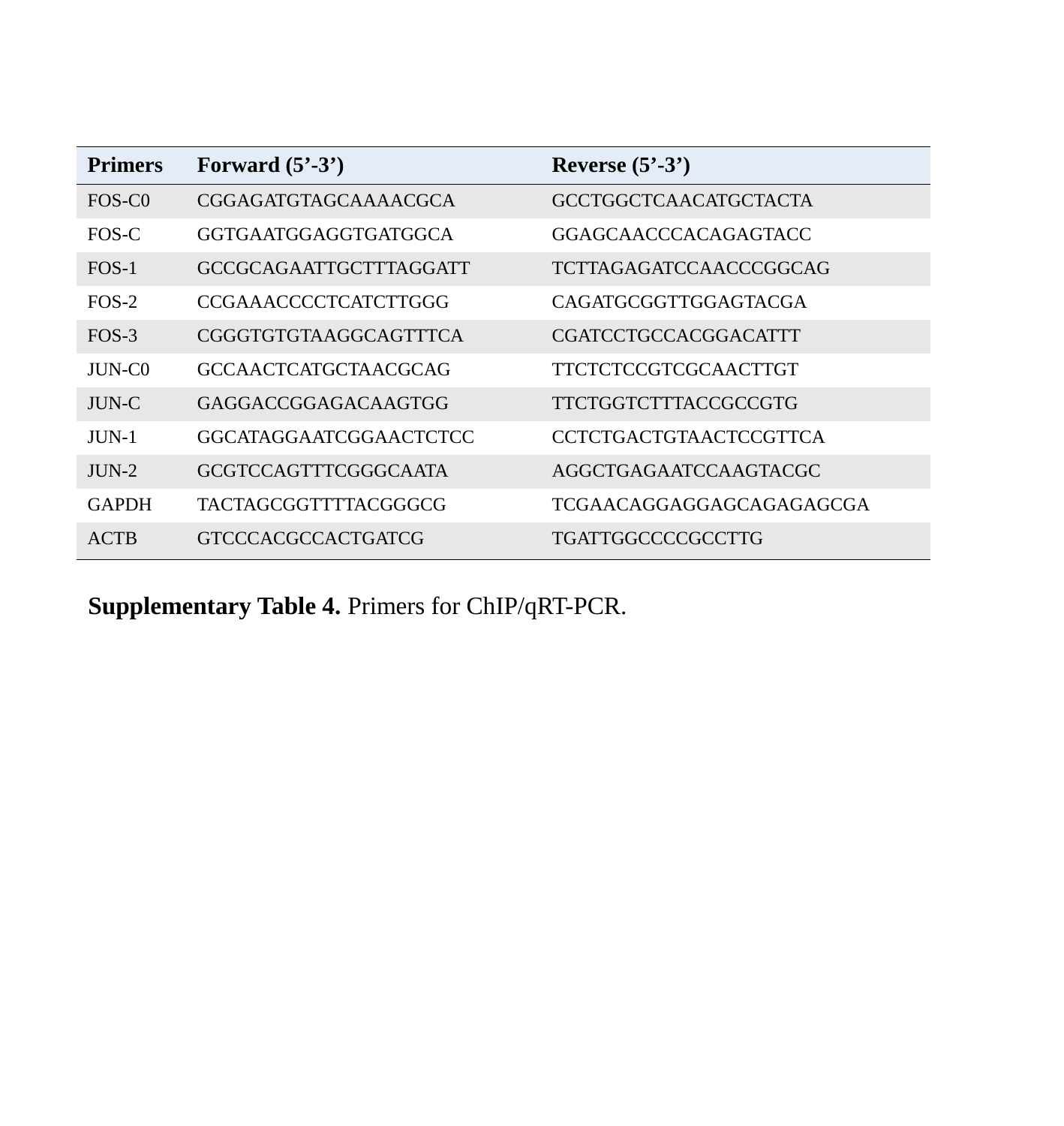

| Primers | Forward (5’-3’) | Reverse (5’-3’) |
| --- | --- | --- |
| FOS-C0 | CGGAGATGTAGCAAAACGCA | GCCTGGCTCAACATGCTACTA |
| FOS-C | GGTGAATGGAGGTGATGGCA | GGAGCAACCCACAGAGTACC |
| FOS-1 | GCCGCAGAATTGCTTTAGGATT | TCTTAGAGATCCAACCCGGCAG |
| FOS-2 | CCGAAACCCCTCATCTTGGG | CAGATGCGGTTGGAGTACGA |
| FOS-3 | CGGGTGTGTAAGGCAGTTTCA | CGATCCTGCCACGGACATTT |
| JUN-C0 | GCCAACTCATGCTAACGCAG | TTCTCTCCGTCGCAACTTGT |
| JUN-C | GAGGACCGGAGACAAGTGG | TTCTGGTCTTTACCGCCGTG |
| JUN-1 | GGCATAGGAATCGGAACTCTCC | CCTCTGACTGTAACTCCGTTCA |
| JUN-2 | GCGTCCAGTTTCGGGCAATA | AGGCTGAGAATCCAAGTACGC |
| GAPDH | TACTAGCGGTTTTACGGGCG | TCGAACAGGAGGAGCAGAGAGCGA |
| ACTB | GTCCCACGCCACTGATCG | TGATTGGCCCCGCCTTG |
Supplementary Table 4. Primers for ChIP/qRT-PCR.

## Slide 33
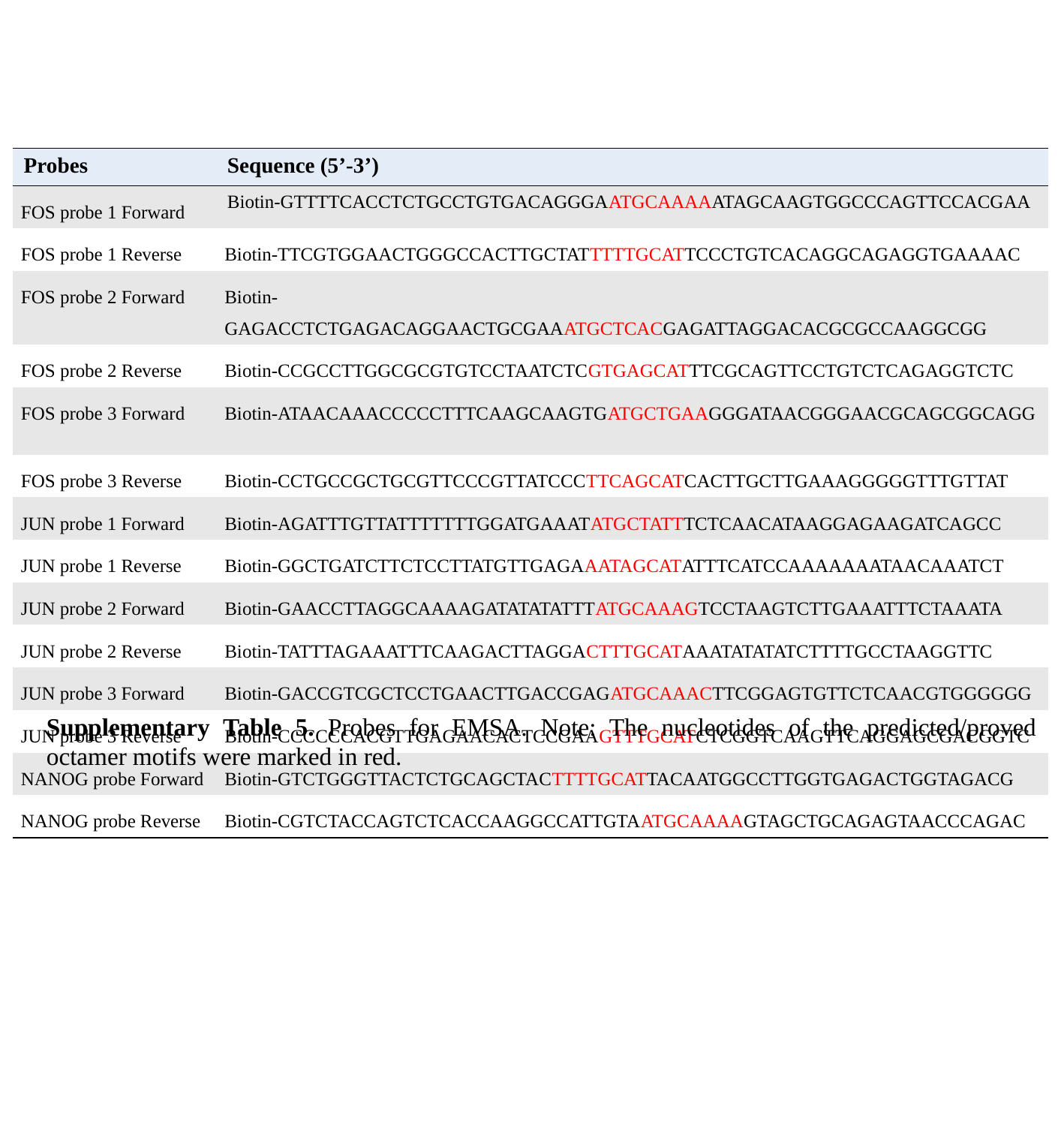

| Probes | Sequence (5’-3’) |
| --- | --- |
| FOS probe 1 Forward | Biotin-GTTTTCACCTCTGCCTGTGACAGGGAATGCAAAAATAGCAAGTGGCCCAGTTCCACGAA |
| FOS probe 1 Reverse | Biotin-TTCGTGGAACTGGGCCACTTGCTATTTTTGCATTCCCTGTCACAGGCAGAGGTGAAAAC |
| FOS probe 2 Forward | Biotin-GAGACCTCTGAGACAGGAACTGCGAAATGCTCACGAGATTAGGACACGCGCCAAGGCGG |
| FOS probe 2 Reverse | Biotin-CCGCCTTGGCGCGTGTCCTAATCTCGTGAGCATTTCGCAGTTCCTGTCTCAGAGGTCTC |
| FOS probe 3 Forward | Biotin-ATAACAAACCCCCTTTCAAGCAAGTGATGCTGAAGGGATAACGGGAACGCAGCGGCAGG |
| FOS probe 3 Reverse | Biotin-CCTGCCGCTGCGTTCCCGTTATCCCTTCAGCATCACTTGCTTGAAAGGGGGTTTGTTAT |
| JUN probe 1 Forward | Biotin-AGATTTGTTATTTTTTTGGATGAAATATGCTATTTCTCAACATAAGGAGAAGATCAGCC |
| JUN probe 1 Reverse | Biotin-GGCTGATCTTCTCCTTATGTTGAGAAATAGCATATTTCATCCAAAAAAATAACAAATCT |
| JUN probe 2 Forward | Biotin-GAACCTTAGGCAAAAGATATATATTTATGCAAAGTCCTAAGTCTTGAAATTTCTAAATA |
| JUN probe 2 Reverse | Biotin-TATTTAGAAATTTCAAGACTTAGGACTTTGCATAAATATATATCTTTTGCCTAAGGTTC |
| JUN probe 3 Forward | Biotin-GACCGTCGCTCCTGAACTTGACCGAGATGCAAACTTCGGAGTGTTCTCAACGTGGGGGG |
| JUN probe 3 Reverse | Biotin-CCCCCCACGTTGAGAACACTCCGAAGTTTGCATCTCGGTCAAGTTCAGGAGCGACGGTC |
| NANOG probe Forward | Biotin-GTCTGGGTTACTCTGCAGCTACTTTTGCATTACAATGGCCTTGGTGAGACTGGTAGACG |
| NANOG probe Reverse | Biotin-CGTCTACCAGTCTCACCAAGGCCATTGTAATGCAAAAGTAGCTGCAGAGTAACCCAGAC |
Supplementary Table 5. Probes for EMSA. Note: The nucleotides of the predicted/proved octamer motifs were marked in red.

## Slide 34
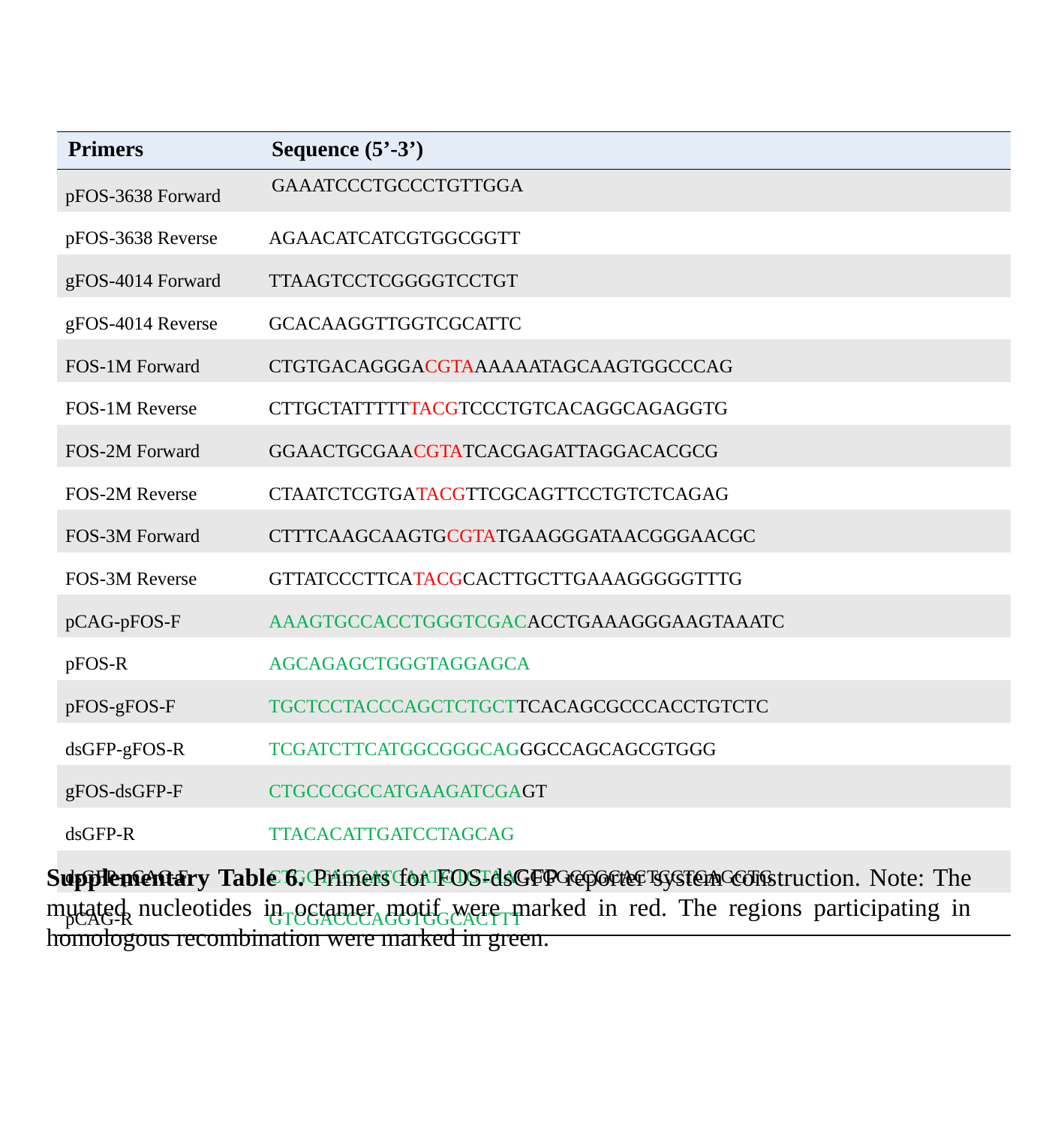

| Primers | Sequence (5’-3’) |
| --- | --- |
| pFOS-3638 Forward | GAAATCCCTGCCCTGTTGGA |
| pFOS-3638 Reverse | AGAACATCATCGTGGCGGTT |
| gFOS-4014 Forward | TTAAGTCCTCGGGGTCCTGT |
| gFOS-4014 Reverse | GCACAAGGTTGGTCGCATTC |
| FOS-1M Forward | CTGTGACAGGGACGTAAAAAATAGCAAGTGGCCCAG |
| FOS-1M Reverse | CTTGCTATTTTTTACGTCCCTGTCACAGGCAGAGGTG |
| FOS-2M Forward | GGAACTGCGAACGTATCACGAGATTAGGACACGCG |
| FOS-2M Reverse | CTAATCTCGTGATACGTTCGCAGTTCCTGTCTCAGAG |
| FOS-3M Forward | CTTTCAAGCAAGTGCGTATGAAGGGATAACGGGAACGC |
| FOS-3M Reverse | GTTATCCCTTCATACGCACTTGCTTGAAAGGGGGTTTG |
| pCAG-pFOS-F | AAAGTGCCACCTGGGTCGACACCTGAAAGGGAAGTAAATC |
| pFOS-R | AGCAGAGCTGGGTAGGAGCA |
| pFOS-gFOS-F | TGCTCCTACCCAGCTCTGCTTCACAGCGCCCACCTGTCTC |
| dsGFP-gFOS-R | TCGATCTTCATGGCGGGCAGGGCCAGCAGCGTGGG |
| gFOS-dsGFP-F | CTGCCCGCCATGAAGATCGAGT |
| dsGFP-R | TTACACATTGATCCTAGCAG |
| dsGFP-pCAG-F | CTGCTAGGATCAATGTGTAAGCGGCCGCACTCCTCAGGTG |
| pCAG-R | GTCGACCCAGGTGGCACTTT |
Supplementary Table 6. Primers for FOS-dsGFP reporter system construction. Note: The mutated nucleotides in octamer motif were marked in red. The regions participating in homologous recombination were marked in green.
