# Supplementary material for: Endogenous authentic OCT4A proteins directly regulate FOS/AP-1 transcription in somatic cancer cells
Source: Cell Death Dis. 2018 May 22;9(6):585. doi: 10.1038/s41419-018-0606-x (PMC5964179; doi:10.1038/s41419-018-0606-x)
Supplement: Supplementary file 2 — Supplemental methods [file 41419_2018_606_MOESM2_ESM.docx]

**Supplementary Materials and Methods**

**Cell lines and culture**

The HeLa, 293T, U87, U251, HepG2, Huh7, MDA-MB-231, A549, LO2 cells were obtained from the Cell Bank of the Chinese Academy of Sciences, Shanghai, China. HUVEC and NCCIT cells were obtained from ATCC. 293T, U251, Huh7, LO2, HUVEC and NCCIT cells were cultured in DMEM (Hyclone SH30243.01B), HeLa, U87 and HepG2 cells in MEM (Hyclone SH30024.01B), MDA-MB-231 cells in L15 (Hyclone SH30525.01) and A549 cells in F12K (Gibco 21127-022). All culture media were supplemented with 10% fetal bovine serum (Gibco 10099 or Pufei 1101-500) and 1% (v/v) penicillin/streptomycin (Hyclone SV30010). MDA-MB-231 cells were cultured at 37˚C with 100% air and all other cells were cultured at 37˚C with 95% air and 5% CO_2_.

**RT-PCR/qPCR analysis**

Total RNA was extracted by RNAiso Plus (TaKaRa 9109). Genomic DNA contamination was erased from total RNA first with gDNA Eraser and then the total RNA was reverse transcribed to cDNA using the PrimeScript RT reagent kit (TaKaRa RR047A) according to the manufacturer’s protocol. Regular PCR and quantitative real-time PCR (qPCR) were performed using the KOD FX reagent (TOYOBO KFX-101) and the iTaq Universal SYBR Green Supermix (Bio-Rad 1721-5124). All the primers used in regular PCR or quantitative real-time PCR are listed in Supplementary Table 4.

**Immunoprecipitation (IP) and mass spectrometric (MS) analysis**

HeLa, Tag knock-in HeLa clone 3A11 or NCCIT cells were lysed on ice in a lysis buffer for Western blot/IP (Beyotime P0013) supplemented with Pierce Protease Inhibitor Tablets (EDTA-free) (Thermo 88802). The whole cell lysates were incubated with anti-OCT4A (Cell Signaling Technology (CST) 2890) or anti-FLAG M2 beads (Sigma M8823) for indicated time at 4˚C. Immunocomplexes were eluted by low pH elution buffer (0.1 M glycine, pH 2.0) or 1.5×Laemmli buffer (Bio-Rad 161-0747) before being resolved by SDS-PAGE followed by Western blotting (WB) or Coomassie Brilliant Blue staining.

For MS analysis, the OCT4A (45 kDa) or Tag-OCT4A (50 kDa) bands were excised from gels after destaining and further digested overnight by trypsin. The peptides were extracted three times with 60% acetonitrile/0.1% trifluoroacetic acid and vacuum-dried. The peptide mixture was loaded onto a C18-reversed phase column (Thermo Scientific Easy Column, 10 cm long, 75 μm inner diameter, 3 μm resin) in buffer A (0.1% formic acid) and separated with a linear gradient of buffer B (80% acetonitrile and 0.1% Formic acid) at a flow rate of 250 nl/min controlled by IntelliFlow technology over 140 min. Experiments were performed on the Q Exactive mass spectrometer that was coupled to Easy nLC (Thermo Fisher Scientific). MS data were acquired using a data-dependent top10 method dynamically choosing the most abundant precursor ions from the survey scan (300–1800 m/z) for HCD fragmentation. Determination of the target value was based on predictive Automatic Gain Control (pAGC). Survey scans were acquired at a resolution of 70,000 at m/z 200 and resolution for HCD spectra was set to 17,500 at m/z 200. Normalized collision energy was 30 eV and the underfill ratio, which specifies the minimum percentage of the target value likely to be reached at maximum fill time, was defined as 0.1%. The instrument was run with peptide recognition mode enabled. MS/MS spectra were searched using MASCOT engine (Matrix Science, London, UK; version 2.2) against the UNIPROT human database (updated on 04/07/2016; 154485 sequences). For protein identification, the following options were used. Enzyme: Trypsin, Dynamical modifications: Oxidation (M), Fixed modification: Carbamidomethyl (C), Max Missed Cleavages: 2, ProteomicsTools: 3.1.6 and Filter by score ≥ 20.

**Inducible overexpression of OCT4A or OCT4-PG1**

Inducible FLAG:OCT4A, FLAG:OCT4-PG1 or GFP (as control) expression vectors with doxycycline (Dox) induction were constructed by replacing Cas9 in pCW-Cas9 (Addgene 50661) with FLAG-OCT4A, FLAG-OCT4-PG1, or GFP, termed pCW-FLAG:OCT4A, pCW-FLAG:OCT4-PG1 or pCW-GFP, respectively. To generate 293T cells with inducibly expressed OCT4-PG1, the pCW-FLAG:OCT4-PG1 or pCW-GFP (used as negative control) plasmids were transiently transfected into 293T cells, respectively. Twenty-four hours after transfection, the transfected cells were treated with or without 1 μg/ml Dox and further cultured for 24 h before being subjected for further analysis. To create HeLa cells with inducibly expressed OCT4A, the recombinant plasmid pCW-FLAG:OCT4A was packaged into lentiviral pseudoparticles. OCT4A-KO (2-2) or wild type (WT) HeLa cells were infected with lentiviruses at low MOI and selected in 1 μg/ml puromycin for 7 days. The remaining cells were propagated and subjected for further analysis.

**Endogenous OCT4A quantification in cancer cells**

First, the His-OCT4A proteins were quantitated by Coomassie Brilliant Blue method. Briefly, serially diluted BSA (as standard) and His-OCT4A proteins were subjected to SDS-PAGE and stained by Coomassie Brilliant Blue R-250 (Sangon A100472). After destaining, images were captured by the Gel Doc XR+ System (Bio-Rad) and further analyzed by Image J. The gray values of the protein bands and their equivalent mass of BSA were fit into a linear equation as the standard curve and then the gray values of the His-OCT4A bands with the correct size were analyzed by Image J. The relevant mass of each His-OCT4A band was calculated through the BSA standard curve and the exact concentration of His-OCT4A was obtained by dividing the mass with the volume loaded. Then, nuclear proteins were extracted from 1.6 × 10^6^ HeLa and NCCIT cells which were counted by the Countess II FL Automated Cell Counter (Thermo). Serially diluted His-OCT4A proteins (as standard), HeLa nuclear proteins and diluted NCCIT nuclear proteins were subjected to SDS-PAGE on the same gel and enhanced WB analysis. The signals of bands recognized by anti-OCT4A (CST 2890) were visualized and the images were acquired by a Tanon 5200 Multi Imaging System. The gray values of the bands of His-OCT4A (46 kDa), the band of NCCIT (45 kDa) and the band of HeLa (about 45 kDa that was lacking in Clone A2) were analyzed by Image J. The gray values and the equivalent molar numbers of the His-OCT4A bands were fitted to a polynomial equation to generate a standard curve and then the molar numbers (n, in the range of 10^-16^ Mol) corresponding to the OCT4A bands of HeLa nuclear proteins and NCCIT nuclear proteins, respectively, were calculated based on the gray values through the standard curve of His-OCT4A. The OCT4A protein molecule numbers (N) equivalent to the indicated bands were determined by the formula N = n × N_A_ where “n” stands for mole number and “N_A_” stands for Avogadro constant (6.02 × 10^23^). Finally, the OCT4A protein number/nucleus of HeLa and NCCIT was estimated by normalization with sample dilution factors and harvested cell numbers (OCT4A protein number/cell nucleus = N/dilution factor/cell number).

**Immunofluorescence microscopy**

Tag**-**OCT4A cells were seeded on 35 mm glass bottom dishes for 48 h, washed twice with PBS, fixed in 4% paraformaldehyde (Sangon E672002) for 15 min, washed twice with PBS, permeabilized with 0.2% Triton X-100 at RT for 10 min, washed three times with PBS and blocked in 5% Donkey Serum (Jackson ImmunoResearch 017-000-121) in PBS for 45 min. Two primary antibodies (Anti-OCT4A, CST 2890; Anti-FLAG M2, Sigma F1804), diluted at 1:200 ratio were used to incubate cells separately at 4°C overnight. Next day, the cells were washed for three times with PBST, incubated with species-specific AlexFluo conjugated secondary antibodies (Jackson ImmunoResearch) at a dilution ratio of 1:200, washed three times with PBST, counterstained with Hoechst 33342 (Molecular Probe H3570) and inspected under a confocal Microscopy (Olympus FV1000).

**RNA-Seq analysis**

Briefly, total RNA was isolated from WT and OCT4A-KO (2-2) HeLa cells using RNAiso Plus reagent (TaKaRa 9109) following the manufacturer’s procedures. Approximately 10 μg of total RNA were subjected to deplete ribosomal RNA according to the instructions of the Epicentre Ribo-Zero Gold Kit (Illumina RZH1046). Then both the poly(A)- or poly(A)+ RNA fractions were fragmented into small pieces and the cleaved RNA fragments were reverse-transcribed to create the final cDNA library as guided by the procedure for the mRNA-Seq sample preparation kit (Illumina), the average insert size for the paired-end libraries was about 300 bp. The paired-end sequencing was performed on an Illumina Hiseq2000/2500. RNA-seq reads of WT and 2-2 were aligned to the ENSEMBL ([ftp://ftp.ensembl.org/pub/current fasta/homo sapiens/dna/](ftp://ftp.ensembl.org/pub/current%20fasta/homo%20sapiens/dna/)) human reference genome (version: GRCH38) using Tophat package. The aligned read files were processed by Cufflinks and the transcript abundance was reflected by Fragment Per Kilobase of exon per Million fragments mapped (FPKM).

**Electrophoretic Mobility Shift Assay (EMSA)**

EMSAs were carried out using the LightShift chemiluminescent EMSA kit (Thermo 20148) as instructed by the manufacturer and the detailed procedures were provided in our previously published work (1). A 10 μl reaction system was adopted and 1 μg His-OCT4A was included to react with 5’-biotin end-labeled dsDNA probes relevant to indicated regions of the *FOS* and *JUN* gene. The sequences of the probes were listed in Supplementary Table 5.

**Chromatin immunoprecipitation (ChIP)**

ChIP assay was performed using an anti-OCT4A antibody (CST 2890) and the EZ-ChIP Chromatin Immunoprecipitation Kit (Millipore 17-371) according to the manufacturer’s procedures. Overall, 2 × 10^7^ of HeLa-WT and Tag-OCT4A (3A11) HeLa cells were covalently crosslinked by formaldehyde with a final concentration of 1% for 10 min at room temperature. The reaction was terminated by adding glycine to a final concentration of 125 mM. Cells were collected by a silicon scraper and resuspended in the SDS Lysis Buffer containing 1 × Protease Inhibitor Cocktail II and then were sonicated to shear crosslinked chromatin to 200-1000 bp fragments. 100 μl aliquot of the lysates’ supernatant were used for the subsequent IP procedure and 1% of the supernatant used in IP was used as Input. IP was performed with 5 μl anti-OCT4A (CST 2890) or other indicated antibodies at 4˚C overnight with gentle rotation. The antibody/chromatin complex was pulled down by the Protein G Agarose and washed sequentially by a series of buffers provided by the ChIP Kit. Chromatin fragments from each reaction were eluted by 200 μl Elution Buffer (1% SDS, 100 mM NaHCO_3_) and further reverse crosslinked by high concentration of NaCl, supplemented with RNase A and Proteinase K to free DNA. Finally, the freed DNA fragments were purified by a spin column-based method according to the manual of the ChIP Kit. The primers used to amplify the DNA fragments were listed in Supplementary Table 4. Regular PCR and quantitative real-time PCR (qPCR) were carried out using KOD FX reagent (TOYOBO KFX-101) and iTaq Universal SYBR Green Supermix (Bio-Rad 1721-5124), respectively. For qPCR, the DNA fragments corresponding to specific genes were quantified and expressed as percentages of their total input DNA fragments. Besides the control IgG, analysis of fragments of *ACTB*, *FOS* and *JUN* containing no putative octamer motif served as an additional control.

**Generation of OCT4A-KO somatic cancer cells**

Single guide RNA (sgRNA) solely targeting OCT4A was designed by an online software (www.crispr.mit.edu) and its specificity was verified by multiple alignment of OCT4A and OCT4A-related pseudogenes. The ordered single-stranded oligodeoxynucleotides (ssODNs) encoding gRNA were annealed and cloned into pX459 (Addgene 62988) via Golden Gate Assembly to obtain a single plasmid named ‘Cas9-OCT4A-KO’ encoding sgRNA Cas9 nuclease and puromycin resistance gene. Approximately 200,000-300,000 HeLa cells were transfected with 3 μg Cas9-OCT4A-KO plasmid by lipofactamine 3000 (Invitrogen L3000015) and the KO efficiency at the cell population level was tested by standard Surveyor Assay (IDT 706020). After a brief (48 h) puromycin enrichment of lipo-transfected cells, single cells were plated in five 96 well plates by limiting dilution method. Single cell derived clones were identified, expanded and genotyped via PCR-direct sequencing, PCR-clone sequencing, allelic specific PCR, and PCR-RFLP.

**Generation of OCT4A-tagged somatic cancer cells**

Given the nature of highly sequence similarities between OCT4A and its pseudogenes, Cas9-double nickases based gene targeting strategy was applied. In brief, two specific sgRNAs around translation start site were selected and cloned into pX462 (Addgene 62987), respectively. The HDR donor was constructed based on the 3×FLAG-2×STREP donor (Addgene 68375). 1 μg Cas9-DN and 2 μg HDR donor were co-transfected into 300,000 Hela cells, after 8 h incubation, media were refreshed with 1 μM Scr7 inhibitor (Selleck S7742) and incubated for an additional 48 h before puromycin selection. Single cell derived clones were identified, expanded and genotyped. Those with correctly tagged alleles (generally designated as Tag-OCT4A clones) and free of off-target mutations, were subjected for further analysis.

**FOS-dsGFP reporter assays**

The human *FOS* promoter (-3423～+215, relative to TSS) and *FOS* gene region (-549～+3465) were amplified from the genomic DNA of H1 cells by PCR using pFOS-3638 and gFOS-4014 primers, respectively. The two fragments were cloned into the pEasy-blunt vectors (TransGen CB111-01), designated as the pEasy-pFOS and pEasy-gFOS. Then, three mutants of the pEasy-pFOS or pEasy-gFOS targeting three distinct octamer motifs were generated by reverse PCR amplification with the FOS-1M, FOS-2M and FOS-3M primers, respectively. Therefore, the five intermediate donor vectors (pEasy-pFOS-WT/1M/2M and pEasy-gFOS-WT/3M) were constructed. Finally, the wild type and mutant FOS-dsGFP reporter genes were constructed by *in vitro* recombining the following four fragments harboring homologous sequences which were prepared by PCR with indicated primers using the CloneExpress MultiS One Step Cloning Kit (Vazyme C113-02): a promoter fragment containing a 2531 bp *FOS* promoter/enhancer region amplified by pCAG-pFOS-F and pFOS-R, a gene fragment containing the entire four exons and three introns of the *FOS* gene by pFOS-gFOS-F and dsGFP-gFOS-R, a fragment containing dsGFP CDS by gFOS-dsGFP-F and dsGFP-R, a backbone fragment containing a poly A terminator by dsGFP-pCAG-F and pCAG-R, using pEasy-pFOS-WT/1M/2M, pEasy-gFOS-WT/3M, a SORE6-dsGFP plasmid from the Wakefield Lab (2) and pCAG-ERT2CreERT2 (Addgene 13777) as a template, respectively.

The FOS-dsGFP-WT/1M/2M/3M plasmids were delivered into HeLa cells (2-2, WT, 2-2-i and 2-2-i+Dox) using the Lipofectamine LTX & PLUS Reagent (Invitrogen 15338-100). The dsGFP transcripts were quantified by RT-qPCR and adjusted by pCAG primers that target the plasmid backbone for normalizing the transfection efficiency differences. The sequences of the primers were listed in Supplementary Table 6.

**Wound healing assay**

Wild type (WT) and OCT4A-KO HeLa cells were seeded into two separated chambers generated by the Culture-Inserts (Ibidi 80206) and allowed to grow into confluency overnight. The Culture-Inserts were gently removed by using sterile tweezers and then a cell-free gap (“wound”) of about 500 μm was so formed. Three wounds were generated for each cell group. All cells were cultured in serum-free medium thereafter and images were captured at 0, 24, 48, 72 and 96 h. Area percentage of healing was analyzed by Image J software.

**Transwell migration assay**

WT and OCT4A-KO HeLa Cells were starved overnight, trypsinized and resuspended in serum-free medium. 1 × 10^5^ cells were seeded in upper chamber of the Falcon Cell Culture Inserts (8.0 µm pore size; Corning 353097). Culture medium supplemented with 20% FBS was added to the lower chamber. Twenty-four hours later, non-migrated cells in upper chamber were removed by a cotton swab, and cells passed through the pores and attached on the lower surface of the membrane were fixed with methanol and stained with crystal violet. Ten fields of each cell group were snapped under an Olympus IX81 microscope with an Olympus IX-TVAD camera and the numbers of migrated cells were counted.

**Cell propagation and cell cycle analysis**

1 × 10^5^ of each indicated cells were seeded in 6 cm culture dishes and cells were trypsinized and counted using the Countess II FL Automated Cell Counter (Thermo) at 24, 48, 72 and 96 h after seeding. Growth curves were plotted using the GraphPad Prism 5.0 software. Cell cycle analysis was carried out using a PI staining based Cell cycle and Apoptosis Analysis Kit (Beyotime C1052) as instructed by the manufacturer. Briefly, WT, 2-2 and 1-1 cells were starved overnight and re-cultured in FBS-containing culture medium for 21 h. Cells were harvested by trypsin, fixed with 70% ethanol at 4˚C overnight and then stained with PI at 37˚C for 30 min in the dark and finally analyzed by flow cytometry (Cytomics FC 500 MCL, Beckman Coulter, USA).

**Chemosensitivity and apoptosis assays**

Chemosensitivity was evaluated by IC_50_. Cells were seeded at a density of 5,000 cells in a volume of 200 μl per well in 96-well plates and cultured overnight. Then medium was replaced with fresh culture medium containing gradiently diluted Akti-1/2 or Cisplatin. The cells were cultured with drugs for 48 h and then subjected to CCK-8 analysis. The OD_450_ values were measured by a microplate reader (Bio-Rad). Inhibition rate (IR) was calculated according to the formula IR = (OD_450_ (control)-OD_450_ (drug))/OD_450_ (control) × 100% and the IC_50_ values were further calculated based on IRs through regression analysis with Probit Analysis using SPSS 22.0.

Cell apoptosis was assayed with the PE Annexin V Apoptosis Detection Kit I (BD 559763) according to the manufacturer’s instructions. WT and OCT4A-KO (2-2 and 1-1) HeLa cells were subjected to vehicle (DMSO) and Cisplatin, respectively for 48 h. Cells were washed with cold PBS and then re-suspended in 1 × Binding Buffer at a concentration of 1 × 10^6^ cells/ml and 200 μl of the cell suspensions were transferred to 1.5 ml EP tubes. And then cells were stained by PE and 7-AAD for 15 min at room temperature in the dark. After the addition of 1 × Binding Buffer to each tube, the cells were analyzed by flow cytometry (Cytomics FC 500 MCL, Beckman Coulter, USA).

**Colony formation analysis**

For plate colony formation, cells were plated at a number of 100 cells/well in 6-well plates and further cultured for two weeks with culture medium renewed every 3 days. The grown colonies were fixed with methanol, stained with Giemsa, imaged and then counted using Image J. For soft agar colony formation, 0.6% bottom layer of agarose dissolved by culture medium was plated on 6-well plates first, after being solidified, 0.4% upper layer of agarose containing 100 cells per well was plated directly on the bottom layer. 500 μl culture medium was covered on the top of the upper layer and replenished every 3 days. After two weeks, visible colonies were imaged by an Olympus IX81 microscope with an Olympus IX-TVAD camera and counted. For tumorsphere formation, cells were plated at a density of 100 cells/ml in 12-well plates with stem cell medium (SCM) as described previously (3,4). The SCM was replenished every 3 days. Images of the formed tumorspheres were captured by microscope after cells were cultured for two weeks.

**Supplemental References**

1. Pan X, Cang X, Dan S, Li J, Cheng J, Kang B*, et al.* Site-specific Disruption of the Oct4/Sox2 Protein Interaction Reveals Coordinated Mesendodermal Differentiation and the Epithelial-Mesenchymal Transition. The Journal of biological chemistry **2016**;291:18353-69

2. Tang B, Raviv A, Esposito D, Flanders KC, Daniel C, Nghiem BT*, et al.* A flexible reporter system for direct observation and isolation of cancer stem cells. Stem cell reports **2015**;4:155-69

3. Cheng J, Li W, Kang B, Zhou Y, Song J, Dan S*, et al.* Tryptophan derivatives regulate the transcription of Oct4 in stem-like cancer cells. Nature communications **2015**;6:7209

4. Zhao QW, Zhou YW, Li WX, Kang B, Zhang XQ, Yang Y*, et al.* Aktmediated phosphorylation of Oct4 is associated with the proliferation of stemlike cancer cells. Oncology reports **2015**;33:1621-9
